# Bioinformatics Analysis of Human Papillomavirus 16 Integration in Cervical Cancer: Changes in MAGI-1 Expression in Premalignant Lesions and Invasive Carcinoma

**DOI:** 10.3390/cancers16122225

**Published:** 2024-06-14

**Authors:** Oscar Catalán-Castorena, Olga Lilia Garibay-Cerdenares, Berenice Illades-Aguiar, Rocio Castillo-Sánchez, Ma. Isabel Zubillaga-Guerrero, Marco Antonio Leyva-Vazquez, Sergio Encarnacion-Guevara, Eugenia Flores-Alfaro, Mónica Ramirez-Ruano, Luz del Carmen Alarcón-Romero

**Affiliations:** 1 Cytopathology and Histochemistry Research Laboratory, Faculty of Chemical and Biological Sciences, Autonomous University of Guerrero, Chilpancingo 39070, Guerrero, Mexico; 07038467@uagro.mx (O.C.-C.); 18170@uagro.mx (M.I.Z.-G.); 2 Molecular Biomedicine Laboratory, Faculty of Chemical-Biological Sciences, Autonomous University of Guerrero, Chilpancingo 39070, Guerrero, Mexico; billades@uagro.mx (B.I.-A.); 02313@uagro.mx (M.A.L.-V.); 3 CONAHCyT-Autonomous University of Guerrero, Chilpancingo 39070, Guerrero, Mexico; 4 Cell Biology Department, CINVESTAV-IPN Research Institute, Ciudad de México 07360, Mexico; rocio.castillo@cinvestav.mx; 5 Center for Genomic Sciences, National Autonomous University of Mexico, Cuernavaca 62210, Morelos, Mexico; encarnac@ccg.unam.mx; 6 Clinical and Molecular Epidemiology Research Laboratory, Faculty of Chemical-Biological Sciences, Autonomous University of Guerrero, Chilpancingo 39070, Guerrero, Mexico; eugeniaflores@uagro.mx; 7 Functional Genomics and Proteomics Laboratory, Faculty of Chemical-Biological Sciences, Autonomous University of Guerrero, Chilpancingo 39070, Guerrero, Mexico; mramirezru@conahcyt.mx

**Keywords:** premalignant lesions, invasive squamous cell carcinoma, HPV 16, viral integration, interaction networks, MAGI-1

## Abstract

**Simple Summary:**

This research aimed to identify, through an in silico approach using interaction networks, a potential biomarker associated with HPV 16 integration in cervical cancer. MAGI-1 was selected, and the expression profiles and changes in subcellular localization in cell lines and samples were evaluated in patients with premalignant lesions and invasive squamous carcinoma with HPV 16 in an integrated state. MAGI-1 is a potential biomarker that differentiates a normal cell with integrated HPV 16 from early cervical lesions.

**Abstract:**

HPV 16 integration is crucial for the onset and progression of premalignant lesions to invasive squamous cell carcinoma (ISCC) because it promotes the amplification of proto-oncogenes and the silencing of tumor suppressor genes; some of these are proteins with PDZ domains involved in homeostasis and cell polarity. Through a bioinformatics approach based on interaction networks, a group of proteins associated with HPV 16 infection, PDZ domains, and direct physical interaction with E6 and related to different hallmarks of cancer were identified. MAGI-1 was selected to evaluate the expression profile and subcellular localization changes in premalignant lesions and ISCC with HPV 16 in an integrated state in cervical cytology; the profile expression of MAGI-1 diminished according to lesion grade. Surprisingly, in cell lines CaSki and SiHa, the protein localization was cytoplasmic and nuclear. In contrast, in histological samples, a change in subcellular localization from the cytoplasm in low-grade squamous intraepithelial lesions (LSIL) to the nucleus in the high-grade squamous intraepithelial lesion (HSIL) was observed; in in situ carcinomas and ISCC, MAGI-1 expression was absent. In conclusion, MAGI-1 expression could be a potential biomarker for distinguishing those cells with normal morphology but with HPV 16 integrated from those showing morphology-related uterine cervical lesions associated with tumor progression.

## 1. Introduction

Cervical cancer (CC) is the fourth most frequent gynecologic malignancy worldwide, with 630,000 new cases and 342,000 deaths in 2020 [1], increasing in incidence in women of reproductive age [2]. The development and progression of CC are multifactorial, with persistence and integration of high-risk HPVs (HR-HPVs) being the central factors underlying the progression of dysplastic premalignant lesions to invasive squamous cell carcinoma (ISCC) [3]. It is recognized that most HR-HPV infections are transient and revert in less than two years [4]. However, the persistence of HPV 16 infection increases the risk of progression of premalignant lesions to invasive carcinoma due to the integration of the viral genome into the host cell genome [5], which induces overexpression of its E6/E7 oncoproteins, deregulating the cell cycle and thereby increasing deregulated cell proliferation, cell death avoidance, angiogenesis activation, activation of metastatic phenotype, migration, and invasion [6]. Although integration is not part of the normal life cycle of HPV 16, as in the case of retroviruses, which possess an integrase that promotes the insertion of their genome into the host cell genome [7], for HPV 16 integration to occur, there must be damage to the DNA of both the host cell and the virus that can be produced via exposure to reactive oxygen and nitrogen species (ROS/NOS) generated as part of E1/E2-mediated viral replication [8]; during this process, the HPV 16 genome binds to host-cell DNA through the formation of the E2 complex with bromodomain protein 4 (E2-BRD4), a chromatin reader protein that recognizes and binds to acetylated histones through cell division, regulating transcription at common fragile sites [9]. BRD4 has been described to activate viral transcription at this integration site, favoring overexpression of E6/E7 oncoproteins, leading to the formation of an oncogenesis-promoting element [10]. Damage to host-cell DNA can be repaired by activating the DNA damage response (DDR) mechanism [11]. However, the E6 oncoprotein favors the fact that the damage is not repaired through the degradation of p53, which senses or recognizes the cleavage sites [12].

It has been observed that E6 interacts with more than 200 proteins, altering processes such as proliferation, inhibition of apoptosis, and cell-polarity-associated proteins involved in the epithelial–mesenchymal transition [13]. A feature of the E6 oncoprotein of HPV 16 is the presence of a recognition motif for proteins with PDZ domains (PBM) at its C-terminal end constituted by four amino acids (“ETQV/L”); this motif differs between viral genotypes 16 and 18 by one amino acid at position 151 [14]. The PBM motif in the E6 protein of HR-HPVs is considered a molecular signature of oncogenic potential in HR-HPVs [15]. Some of the proteins with these domains have been shown to have direct physical interaction with the E6 of HPV 16/18 via proteins such as DLG-1 [16], MAGI-1 [17], PTPN4 [18], and PTPN3 [19].

MAGI-1 is a protein that interacts with proteins that are part of the complex of tight junctions and adherens junctions [20], maintaining cell polarity integrity. It is considered a tumor suppressor protein [21], as loss of its expression in human T-cell leukemia [22], colorectal cancer [23], breast cancer [24], gastric cancer [25], hepatocellular cancer [26], and cervical cancer [27] is associated with the metastasis of primary tumors, increased cell proliferation, and evasion of apoptosis, modulating the activity of oncogenic pathways such as the PI3K/AKT and the Wnt/β-catenin pathways [28].

The E6 of HPV 16 induces the degradation of MAGI-1 through the junctions with the PDZ2 domain; a consequence of this degradation induces epithelial hyperplasia [21]. From an exhaustive analysis of the information in different experimental articles, 1434 proteins related to HPV-16 integration in cervical cancer were found, of which the type of integration, the site of insertion, and the frequent site of disruption of the viral genome were analyzed, as well as the genes and the altered molecular processes associated with viral carcinogenesis. MAGI-1 was selected because it is a protein associated with HPV 16 infection, viral integration, and physical interaction with E6. However, there is little experimental evidence regarding its expression and behavior during cervical carcinogenesis progression. Our study evaluated the changes in the expression profile and subcellular localization in cell lines, premalignant lesions, and ISCC with HPV 16 integration. The profile expression of MAGI-1 diminished according to the lesion grade. Surprisingly, in cell lines CaSki and SiHa, the protein localization was cytoplasmic and nuclear. However, a gradual change in subcellular localization from the cytoplasm to the nucleus was observed according to the premalignant lesion grade, such as low-grade squamous intraepithelial lesions (LSIL) and high-grade squamous intraepithelial lesions (HSIL). Still, in in situ carcinomas and ISCC, the MAGI-1 expression is absent. In conclusion, MAGI-1 expression could be a potential biomarker in distinguishing those cells with normal morphology but with HPV 16 integrated from those showing morphology related to premalignant cervical lesions associated with tumor progression.

## 2. Materials and Methods

### 2.1. In Silico Analysis

A search was performed in PubMed, selecting original articles that include results evaluating proteins with biomarker potential. Seventy-three articles were found from 2011–2023; from these, the evaluation or determination was performed according to immunoaffinity, proteomics (qualitative/quantitative), and the assessment and validation of the expression of genes associated with HPV 16 integration. Articles in which proteins were analyzed from samples of cervical cytology, paraffin-embedded biopsies, fresh biopsies, cervical cones, cell lines derived from cervical carcinoma, and plasma/serum samples from patients with premalignant and malignant lesions were included, generating a database of 2071 proteins (Supplementary Table S1).

### 2.2. Interaction Network Analysis

The protein analysis began with the search for Uniprot codes (https://www.uniprot.org/, accessed on 1 September 2021) considering the following criteria: current codes and names of the proteins reviewed (records annotated manually with information extracted from the literature and specialized computational analysis). Codes corresponding to isoforms and obsolete codes were eliminated. According to Interaction Network analysis with 0.900 confidence criteria and hiding no interacting nodes, the in silico analysis involved 1434 proteins. The STRING (https://string-db.org/, accessed on 29 January 2023), IntAct (https://www.ebi.ac.uk/intact/, accessed on 29 January 2023), and Cytoscape (https://cytoscape.org/, accessed on 29 January 2023) databases were used for protein–gene interaction analysis. For the analysis of molecular processes, biological processes, and signaling pathways, the following databases were used: GENE ONTOLOGY (https://geneontology.org/, accessed on 4 October 2023), Phanter (https://www.pantherdb.org/, accessed on 4 October 2023), KEEG (https://www.genome.jp/kegg/pathway.html, accessed on 4 October 2023), and Reactome (https://reactome.org/, accessed on 15 January 2024). The original dataset (from review) and TCGA-CESC data analysis for UALCAN proteins, which correlate experimentally with MAGI-1 according to the criteria (median TPM > 0.5), were considered in this database according to the Pearson-CC analysis (Supplementary Table S2).

### 2.3. Cell Lines

From cell lines: spontaneously immortalized keratinocytes from adult human skin (HaCaT), HPV-free squamous cell carcinoma (C-33A), squamous cell carcinoma with 1–2 viral copies of HPV 16 integrated (SiHa), and squamous cell epidermoid carcinoma with >600 viral copies of HPV 16 integrated (CaSki) [29]. The cell lines were cultured in Dulbecco’s modified Eagle medium (DMEM) supplemented with 10% fetal bovine serum (FBS) and penicillin–streptomycin (100 U/mL) in a 5% CO_2_ strain.

### 2.4. Collection of Pap Smears, Cervical Tissue, and Informed Consent

Cytological and histological samples were collected under the Bioethics Committee of the Autonomous University of Guerrero. Informed consent was obtained from all participants (women with a cyto-histological diagnosis of premalignant lesions, ISCC, and HPV 16 infection). All patients were informed of the use of their samples for biological research. Liquid-based cytology samples were obtained from the Cytopathology and Histochemistry Research Laboratory Biobank with the HPV 16 physical status assessment previously performed by [30]. The biopsy was obtained and focused on the lesion site via guided colposcopy, and a certified pathologist made the histological analysis. A total of 14 cervical intraepithelial neoplasia CIN 1 samples and 20 CIN 2–3 samples from the Raymundo Abarca Alarcon hospital were included. In addition, 4 samples with a diagnosis of ISCC were collected at the Cancerology Institute from Guerrero State, which considered all cervical samples with integrated HR-HPV in this study. Cytologies will be selected from 5 total groups of patients: 10 cervical cytologies, no squamous intraepithelial lesions (No-SIL) and HPV negative; 10 cytological samples without SIL and HPV-16-positive; 10 cytologies with low-grade SIL (LSIL) diagnosis; 10 high-grade SIL (HSIL) cytologies; and 10 ISCC, all of them with the integrated version of HPV 16.

### 2.5. Western Blot

Total extracts of HaCaT, C-33A, SiHa, and CaSki cell lines were incubated with RIPA buffer (Tris-HCl pH 7.5 50 mM; EDTA 1 mM; NaCl 100 mM; NP-40 1%); proteins were separated by 8% SDS PAGE and transferred onto 0.22 µm nitrocellulose membranes (NTC). The NTC membranes were blocked at 37 °C for 2 h in PBS 1× Tween (10% milk) and then incubated at 4 °C with anti-MAGI-1(SS-5, Cat. No. sc-100326, Santa Cruz Biotech, Santa Cruz Biotech, Dallas, TX, USA) (dil 1:1000) and mouse anti-IgG1 β-actin (937215, Cat. No. MAB8929, R&D Systems, Minneapolis, MN, USA) (dil 1:1000) overnight. Subsequently, the membranes were incubated with mouse HRP secondary Ab (Cat. No. 115-035-003, Jackson-Inmuno Research, Baltimore, MD, USA) (dil 1:1000). Finally, photographic plates developed the bands using Luminol Reagent (Cat. No. sc-2048, Santa Cruz Biotech).

### 2.6. RT-qPCR

MAGI-1 mRNA expression was analyzed using primers and TaqMan probes specific to this mRNA according to the TaqMan and assay protocol, using the *MAGI-1* TaqMan gene (ID 2023170; Thermo Fisher Scientific, Waltham, MA, USA). RT-qPCR was performed using Applied Biosystems 7900 HT Real-Time Real PCR equipment using 1.0 μL of the mRNA RT product in a reaction volume of 10 μL with 2× Master mix PCR TaqMan Universal and 1 μL of the primer and probe mix according to the TaqMan mRNA assay protocol (Thermo Fisher Scientific). Reactions were incubated at 95 °C for 10 min, followed by 45 cycles of 95 °C for 15 s and 60 degrees for 30 s. Relative quantification of the expression of each mRNA was determined from the critical cycles (threshold cycles), which were defined as the fractional number at which fluorescence exceeds the threshold set by the 2^−∆∆Ct^ method. mRNA expression of MAGI-1 was normalized using the expression of the *GAPDH* TaqMan gene (ID_7743854_1; Thermo Fisher Scientific). Independent duplicates were made for each condition.

### 2.7. Immunocytochemistry and Immunohistochemistry

The streptavidin–biotin–peroxidase immunocytochemical method was used, using the Cytoscan HRP/DAB cell detection system (Cell Marque Corporation, Hot Springs, AR, USA) with the monoclonal antibody MAGI-1 (SS-5 Cat. No. sc-100326, Santa Cruz Biotech), Ki-67 (SP6, Cat. No. 275S Cell Marque Corporation, Rocklin, CA, USA), and E6 (C1P5, Cat. No. MA1-46057 Thermo Fisher Scientific, Rockford, IL, USA). Cells and cervical tissues with embedded paraffin were subjected to antigenic recovery using citrate buffer pH 6.0 (Newcastle Upon Tyne, UK) at 120 °C for 10 min in a Pascal pot (Dako, Carpinteria, CA, USA). Endogenous peroxidase activity was inactivated using 3% hydrogen peroxide for 15 min. Cells and tissues were incubated with primary antibody for 2 h at 1:100 dilution. Then, a secondary antibody coupled with biotin was added for 30 min, followed by incubation with streptavidin peroxidase (HRP) for 30 min. The reaction was revealed using DAB chromogen, and counterstaining was performed with Harris Hematoxylin for 10 s. The HeLa cell line and brain-embedded paraffin sample were considered positive controls, and the same samples were used, and those that omitted primary antibodies were used as negative controls. Densitometric analysis in the cell lines (HaCaT, C-33A, SiHa, and CaSki) was performed on five randomly selected 40× objective fields, and the average absolute intensity was plotted in arbitrary units. The values correspond to the average of three independent biological replicates analyzed using Image-Pro Plus 6.0 software.

### 2.8. Scoring Criteria

The criteria for assessing MAGI-1 expression via immunocytochemistry in the liquid-based cytology were proposed by [31]. The following quantification scale was used: negative (0% of neoplastic cells showing antigen expression); mild positive (antigen expression present in 1–10% of neoplastic cells); moderate positive (antigen expression present in 11–50% of neoplastic cells); and intense positive (antigen expression present in 51% or more of neoplastic cells). To assess Ki-67, E6, and MAGI-1 expression via immunohistochemistry, the criteria proposed by Portari et al. (2013) [32] were used, where 1+ corresponds to lower third of the epithelium, 2+ to the lower two thirds of the epithelium, and 3+ to more than two thirds up to total thickness of the epithelium.

### 2.9. Immunofluorescence

From cell lines at 50–60% confluence, 35 samples of liquid-cytology-based cells fixed with 4% paraformaldehyde for 20 min at room temperature, and 40 paraffin-embedded biopsies, 3 washes were performed with 1× PBS; cells were permeabilized with 0.2% Triton buffer at room temperature for 15 min, blocked with 0.5% PBS-gelatin, then incubated with the primary antibody MAGI-1 (SS-5, Cat. No. sc-100326, Santa Cruz Biotech) (dilution 1:100) overnight at 4 °C. After the incubation time, cells were incubated at room temperature with a FITC-conjugated secondary antibody (Cat. No. 81-651 goat anti-mouse IgG, ZYMED Laboratories, San Francisco, CA, USA). Nuclei were stained with 4′,6-diamidino-2-diamidino-2-phenylindole dihydrochloride (DAPI) (Sigma Chemical Co., St. Louis, MO, USA). Images were obtained using a Leica DMi8 confocal microscope using LAS AF lite Leica software version 4.3. Each image was generated from 12 Z-slices with at least two different fields of the same area via confocal microscopy at 40× and 65×.

### 2.10. TCGA Dataset

The Cancer Genome Atlas Research Network of Cervical Cancer (TCGA-CESC) was used to visualize the clinical data. The cBioportal resource (https://www.cbioportal.org/, accessed on 14 May 2024) accessed data from the cervical squamous cell carcinoma and endocervical adenocarcinoma database (308 patients, 310 samples). Data for the HPV genotype in cervical samples were extracted from a dataset file [33]. The dataset of viral read counts from HPV was extracted from The Genomic Data Commons (GDC) computational platform from NIH (https://gdc.cancer.gov/, accessed on 14 May 2024), accessing the immune landscape of cancer [34], downloading the data for all TCGA samples (viral.tsv file), and selecting only the viral read counts data for the HPV of CESCs. To access the MAGI-1 expression data for cervical cancer, we used the database (https://xena.ucsc.edu/, accessed on 14 May 2024), considering the criteria, namely, TCGA-cervical cancer (313 samples) organized by histological type (phenotypic), and selected the MAGI-1 expression data (genotypic). Also, data on the expression of MAGI-1 were provided by the dataset (https://ualcan.path.uab.edu/, accessed on 14 May 2024) to compare normal and cancer tissue in the CESC-TCGA dataset. The patient IDs from all three databases were manually revised to generate an Excel database with the data concentrate (patient ID, HPV genotype, integration, read counts, and MAGI-1 expression) (Supplementary Table S3).

### 2.11. Statistical Analysis

Relative frequency for the different variables was determined, and Fisher’s exact and Chi-squared tests were used to evaluate the correlation between population variables and protein expression. The statistical tests used to compare the means of the relative expression of MAGI-1 in the SIL and ISCC study groups were one-way ANOVA and Dunette’s multiple comparison test. A *p* ≤ 0.05 was considered significant. GraphPad Prism 9 and Stata v13.0 software were used for the statistical analysis.

## 3. Results

### 3.1. In Silico Analysis of Integrated HPV 16 Associated Proteins in Cervical Cancer 

**Figure 1:**
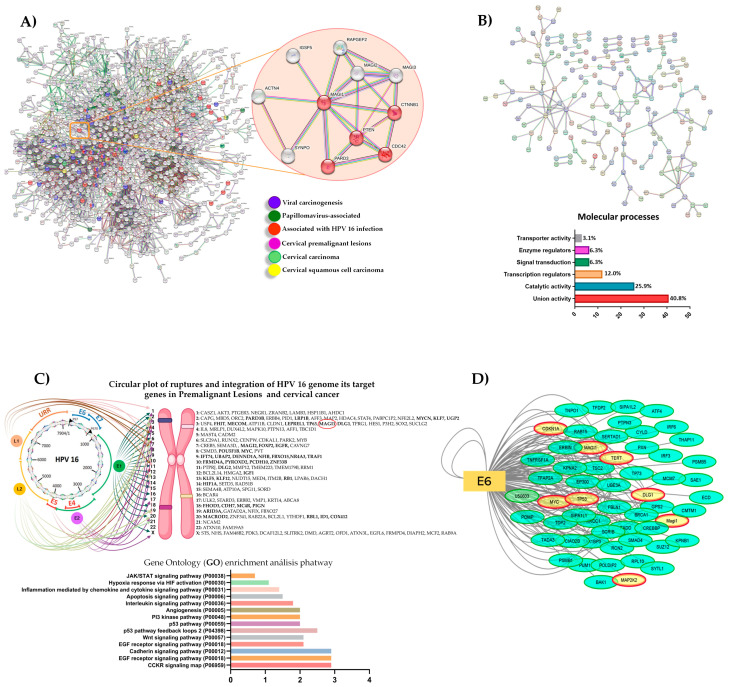
In silico analysis of HPV-16-associated proteins integrated in cervical cancer: (**A**) protein interaction networks associated with HPV 16 infection; (**B**) protein interaction networks associated with VHP integration 16; (**C**) frequent viral genome disruption sites, insertion sites, genes deregulated by integration, and signaling pathways involved in the integration process; (**D**) target proteins with direct physical interaction with the E6 of HPV 16.

Derived from the exhaustive analysis of bibliographic information related to proteins associated with HPV 16 integration in cervical cancer, a database of 2071 proteins was generated (Table S1), from which direct physical interaction networks were designed using the STRING database, eliminating non-interacting nodes, obtaining 1434 proteins considered for the analysis, which correspond to proteins involved in processes such as growth, development, differentiation, and cell cycle (JAK3, RHOA, TP53, CCND1, CDKN1A, CCNE1, and RB); proteins associated with papillomavirus infection other than cervical cancer that participate in cell stability, differentiation, and proliferation (KRT5, KRT14 and FGFR3); proteins associated with HPV 16 infection, highlighting proteins with PDZ domains (MAGI 1, MAGI 2, DLG 2, LAMB3B, and LAMB2); and proteins associated with cervical squamous cell carcinogenesis, which participate in processes such as cell cycle regulation, proliferation, growth, cell migration, DNA replication, and transcription (TOP1, TOP2A, TERT, PTEN, MCM7, RB1, CDKN2A, RPTOR, and SMAD3) (Figure 1A).

Of the 1434 identified proteins, 232 are directly associated with HPV 16 integration. Within the ontology analysis of genes related to the molecular functions in which this group of proteins is involved, it is observed that 40.8% are proteins binding to nucleic acids, enzymes, signaling receptors, and cytoskeleton components. In contrast, 25.9% correspond to proteins with catalytic activity, specifically hydrolase and transferase activity. Finally, 12% are transcriptional regulatory proteins such as transcription factors (Figure 1B). According to next-generation sequencing data reporting on cervical cancer, the main sites of cleavage of the HPV 16 genome are E1, E2, L1, and the frequent sites of insertion into the host cell genome. It can be cleaved in almost any chromosome, most frequently in chromosomes 2, 4, 6, and 9, impacting the expression of proto-oncogenes such as MYC, AKT3, MTOR, KRAS, and PIK3CA, amplifying genes that maintain intercellular junctions, such as MAGI-1, MAGI-2, DLG2, and PARD3B, while inhibiting the expression of tumor suppressor genes such as LEPREEL1, FHIT, LRP1, TP63, and TP53. Ontology analysis of genes affected by integration in cervical cancer showed that the deregulated central pathways are PI3K, P53, WNT, and HIF1, among others (Figure 1C). The analysis from the IntAct and Cytoscape databases revealed that the E6 of HPV 16 has an interaction with 136 host proteins; some of them are CDKN1A, MAGI-1, TERT, MYC, TP53, DLG1, and MAP2K2 (Figure 1D). Of the 136 proteins, 56 are known to interact with the E6 oncoprotein in a direct physical manner, causing its degradation. MAGI-1 is among the few proteins studied in protein expression in premalignant lesions and cancer. It possesses PDZ domains that make it a direct physical target of E6, which is overexpressed due to HPV 16 integration into host genomic DNA. Therefore, MAGI-1 may be a protein related to cervical cancer, HPV 16 genomic integration, and disease progression.

### 3.2. MAGI-1 Expression Profile in Cervical Cancer Cell Lines

**Figure 2:**
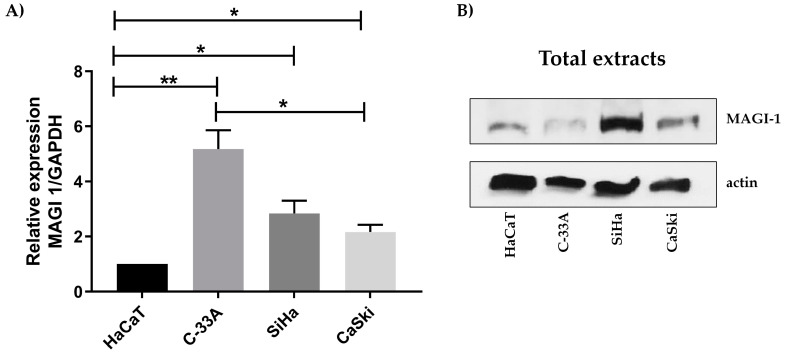
Analysis of MAGI-1 expression in cervical cancer cell lines with integrated HPV 16. (**A**) Increased expression of MAGI-1 mRNA in C-33A with respect to cell lines with HPV 16 in the integrated state where a decrease in MAGI-1 expression is observed. The statistical tests used for comparison of means were one-way ANOVA and Dunnett’s multiple comparison test (*p* < 0.05). (**B**) WB analysis increased MAGI-1 expression in SiHa and CaSki with respect to HaCaT and C-33A. * (*p* ≤0.05), ** (*p* ≤0.01).

To evaluate the changes in expression profiles of MAGI-1 in cervical cancer, the initial analysis in cell lines C-33A, SiHa, and CaSki compared to the non-tumor cell line HaCaT was conducted; higher MAGI-1 expression was observed in the C-33A cell line (*p* ≤ 0.05) concerning HaCaT (*p* ≤ 0.01). In contrast, as to changes in the expression of SiHa and CaSki, no differences were observed (Figure 2A). The expression pattern of MAGI-1 protein in the cell lines showed that in total extracts, a higher expression of MAGI-1 was observed in the SiHa, as well as an increased CaSki cell line in comparison to HaCaT, but no differences between C-33A and HaCaT were observed (Figure 2B).

**Figure 3:**
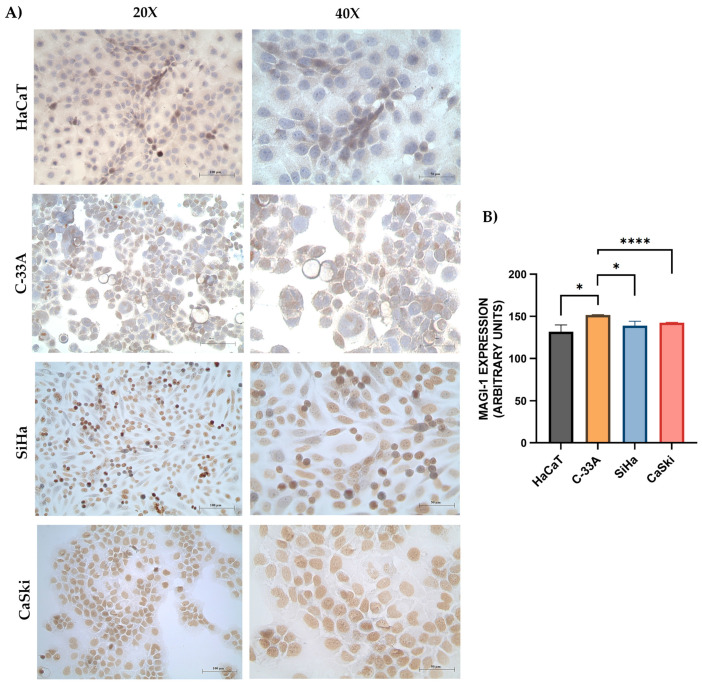
Assessment of MAGI-1 expression and localization in cervical cancer cell lines: (**A**) Lower cytoplasmic expression of MAGI-1 is observed in the HaCaT cell line and only a few positive nuclei. Homogeneous expression of MAGI-1 in the nucleus and a few positive cytoplasm examples is observed in C-33A. A higher number of positive nuclei is observed in SiHa and CaSki. (**B**) According to the staining intensity, a significant difference is observed in C-33A with respect to CaSki (*p* < 0.001). Streptavidin–biotin peroxidase method 20× (100 µm) and 40× (50 µm). One-way ANOVA was used to compare the means of the staining intensity. * (*p* ≤0.05), **** (*p* ≤ 0.0001).

The HeLa cell line was used as a positive control (Figure S1). To evaluate the changes in the subcellular localization of MAGI-1 in cervical cancer cell lines due to the effect of HPV 16 integration, HaCaT, C-33A, SiHa, and CaSki cells were analyzed, and MAGI-1 evaluation was performed via immunocytochemistry; the results showed higher expression in C-33A concerning HaCaT (*p* = 0.0131), but CaSki (*p* ≤ 0.001) and SiHa (*p* ≤ 0.0154) showed lower expression of MAGI-1 in comparison to C-33A. However, interestingly, SiHa and CaSki had a solid nuclear expression pattern, but C-33A had just a few of them (Figure 3).

**Figure 4:**
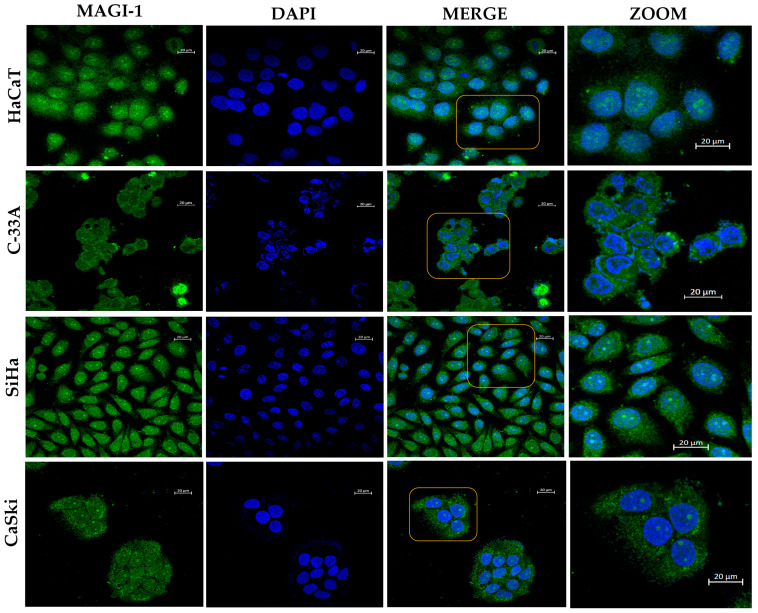
Assessment of MAGI-1 expression and subcellular localization changes in cervical cancer cell lines. The subcellular localization in HaCaT for MAGI-1 is mostly membranal and cytoplasmic; in C-33A, there is cytoplasmic colocalization and that of the perinuclear area; in the case of SiHa and CaSki, MAGI-1 has predominantly nuclear colocalization. Cells were incubated with a FITC-conjugated secondary antibody (Cat. No. 81-651). Nuclei were stained with 4′,6-diamidino-2-diamidino-2-phenylindole dihydrochloride (DAPI). Barr: 20 µm. Yellow boxes are optical zooms.

The HeLa cell line was used as a positive control (Figure S2). Because MAGI-1 had nuclear expression in cell lines with HPV-16 integrated, nuclear colocalization was assessed in HaCaT, C-33A, SiHa, and CaSki cell lines via confocal microscopy. The results showed a cytoplasmic distribution in HaCaT, whereas in C-33A, it is observed at the membrane and cytoplasmic distribution; for cervical cancer cell lines as SiHa, a higher colocalization of MAGI-1 in the nucleus was observed concerning CaSki. Interestingly, in the HPV-negative cervical cancer cell line (C-33A), MAGI-1 expression was mainly observed in the cytoplasm without nuclear colocalization (Figure 4).

### 3.3. Characteristics of the Study Population

Table 1 shows the clinical and demographic characteristics of the population analyzed. The age range of the women in the study was 20 to 86 years, identifying significant differences between the sexual debut (*p* = 0.037), the number of births (*p* = 0.054) with the lesion degree, and cases of ISCC. On the other hand, factors such as patient age, age at menarche, and number of sexual partners showed no significant differences between the groups (*p* > 0.05).

Table 2 shows that the integrated status was the most frequent in patients without HPV-16-positive SIL (80%), LSIL (70%), HSIL (60%), and ISCC, with 100%; while the mixed status represented 22% of the total population analyzed. A total of 5% of the total population analyzed.

### 3.4. Role of HPV Genotype, Integration, Viral Load, and MAGI-1 Expression

**Figure 5:**
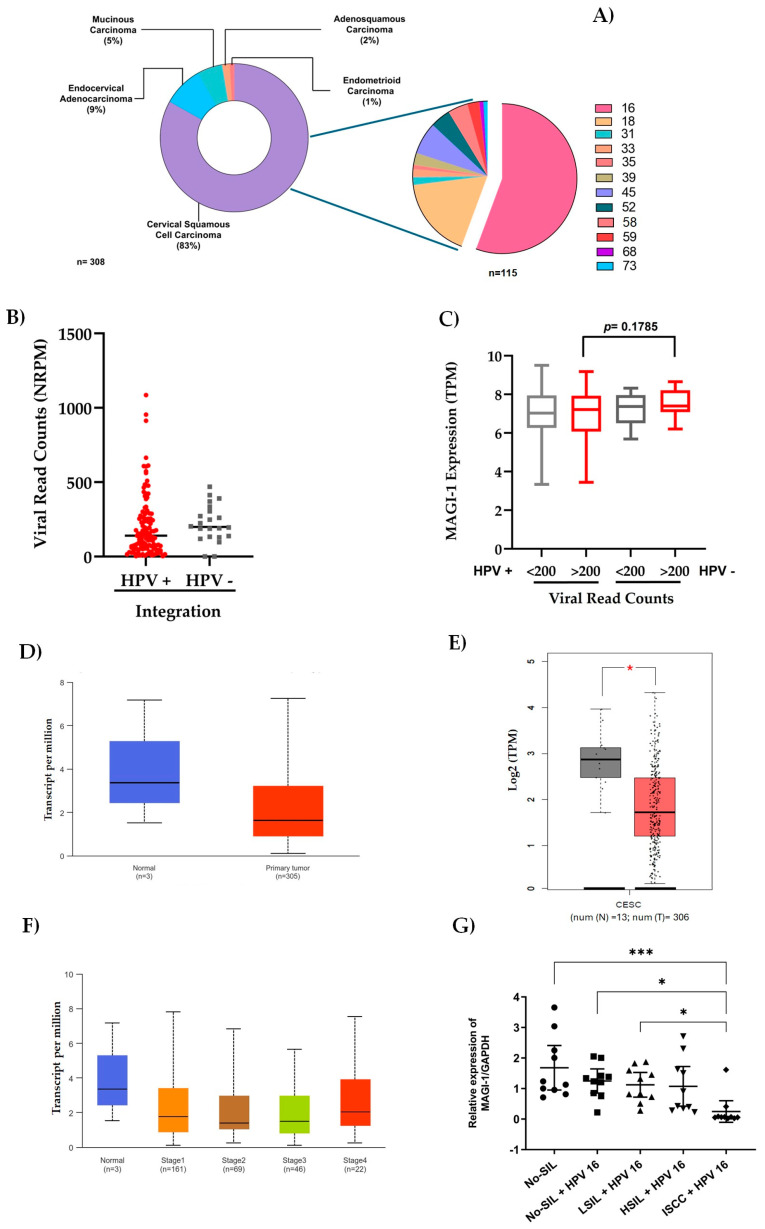
Role of HPV genotype, integration, viral load, and MAGI-1 expression: (**A**) the TCGA-CESC dataset was used to analyze histological types of cervical carcinomas and genotypes more frequent in cervical squamous cell carcinomas with HPV integration; (**B**) viral load profiles (score of normalized reads per million (NRPM)) between HPV (+) and HPV (−) integration; (**C**) correlation between viral load (>200 and <200, NRPM) and *MAGI-1* expression (TPM) in HPV (+) and HPV (−) integration; expression profiles of MAGI-1(TPM) in CESC from TCGA samples analyzed via UALCAN (**D**) and GEPIA (**E**) resources; (**F**) *MAGI-1* expression (TPM) at different clinical stages in CESC; (**G**) experimental relative expression of cytological No-SIL samples, negative for HPV, SIL, and ISCC with HPV 16 integration; the mRNA levels were normalized to the *GAPDH* gene. Data are reported with mean and standard error, and evaluations are made in triplicate. The statistical tests used for comparison of means were one-way ANOVA and Dunnett’s multiple comparison test *p* < 0.05 (*** *p* < 0.001) (* *p* < 0.05).

From the data obtained from TCGA-CESC, which concentrates data from 308 samples organized by histological type, squamous cell carcinoma is the most frequent (83%), with HR-HPV 16 (55.6%) and 18 (17.4%) being the most frequent in this histotype. In contrast, the remaining 27% comprises genotypes 31, 33, 35, 39, 45, 52, 56, 59, 68, and 73 (Figure 5A). Most of the high-risk genotypes are found in their integrated form (85%); however, viral loads greater than 200 NRMP are observed in patients with HR-HPV in its non-integrated form (HPV −, mean of 221.7) relative to those with HR-HPV in its integrated from (HPV +, mean of 202.1), albeit not significantly (Figure 5B). When correlating MAGI-1 expression values concerning viral load in patients with HR-VPH in its integrated (+) and non-integrated (−) versions, no significant differences were observed (Figure 5C). However, when evaluating MAGI-1 expression in cervical cancer tissues concerning tumor tissue in UALCAN (308 samples) and GEPIA (319 samples), the differential expression between normal and tumor tissue is analyzed. The analysis showed an underexpression of MAGI-1 in cancer cases compared to normal tissue (*p* < 0.05) (Figure 5D,E). The analysis of MAGI-1 expression in the different clinical stages of CESC showed a trend towards decreased expression, albeit not significantly (Figure 5F). Relative expression analysis of MAGI-1 in 50 cytological samples from No-SIL and HPV-negative patients and No-SIL, LSIL, HSIL, and ISCC cells patients, HPV 16 integrated, showed a lower expression of MAGI-1 correlating to increasing lesion grade. Significant differences were observed between the No-SIL and HPV-negative group and the HPV-16-positive ISCC cases (*p* < 0.001). In contrast, the No-SIL and LSIL cases with HPV 16 integration showed significant differences (*p* < 0.05) compared to the ISCC group (Figure 5G).

### 3.5. Decreased MAGI-1 Expression Is Associated with the Cervical Lesion Grade

**Figure 6:**
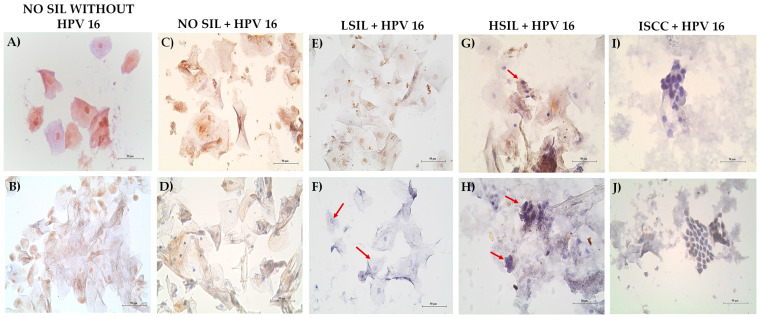
MAGI-1 expression in cervical cytology of No-SIL, HPV-16-positive No-SIL, LSIL, HSIL, and HPV-16-positive cases: (**A**) positive immunocytochemistry for MAGI-1 in the nucleus and cytoplasm; (**B**) more nuclear expression and some positive cytoplasm ((**A**,**B**) are cases with a cytological diagnosis of No-IL, negative for HPV); (**C**,**D**) positive immunocytochemistry for MAGI-1 in cytoplasm and some nuclei in cytologically diagnosed cases of No-IL, positive for integrated HPV 16; (**E**) immunocytochemistry in a case of LSIL positive for MAGI-1 in the nucleus and mild staining in the cytoplasm of intermediate and superficial cells (no cytopathic changes were generated by the virus); (**F**) koilocytes marked with a red arrow, negative for MAGI-1 in cases diagnosed with LSIL with integrated HPV 16; (**G**,**H**) MAGI-1-negative dysplastic cells; some MAGI-1-negative cells of the flat epithelium are also observed; (**I**,**J**) negative immunocytochemistry for MAGI-1 in cases of stage IIB-IIIB non-keratinizing large-cell epidermoid carcinoma with integrated HPV 16. Streptavidin–biotin peroxidase method 40×.

The expression pattern and subcellular distribution of MAGI-1 in patient samples were evaluated through immunocytochemistry and immunohistochemistry. The results showed that 80% of the cases of cells from patients without SIL and negative for HPV infection expressed the protein in >50% of the intermediate and superficial cells in the nucleus and cytoplasm (90% and 20%, respectively), and this group had moderate expression at the cytoplasmic level (Table 3 Figure 6A,B).

Cases with cytological diagnosis of No-SIL and with HPV 16 infection in the integrated physical state showed that 30% were negative for MAGI-1, and 40% of the cases were positive in intermediate and superficial cells at the level of the nucleus and cytoplasm. Finally, 30% of the cases did not show protein expression (Table 3, Figure 6C,D). In the group of patients diagnosed with LSIL, HPV 16 integrated, the results showed that 30% of the cases were negative for the protein, the koilocytes (cells with karyomegaly, perinuclear halo, and binucleation) were negative for MAGI-1, the cells that morphologically did not present any cellular alteration (40%) were positive for the protein, and the rest of the cases (30%) showed a slight expression at the cytoplasm level (Table 3, Figure 6E,F). In HSIL cases, the MAGI-1 protein expression was 40% in at least 1–10% of cells with no apparent morphological alterations, and negative cases were 60%, in which dysplastic cells representative of the lesion were present (equivalent to a high-grade lesion or carcinoma in situ) *(*Table 3, Figure 6G,H). All (100%) of the cases positive for HPV 16 integration of ISCC were negative for MAGI-1 (Table 3, Figure 6I,J).

### 3.6. MAGI-1 Diminishing Expression Is Associated with Cell Proliferation and E6 Overexpression in CIN and ISCC HR-HPV 

**Figure 7:**
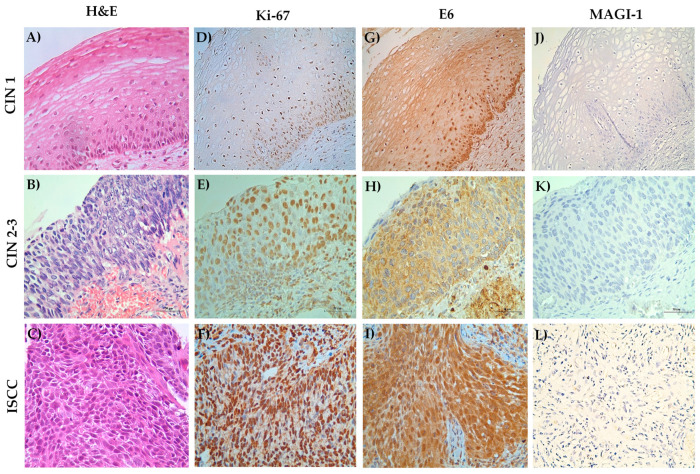
Expression of Ki-67, E6, and MAGI-1 in cases of CIN and ISCC with HR-HPV in the integrated state. (**A**–**C**) H&E staining defined the histological diagnosis. (**D**–**F**) Expression of Ki-67 and (**G**–**I**) E6 throughout the thickness of the epithelium in cases of CIN; expression in tumor nests in the stroma for Ki-67 and E6 in ISCC cases; (**J**–**L**) negative expression for MAGI-1 in cases of CIN and ISCC. Streptavidin–biotin peroxidase method 40×.

For the evaluation of MAGI-1 expression in tissue, a rat brain sample was used (Figure S3). To evaluate if the decreasing of MAGI-1 expression is associated with cell proliferation and E6 overexpression in HR-HPV-integrated CIN and ISCC, the expression of Ki-67, E6, and MAGI-1 was analyzed in 38 cervical biopsies, of which 14 were CIN 1, 20 were CIN 2–3, and 4 were ISCC biopsies. In the CIN 1 group, 79% were positive for Ki-67 in the lower one third of the epithelium, while in the case of E6, 79% were positive in the more than two thirds up to the total thickness of the epithelium. In contrast, MAGI-1 was negative in 79% of these cases, which is related to the integration of the virus, and consequently, the overexpression of the E6 oncoprotein and the cell proliferation evidenced by Ki-67. In the CIN 2–3 group, it was observed that Ki-67 was expressed in more than two thirds (up to the total thickness) of the epithelium, evidencing cell proliferation beyond the histological diagnosis; the expression of E6 in this group was observed in more than two thirds up to the entire thickness) of the epithelium in 90% of cases; and finally, for MAGI-1, it was negative in 85% of cases. In the patients diagnosed with ISCC, Ki-67 and E6 were positive in 100% of cases. In contrast, MAGI-1 was negative in 100% of cases (Table 4, Figure 7).

### 3.7. Changes in the Subcellular Localization of MAGI-1 in Premalignant Lesions and ISCC with HPV 16 Integrated

**Figure 8:**
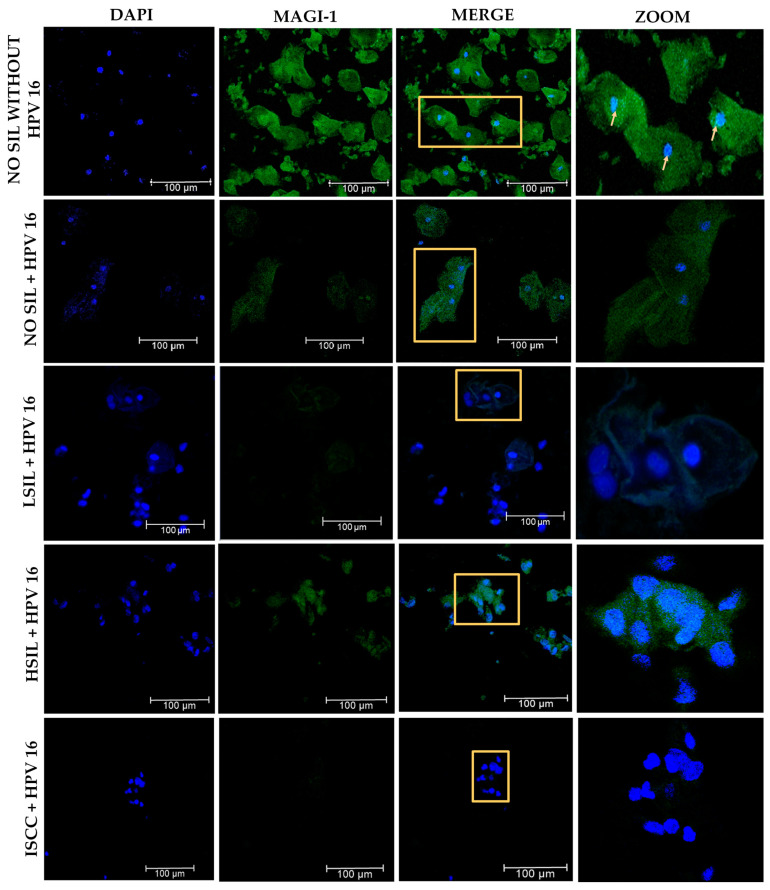
Changes in the subcellular localization of MAGI-1 in cervical cytology of SIL and ISCC with integrated HPV 16. Nuclei were stained with DAPI (blue) to define cell structures. The presence of MAGI-1 was evidenced via a secondary antibody coupled to FITC (green). The optical section shows the co-localization of MAGI-1 in the nucleus. Subcellular localization shifts from cytoplasm to nucleus as the cervical lesion degree increases. Yellow boxes are optical zooms.

To evaluate the changes in the subcellular distribution of MAGI-1, 35 cervical cytology samples were analyzed. In the No-SIL group, negative for HPV, a cytoplasmic and nuclear localization was observed in the superficial epithelial cells; in the No-SIL group of patients, with HPV 16 integrated, a decrease in MAGI-1 expression was related to localization at the nucleus and cytoplasm level in the intermediate and superficial cells. In the LSIL group, specifically in the cells characteristic of this lesion (koilocytes), a slight expression was observed in the nucleus and cytoplasm, being more evident in those cells that do not present an apparent morphological change induced by HPV 16 infection. The HSIL group showed a decreased protein expression; however, in dysplastic cells with karyomegaly, a nuclear and cytoplasmic localization was observed. Finally, in ISCC, there was no protein expression (Figure 8).

**Figure 9:**
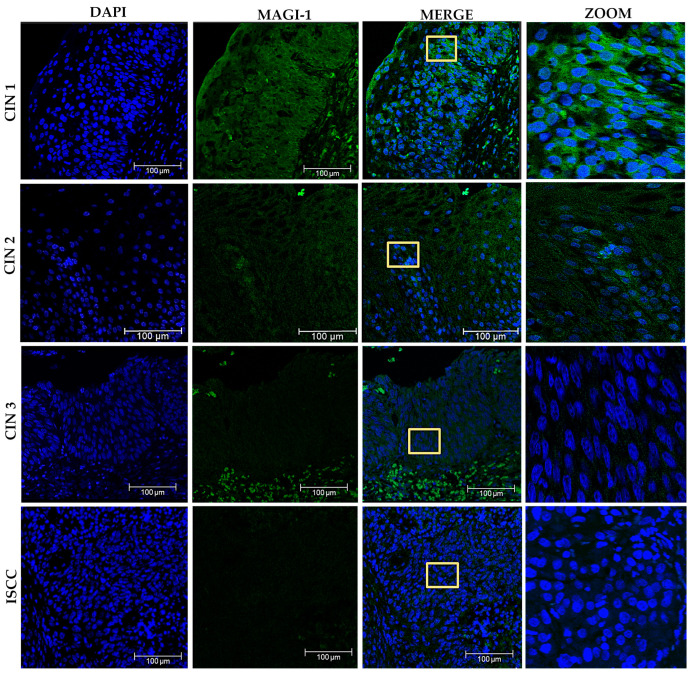
Evaluation of MAGI-1 expression and relocalization in cervical tissues of CIN and HR-HPV-positive ISCC. A decrease in expression is observed as the cervical lesion progresses, with relocalization of MAGI-1 in the nucleus and cytoplasm in CIN 1 and CIN 2, and negative expression of MAG-1 in CIN 3 and ISCC. Nuclei were stained with DAPI (blue) to help define tissue structures; the presence of MAGI-1 was evidenced with a secondary antibody coupled to FITC (green). Optical zoom shows the changes in the subcellular localization of MAGI-1. Yellow boxes are optical zooms.

For the evaluation of MAGI-1 expression in tissue, a rat brain sample was used (Figure S4). Subsequently, 45 cervical tissue sections including cervical intraepithelial neoplasia (CIN) were graded on a scale from 1 to 3, which can also be expressed descriptively as mild, moderate, or severe dysplasia or carcinoma in situ (CIS), and ISCC with HR-HPV were analyzed. In the CIN 1 group, moderated-to-intense expression was observed in the cells of the stratum basal at the membrane level; in the intermediate and superficial cells, the relocalization of MAGI-1 was observed in the nucleus; in the CIN 2 group, there was evidence of decreased expression; and in some intermediate cells, the subcellular localization was observed at the nuclear and cytoplasmic level. In the case of CIN 3 and ISCC, all cases were negative for MAGI-1 expression (Figure 9).

## 4. Discussion

The persistence of the viral infection is a process related to cancer development and progression associated with HPV 16 infection; when viral genome integration occurs, it can cause mutations, the silencing of tumor suppressor genes, and amplification of proto-oncogenes [35]; this, in turn, triggers different molecular processes, such as the overexpression of its E6/E7 oncoproteins and, as a consequence, the degradation of tumor suppressor proteins responsible for regulating processes such as the cell cycle and the proliferation, migration, and invasion of infected cells [36].

Through next-generation sequencing (NGS) studies, it has been observed that there are frequent sites of integration in different types of cancer because in the human genome, there are fragile sites susceptible to DNAase, i.e., transcriptionally active, genomic regions with gene enrichment, and CpG islands, and in open chromatin, this integration occurs through microhomology [37]. Interestingly, it has been seen that the integration of the viral genome generates a loss of function of genes because the integration occurs within the gene or in surrounding regions [38]. However, when the integration occurs in the promoter region, the viral promoters p97 and p670 are integrated upstream of the proto-oncogenes [39]. Recurrently, the amplification of proto-oncogenes such as TP63, MYC TPRG1, IL1RAP, KLF5, and KLF12 has been observed [40].

HPV 16 integration in early lesions is random, but in HSIL and ISCC with viral persistence, viral genome integration occurs in the loci of tumor suppressor genes and proto-oncogenes [41]. Recently, in a sequencing study of long regions (>5 kb) employing a nanopore strategy evaluating HPV 16 integration sites in cervical cancer tumors, four types of integration were described; type A integration integrates a truncated viral genome harboring E6/E7 genes; type B integration is characterized by a truncated viral genome, where E6/E7 genes are not present; type C integration is characterized by a complete integration of the intact viral genome; and type D integration is the final example, in which a combination of types A, B, and C is observed [42]. All these integration possibilities have been analyzed and found to occur mainly in the intergenic, intragenic, and promoter regions of genes encoding PDZ proteins such as MAGI-1/2, DLG2, PARD3B, PTPN13, FRMPD4, and MAST4 [38,39,42].

The integration of HR-HPV into the host genome is a crucial feature of invasive cervical cancer [33,37]. However, there are few studies on the integration of the HR-HPV genome in patient samples; Burk et al. (2017) analyzed 178 core-set tumors, showing that 95% were HPV-positive (HPV 16 and HPV 18, most frequently) and revealing a detectable expression of E6 and E7 oncogene mRNAs, indicating the integration of HPV into the host genome in 83% of samples. Also, Wang X et al. (2022), through the analysis of 214 samples of small-cell cervical carcinoma (SCCC), performed high-throughput HPV-captured sequencing and showed that HPV18/16 infections and integrations were almost dominant in SCCC cases [43].

The TCGA-CESC database is a highly relevant genomic program; however, it concentrates little information on normal tissue, concerning, as it does, little-characterized tumor tissue. The analysis of MAGI-1 expression has been little studied. The use of resources such as cBioportal, GDC, Gepia, GTeX, and UALCAN allowed for the concentration of data to reveal the relationship between MAGI-1 expression and integration, viral load, and clinical stage in 308 tissues with cervical cancer, of which 115 had data on HPV genotype, integration, viral load, and MAGI-1 expression (Figure 5).

High viral load has been reported to be a risk factor for cervical cancer, although the association is not very clear for other HPV types compared to HPV 16 [44,45,46]. Data retrieved from TCGA-CESC (Supplementary Table S3), in which viral load, integration, and MAGI-1 expression were concentrated from 172 samples, did not generate significant results when comparing integration with viral load or integration with viral load and MAGI-1 expression (Figure 5B–C).

In the cell lines, the overexpression of MAGI-1 is observed in C-33A, a cell line that is not infected with HPV; however, in the cell lines SiHa and Caski, an increase in expression is observed concerning HaCaT; these results could indicate that VPH 16 could have an impact on the changes in its expression, probably via the proteins that regulate it, such as STAT 1. STAT 1 is one of the transcription factors that regulate MAGI-1 expression; it has been seen that E6 induces the degradation of this transcription factor through E6AP [47]. Considering that SiHa and CaSki cell lines present HPV 16 integration and that, as a consequence of the integration, the overexpression of E6 is induced, it can be inferred that E6 induces the degradation of the transcription factor that regulates MAGI-1; therefore, an increase in mRNA expression is observed.

In gastric cancer, it has been reported that the *MAGI-1* gene presents somatic mutations in the region coding for the WW domain and PDZ1 [48]; this could be another mechanism that explains the lack of MAGI-1 function. MAGI-1 expression can be regulated through microRNAs such as miR-486-5p in erythroid differentiation [49], and in renal carcinoma, miR-520h has been considered a poor prognosis for patients [50]. So far, there are no reports of MAGI-1 promoter methylation status in cervical cancer. However, the MAGI-1 promoter has been reported to be hypermethylated in anaplastic thyroid cancer [51] and acute lymphoblastic leukemia [52].

At the protein level, the results showed a higher expression of MAGI-1 in C-33A, probably due to the absence of HPV 16 infection, which does not induce its degradation via E6 oncoprotein, despite being a cell line derived from a primary tumor, compared to SiHa and CaSki. However, it should be taken into account that the CaSki cell line presents more integrated viral copies (60–600 viral copies) concerning SiHa, which only has 1–2 viral copies [53], and this could translate to an increase in the overexpression of the E6 of HPV 16 and, consequently, more significant degradation of MAGI-1.

According to the results of immunocytochemistry, concerning the SiHa and CaSki cell lines, where the protein was expressed exclusively in the nucleus, the presence of MAGI-1 was observed in the membrane, cytoplasm, and nucleus in the C-33A cell line. MAGI-1 localization is not limited to being part of adherent junctions and cell–cell or extracellular matrix cell tight junctions; it is also involved in mediating cell migration, signaling, proliferation, survival, vascular integrity, permeability, nitric oxide production, and angiogenesis [54]. Considering its functions and isoforms, which can be expressed in the membrane, cytoplasm, or nucleus, and the interaction it has with the E6 of HPV 16 and its variants, it has been observed that MAGI-1 localization can change depending on the context and interaction with some proteins [17]. Changes in the localization of MAGI-1 in the HeLa cell line by silencing the E6/E7 of HPV 18 through cell fractionation have been analyzed, and most of the protein accumulated in the nucleus and part of the cell membrane [55]. It is essential to mention that the canonical MAGI-1 sequence has two nuclear localization signals in the carboxyl-terminal region [20,56], which explains the nuclear localization of the protein in the cell lines analyzed. 

MAGI-1 interacts with different signaling molecules, such as PTEN, the RhoA-specific GEF/NET1, and catenin, suggesting that MAGI 1 may function as a modulator of multiple signaling pathways [57]. The involvement of MAGI-1 in different molecular mechanisms has been analyzed; it has been observed that the K499 mutation of the PDZ 1 domain of MAGI-1 (K499E) reduces the binding affinity to the PBM motif of the E6 of HPV 18, increasing the expression of ZO-1 and PAR3 and decreasing cell proliferation due to its role in modulating proteins involved in the G1/S transition in the cell cycle [27]. MAGI-1 has also been associated with the induction of apoptosis in cervical cancer, as overexpression of MAGI-1 has been associated with cell rounding, apoptotic body formation, and DNA fragmentation [58].

In kidney epithelial cells under conditions of apoptosis, MAGI-1 is cleaved by caspase 3 at Asp761 within the carboxyl-terminal region, where multiple nuclear signaling sites are located; we observe that this moiety was present in the nucleus, while the amino-terminal region was present in the cytoplasm [59]. It has also been reported that in endothelial cells exposed to endoplasmic reticulum stress, p90 ribosomal S6 kinase (p90RSK) binds to MAGI1, leading to the phosphorylation of MAGI-1 at S741 and the deSUMOylation of MAGI-1 at K931. MAGI-1 deSUMOylation at K931 induces nuclear translocation of MAGI-1-p90RSK and MAGI-1-ATF6 complexes, which causes cell apoptosis [60]. The transfection of MAGI-1 in HepG2 cells inhibits cell migration and invasion through the positive regulation of PTEN expression in hepatocellular carcinoma, which is associated with poor prognosis [61]. The silencing MAGI-1 in the MCF7 cell line increased cell proliferation and reduced the number of cells in apoptosis, indicating that MAGI-1 silencing affects S473 phosphorylation of AKT, a direct target of PI3K, and S9 phosphorylation of GSKβ3, a direct target of AKT [24].

This work shows the first evidence of MAGI-1 expression in liquid-based cytology, cervical tissues, and cells. Also, it changes its subcellular localization in cervical cancer; we observe that in other types of cancer, e.g., the over- and underexpression of the protein in estrogen-receptor-positive (ER+) breast cancer, an overexpression of MAGI-1 was evident concerning a triple negative where there is no expression of MAGI-1; in this sense, the overexpression of the protein has been associated with a better prognosis and a better response to treatment [24]. MAGI-1 expression in colorectal cancer cells is null; upon inducing MAGI-1 overexpression in colorectal cancer cells, a change in epithelial-like morphology has been observed, restoring E-cadherin and β-catenin localization at cell–cell junctions; the formation of actin stress fibers, the formation of focal adhesions, and increased cell adhesion to extracellular matrix proteins has been associated with the suppression of Wnt signaling [23].

The behavior of MAGI-1 protein expression in samples from women with premalignant lesions and invasive carcinoma with integrated HPV 16 has not been evaluated yet. When MAGI-1 was assessed via immunocytochemistry in patients diagnosed with No-SIL without HPV, MAGI-1 was expressed in 80% of cases in the nucleus and cytoplasm of normal cells. In the group of patients with No-SIL, LSIL, and HSIL with active HPV 16 infection in the integrated state, a loss of MAGI-1 expression was observed in 30% and 55.6%, respectively. MAGI-1 is an E6 target protein that binds at lysine 499 (K499) of PDZ 1 [62] and, through E3 protein ubiquitin ligase (SH3RF3), induces its degradation [63].

In ISCC cases with HPV 16 in an integrated state, 100% were found to be negative for MAGI-1; loss of MAGI-1 expression is associated with the increased proliferation, invasiveness, and metastasis of different cancer types [54]. There are no reports of MAGI-1 evaluation in samples from patients with premalignant lesions of the uterine cervix. However, regarding the expression of DLG-1 in histological uterine cervix samples of LSIL with HPV-HR, following a change in the localization of the protein from membranal to cytoplasmic in HSIL, DLG-1 expression was observed throughout the total thickness of the epithelium, DLG-1 localization was preferentially cytoplasmic, and the staining was diffuse and very intense, indicating upregulation of DLG-1 and, ultimately, cases of invasive cervical carcinoma. DLG-1 levels were extremely weak or even absent, especially in the invasively growing epithelial foci [64]. The relative abundance of the E6/E7 oncoproteins is a determinant of the relocalization of cell-polarity-associated PDZ-domain proteins such as DLG-1 [16]. Inducing overexpression of the HPV 18 oncoproteins in HEK293 cells, one observes a mainly cytoplasmic localization of DLG-1, and these changes in localization have been shown to favor binding to GTPase RhoG, thus facilitating the invasive potential of HR-HPV-infected cells [65].

Our results suggest that E6 overexpression and Ki-67-labeled cell proliferation are directly proportional to the loss of MAGI-1 expression, as well as to changes in subcellular localization, as nuclear relocalization was observed in samples with a diagnosis of CIN 1–3 with HR-HPV in the integrated state. Surprisingly, no studies assess the expression and changes in subcellular localization of MAGI-1 due to HPV 16 genome integration and E6 overexpression in cervical cytological and histological samples in premalignant lesions. Therefore, our results contribute to a broader picture in the context of tumor progression, as MAGI-1 is considered a tumor suppressor gene, and both loss of expression and changes in subcellular localization in samples of premalignant lesions with HPV 16 in the integrated state could be a potential biomarker associated with tumor progression.

## 5. Conclusions

The interaction network analysis revealed MAGI-1 as a protein associated with HPV 16 integration, with direct physical interaction with E6, and it is located in the hallmark of the activation of invasion and metastasis. Although MAGI-1 in non-tumor cells is expressed in the membrane and cytoplasm, increased expression and a nuclear localization are observed in cells derived from cancerous tissues. However, in tissues from patients with a molecular diagnosis of HPV 16 in the integrated state, a negative correlation is observed between MAGI-1 expression and progression of SIL, highlighting that in cases of CIN 3 and ISCC, there is a total loss of MAGI-1 expression. MAGI-1 is a protein that could be considered as a protein marker associated with HPV 16 integration into the host-cell genome, especially in premalignant lesions, allowing for the discrimination of cells without SIL but with HPV 16 infection with integrated HPV 16, independently of the viral load.

## Figures and Tables

**Table 1:** Population characteristics concerning cytological diagnosis and HPV 16.

Variable	No-SIL without HPV *n* (%)	No-SIL + HPV 16 *n* (%)	LSIL+ HPV 16 *n* (%)	HSIL + HPV 16 *n* (%)	ISCC + HPV 16 *n* (%)	Total *n* = 50	*p*
Age groups							
20–37	6 (60)	2 (20)	5 (50)	6 (60)	2 (20)	21 (43)	
38–49	2 (20)	4 (40)	2 (20)	2 (20)	3 (30)	13 (24)	0.093
50–86	2 (20)	4 (40)	3 (30)	2 (20)	5 (50)	16 (33)	
* SD							
12–16	1 (10)	5 (50)	2 (20)	5 (50)	5 (50)	18 (36)	
17–18	3 (30)	1 (10)	2 (20)	4 (40)	3 (30)	13 (26)	0.037
19–30	6 (60)	4 (40)	6 (60)	1 (10)	2 (20)	19 (38)	
Menarche							
11–12	3 (30)	6 (60)	5 (50)	3 (30)	2 (20)	19 (38)	
13–14	6 (60)	3 (30)	3 (30)	5 (50)	5 (50)	22 (44)	0.697
15–16	1 (10)	1 (10)	2 (20)	2 (20)	3 (30)	9 (18)	
Number of sex partners							
1	7 (70)	4 (40)	6 (60)	8 (80)	6 (60)	31 (62)	0.987
>2	3 (30)	6 (60)	4 (40)	2 (20)	4 (40)	19 (38)	
Giving birth							
0	5 (50)	4 (40)	2 (20)	3 (30)	0	14 (28)	0.054
>1	5 (50)	6 (60)	8 (80)	7 (70)	10 (100)	36 (72)	

Data are expressed as *n* (%) frequencies of the variables *p* < 0.05 (Chi2). * SD: sexual debut; No-SIL: no squamous intraepithelial lesion; SIL: squamous intraepithelial lesion; LSIL: low-grade squamous intraepithelial lesion; HSIL: high-grade squamous intraepithelial lesion; ISCC: invasive squamous cell carcinoma.

**Table 2:** The physical state of HPV 16 in cervical cytology No-SIL, SIL, and ISCC.

Viral Physical State	No-SIL + HPV 16 *n* = 10	LSIL + HPV 16 *n* = 10	HSIL + HPV 16 *n* = 10	ISCC + HPV 16 *n *= 10	Total *n* = 40	*p*
Episomal	0	0	0	0	0	0.170
Mixed	2 (20)	3 (30)	4 (40)	0	9 (22.5)	
Integrated	8 (80)	7 (70)	6 (60)	10 (100)	31 (77.5)	

Data are expressed as *n* (%) *p* < 0.05 (Chi2): No-SIL: non-squamous intraepithelial lesion; LSIL: low-grade squamous intraepithelial lesion; HSIL: high-grade squamous intraepithelial lesion; ISCC: invasive squamous cell carcinoma.

**Table 3:** Expression of MAGI-1 in cervical cytology No-SIL, SIL, and ISCC.

Assessment Criteria	No SIL/without HPV *n* = 10	No-SIL + HPV 16 *n* = 10	LSIL + HPV 16 *n* = 10	HSIL + HPV 16 *n* = 10	ISCC + HPV 16 *n* = 10	*p*
**MAGI-1**						
**Level of expression**						
**Negative**	0	3 (30)	3 (30)	6 (60)	10 (100)	<0.001
**Mild** (1–10% of positive cells)	0	4 (40)	3 (30)	4 (40)	0	
**Moderate** (11–50% of positive cells)	2 (20)	3 (30)	4 (40)	0	0	
**Intense** (>50% positive cells)	8 (80)	0	0	0	0	
**Subcellular localization**						
* **Negative**	0	3 (30)	3 (30)	6 (56)	10 (100)	
Nucleus	0	0	1 (10)	2 (22)	0	<0.001
Cytoplasm	1 (10)	3 (30)	2 (20)	0	0	
Nucleus–Cytoplasm	9 (90)	4 (40)	4 (40)	2 (22)	0	

No-SIL: non-squamous intraepithelial lesion; SIL: squamous intraepithelial lesions; LSIL: low-grade squamous intraepithelial lesion; HSIL: high-grade squamous intraepithelial lesion; ISCC: invasive squamous cell carcinoma. Data are shown in *n* (%). *p* < 0.05, calculated via Fisher’s exact test. Immunocytochemistry was interpreted using the evaluation criteria of [31]. * Negative is considered as the absence of the expression.

**Table 4:** Expression of Ki-67, E6, and MAGI-1 in CIN and ISSC.

	Histological Diagnosis			
Expression Level	CIN 1 *n* (%)	CIN 2–3 *n* (%)	ISCC *n* (%)	*p*
**Ki-67**				
Negative	0	0	0	
1+	11 (79)	1 (5)	0	
2+	2 (14)	6 (30)	0	**<0.001**
3+	1 (7)	13 (65)	0	
Tumor nests in the stroma	0	0	4 (100)	
**E6**				
Negative	0	0	0	
1+	1 (7)	0	0	
2+	2 (14)	2 (10)	0	**<0.001**
3+	11 (79)	18 (90)	0	
Tumor nests in the stroma	0	0	4 (100)	
**MAGI-1**				
Negative	11 (79)	17 (85)	4 (100)	
1+	2 (14)	2 (10)	0	
2+	1 (7)	1 (5)	0	0.089
3+	0	0	0	
Tumor nests in the stroma	0	0	0	

Diagnostic: CIN 1 (cervical intraepithelial neoplasia, grade 1); CIN 2–3 (cervical intraepithelial neoplasia, grades 2–3). Epithelial distribution based on the evaluation of [32]: 1+ (lower one third of the epithelium); 2+ (lower two thirds of the epithelium); 3+ (more than two thirds up to total thickness of the epithelium). Data are shown in n (%) calculated via Fisher’s exact test.

## Data Availability

The data presented in this study are available in this article (and Supplementary Materials).

## Supplementary Materials

The following supporting information can be downloaded at https://www.mdpi.com/article/10.3390/cancers16122225/s1: Table S1: Bibliographic database of proteins associated with infection and integration of VHP 16; Table S2: Original dataset and TCGA-CESC data analysis in UALCAN proteins; Table S3: TCGA-CESC: HPV genotype, integration, viral load, and MAGI-1 expression; Figure S1: Evaluation of MAGI-1 expression in the HeLa cell line as a positive control of immunocytochemistry; Figure S2: Evaluation of MAGI-1 by IF in HeLa cell line as a positive control of immunofluorescence; Figure S3: Evaluation of MAGI-1 expression in rat brain tissue as positive control of immunohistochemistry; Figure S4: Evaluation of MAGI-1 by IF in HeLa rat brain tissue as positive control of immunohistofluorescence.
