# Supplementary material for: Bioinformatics Analysis of Human Papillomavirus 16 Integration in Cervical Cancer: Changes in MAGI-1 Expression in Premalignant Lesions and Invasive Carcinoma
Source: Cancers (Basel). 2024 Jun 14;16(12):2225. doi: 10.3390/cancers16122225 (PMC11202195; doi:10.3390/cancers16122225)
Supplement: Supplementary file 1 [file cancers-16-02225-s001.zip › cancers-2963458-supplementary/cancers-2963458-supplementary/cancers-2963458-Table S1 and Figures S1-S4.pdf]

Table S1: Bibliographic database of proteins associated with infection and integration of VHP 16.

STUDIES OF IHQ PROTEIN ASSESSMENT IN SIL AND CERVICAL CANCER			
**BIBLIOGRAPHIES**	**STUDY POPULATION**	**PROTEIN (UNIPROT CODE)**	**FINDINGS**
Progression of cervical low grade squamous intraepithelial lesions: in search of prognostic biomarkers<br><br>Quint *et al.*, 2013	584 consecutive patients with biopsy proven LSIL and 2-year follow-up were included in the age analysis.	p53 (P04637)<br>pRb (P06400)<br>p16 (P42771)<br>Ki-67 (P46013)	Results: The odds of LSIL persistence and progression were significantly higher in women 30-39, 40-49 and 50+ years old, as compared to women 20-29 years old (OR 1.89, 2.52 and 2.39, respectively). The odds of persistence and progression were higher in women infected with HPV16, 18, 33 and 52 (OR 3.5, 3.1, 3.5 and 2.9, respectively). There were no significant differences in expression of immunomarkers (p16, p53, pRB and Ki-67) between the lesions that regressed versus the lesions that persisted or progressed.
High-risk human papillomavirus load and biomarkers in cervical intraepithelial neoplasia and cancer<br><br>*Chang et al., 2014*	Cervical tissue biopsy in 143 women:<br><br>77 No-SIL without HPV<br>33 CIN I<br>6 CIN II<br>22 CIN III<br>5 CESC	p16INK4a (P42771)<br>Cyclin D1 (P24385)<br>p53 (P04637)<br>COX-2 (P35354)<br>Ki-67 (P46013)<br>GLUT1 (P11166)<br>hPygopus2 (Q9BRQ0)<br>Beta-catenin (P35222)	The relative risk (odds ratio) of p16(INK4A) and GLUT1 overexpression increased gradually according to the histological severity of cervical disease. The p16(INK4A) showed statistically significant odds ratios in CIN II, CIN III, and cancer; GLUT1, in CIN II and CIN III; hPygopus2, in CIN III; and beta-catenin, in CIN III and cancer. Conclusively, HPV load, p16(INK4A), and GLUT1 can be instrumental in predicting the severity of HPV-related cervical disease. The beta-catenin/hPygopus2 signaling may be involved in proceeding to CIN III.

Effect of human papillomavirus on cell cycle-related proteins p16, Ki-67, Cyclin D1, p53, and ProEx C in precursor lesions of cervical carcinoma: a tissue microarray study<br><br>*Conesa-Zamora et al., 2009*	Using tissue microarrays, and HPV genotypes were identified in 144 cervical tissue specimens encompassing normal or benign epithelial lesions, low- and high-grade squamous intraepithelial lesions (LSIL and HSIL, respectively), and CC.	p16 (P42771)<br>Ki-67 (P46013)<br>Ciclina D1 (P24385)<br>p53 (P04637)<br>ProEx C (P49736)<br>(P11388)	Expression of p16, Ki-67, and ProEx C was most associated with the severity of dysplasia. Positive expression of p16, Ki-67, and ProEx C and negative expression of p53 seem to be related to HPV-16 infection. AIM cases show an immunohistochemical pattern more similar to LSIL than to HSIL. Immunohistochemical assessment of cell cycle proteins may help to distinguish normal and benign conditions of the cervix from precursor lesions of CC.
Tissue transglutaminase 2 as a biomarker of cervical intraepithelial neoplasia (CIN) and its relationship to p16INK4A and nuclear factor kappaB expression<br><br>*Gupta et al., 2010*	Twenty cases each with normal cervical histology, CIN1, CIN2, CIN3, and invasive SCC were analyzed	transglutaminasa tisular 2 TG2 (P21980)<br>NF-kappa-B (Q00653)<br>p16 (P42771)	Cytoplasmic as well as nuclear TG2 expression was observed in the epithelial cells. As compared to normal controls, CIN1 showed markedly increased cytoplasmic TG2 expression (p = 0.006). In CIN2/3, additional nuclear TG2 expression was seen (p = 0.009 and 0.031, respectively). Marked extracellular stromal upregulation of TG2 was noted in CIN3/SCC versus normal controls (p = 0.054; p = 0.003). There was no relationship of TG2 with either p16 of NF-kappaB expression. Combining TG2 immunoreactivity with p16 increased the immunolabeling of dysplasia from 35% to 100% in CIN1, 45% to 60% in CIN2, and 60% to 85% in CIN3
Expression of fibroblast growth factor receptor 2 IIIc in human uterine	29 cervical cancer biopsies: patients were	FGFR2 IIIc (P21802)	In CINs 1 and 2, FGFR2 IIIc was found to be localized at the basal to lower two-thirds of the squamous epithelium, whereas it was localized in most of the

cervical intraepithelial neoplasia and cervical cancer<br><br>Kawase *et al.*, 2010	classified into keratinising type (8 patients) and non-keratinising type (21 patients) of SCC.<br>15 CIN1<br>15 CIN2<br>15 CIN3		squamous epithelium, except for the superficial layer in CIN 3. In situ hybridization (ISH) analysis showed that the expression patterns of FGFR2 IIIc mRNA are similar to those of FGFR2 IIIc protein in CINs. The FGFR2 IIIc protein was detected in all invasive cervical cancer patients (29 cases) and its mRNA was found to be strongly expressed in the invasive front of cancer cell nests. FGFR2 IIIc cDNA was stably transfected into CaSki cells, which are derived from a cervical SCC.
The impact of epithelial biomarkers, local immune response and human papillomavirus genotype in the regression of cervical intraepithelial neoplasia grades 2-3<br><br>*Ovestad et al., 2011*	55 cases of CIN2-3 in cervical biopsies with subsequent cervical cones were studied retrospectively to assess how epithelial biomarkers	CD4 (P01730)<br>CD8 (P01732)<br>CD25 (P01589)<br>CD138 (P18827)<br>p63 (Q9H3D4)<br>Histone H3 (P68431)<br>CK-13 (P13646)<br>CK-14 (P02533)<br>p16 (P42771)<br>pRB (P06400)<br>p53 (P04637)<br>Ki-67 (P46013)	18% of CIN2-3s regressed (median biopsy-cone interval of 12.0 weeks, range 5.0-34.1 weeks). CIN2-3s that regressed had higher epithelial pRb and p53, lower stromal CD25(+) and CD138(+), and higher CD8 cells than persistent lesions. They also had higher ratios of CD4(+)/CD25(+) and CD8(+)/CD25 in stroma and epithelium. HPV16 correlated with low pRb and low CD8(+). With multivariate analysis a combined high ratio of CD8(+)/CD25(+) in the stroma, high epithelial pRb and p53 expression had independent value to predict the regression.
Expresión nuclear de Rac1 en lesiones premalignas de cuello uterino y células de cáncer de cuello uterino	102 cervical paraffin-embedded biopsies: 20 without Squamous Intraepithelial Lesions (SIL), 51 Low- grade	Rac 1 (P63000)<br>RhoA (P61586)<br>Cdc42 (Q9NRR8)<br>TIAM1 (Q13009)<br>beta-Pix (Q14155)	Immunoreacivity for Rac1, RhoA, Tiam1 and beta-Pix was stronger in L-SIL and H-SIL, compared to samples without SIL, and it was significantly associated with the histological diagnosis. Nuclear expression of Rac1 was observed in 52.9% L-SIL and 48.4% H-SIL, but not in samples without SIL. Rac1

Mendoza-Catalán et al., 2012	SIL, and 31 High-grade SIL		was found in the nucleus of C33A and SiHa cells but not in HaCat cells. Chemical inhibition of Rac1 resulted in reduced cell proliferation in HaCat, C33A and SiHa cells.
Dynamin 2 expression as a biomarker in grading of cervical intraepithelial neoplasia<br><br>*Lee et al., 2012*	All the patients with reactive changes (n=7) or normal (n=4) did not show dynamin 2 expression. There were 33, 14, and 12 cases with CIN I, II, and III, respectively	Dynamin 2 (P50570)	Negative expression of dynamin 2 was more sensitive for the detection of CIN II/III than high expression (2+) of Ki-67 (96.2% vs. 73.1%, P=0.041). Among patients in whom HPV infection was detected, the degrees of dynamin 2 expression were not associated with the type of HPV infection (low-risk vs. high-risk). Overall, there was a negative correlation between the expression patterns of Ki-67 and dynamin 2.
Risk of progression of early cervical lesions is associated with integration and persistence of HPV-16 and expression of E6, Ki-67, and telomerase<br><br>*Vega- Peña et al., 2013*	A total of 75 cytological specimens in liquid base (Liqui-PREP) were analyzed: 25 specimens were with no signs of SIL (NSIL) and without HPV; 25 NSIL with HPV-16, and 25 with both LSIL and HPV-16.	Ki-67 (P46013)<br>E6 (P03126)<br>hTERT (O14746)	Of the total group, 58.6% had LSIL associated with persistence and of these 59.3% was associated with integrated state of HPV as intense expression of E6, Ki-67 (P = 0.013, P = 0.055) has except for the expression of telomerase present a non-significant association (P<0.341).
Peroxiredoxin 3 is a novel marker for cell proliferation in cervical cancer<br>*HU et al., 2013*	68 patients with invasive squamous cervical cancer:<br><br>56 positive for HPV 16	Peroxirredoxin 3 (P30048)	All samples were positive for high-risk HPV, among which fifty-six samples were positive for HPV16, seven for HPV18 and five for HPV33. The expression of HPV16 E6/E7 was significantly higher in cancer areas compared to the adjacent normal epithelial tissues. The positive cells for Prx3 and Ki67 were significantly higher in cancer cells compared to

	7 positive for HPV 18<br>5 positive for HPV 33		normal epitheliums and the staining pattern of Prx3 was consistent with that of Ki67 (Pearson's correlation coefficient was 0.801, P= 0.000). The upregulation of Prx3 might be a protective response to oxidative stress in the cancer microenvironment.
The integration of HR-HPV increases the expression of cyclins A and E in cytologies with and without low-grade lesions<br><br>Zubillaga-Guerrero et al., 2013	115 cytological specimens in liquid base (liquid-PREP™) were analyzed. 25 specimens were with no signs of SIL (NSIL) and without HPV; 30 with NSIL with low-risk HPV (LR-HPV); 30 with NSIL with HR-HPV; and 30 with both LSIL and HR-HPV.	Ciclina A (P78396)<br>Ciclina E (P24864)	In the cytologies NSIL with LR-HPV, the expression of cyclin-A and cyclin-E was found respectively in 23.3% and 33.3% of the specimens. Among the specimens of NSIL with HR-HPV, 33.3% expressed cyclin-A and 40% cyclin-E, while 100% of the LSILs expressed the 2 cyclins. On the other hand, 100% of the samples NSIL with LR-HPV presented an episomal pattern.
Cervical squamocolumnar junction-specific markers define distinct, clinically relevant subsets of low-grade squamous intraepithelial lesions<br><br>Herfs *et al*., 2013	214 patients were classified by 2 experienced pathologists (panel) as LSIL or HSIL using published criteria. SILs were scored SCJ and SCJ using SCJ-specific antibodies (keratin7,	p16 (P42771)<br>Ki-67 (P46013)<br>**Krt 7 (P08729)**<br>**AGR 2 (O95994)**<br>**GDA (Q9Y2T3)**<br>**MMP7 (P09237)**	The original diagnostician agreed with the panel diagnosis of HSIL and SCJ LSIL in all cases (100%). However, for SCJ LSIL, panelists disagreed with each other by 15% and with the original diagnostician by 46.2%. Comparing SCJ and SCJ LSILs, 60.2% and 94.9% were p16 positive, 23% and 74.4% showed strong (full-thickness) p16 staining, and 0/54 (0%) and 8/33 (24.2%) with follow-up had an HSIL outcome, respectively. Some SCJ LSILs are more

	AGR2, MMP7, and GDA).		likely to both generate diagnostic disagreement and be associated with HSIL. Conversely, SCJ LSILs generate little observer disagreement and, when followed, have a very low risk of HSIL outcome
Altered expression of ezrin, E-Cadherin and β-Catenin in cervical neoplasia<br><br>*Auvinen et al., 2013*	45 biopsies all with HR-HPV of which:<br>26 CIN 1<br>12 CIN 2<br>3 CIN 3<br>4 adenocarcinoma *in situ* (AIS)	p16 (P42771)<br>Ezrina (P15311)<br>β-catenina (Q02248)<br>E-cadherina (P12830)<br>Six-1 (Q15475)	Based on our previous work on ERM proteins we sought out to study the expression of ezrin in cervical premalignant lesions. We also studied the expression of E-cadherin and β-catenin, which play an important role in epithelial cell adhesion. We observed intensifying expression of ezrin along with progressing grade of neoplasia. Ezrin staining was found to colocalize with p16 staining in high-risk HPV associated lesions. Expression of E-cadherin and β-catenin was found to be altered along with the severity of the lesion, similar to ezrin.
High expression of astrocyte elevated gene-1 (AEG-1) is associated with progression of cervical intraepithelial neoplasia and unfavorable prognosis in cervical cancer<br><br>Huang *et al., 2013*	Cervical biopsies:<br>18 CIN 1<br>17 CIN 2<br>15 CIN 3<br>74 SCC<br>16 adenocarcinoma	AEG-1 (Q86UE4)	The expression level of AEG-1 was increased from CIN I to CIN III. High expression of AEG-1 could be observed in 61.1% (55/90) of cervical cancer. Moreover, high expression of AEG-1 correlated with tumor size and lymph node metastasis (all $P < 0.05$). More importantly, high expression of AEG-1 was closely associated with cervical cancer patient shortened survival time as evidenced by univariate and multivariate analysis ($P < 0.05$).
Expression of E6, p53 and p21 proteins and physical state of HPV16 in cervical	101 liquid-based cytological samples were analyzed.	E6 (P03126)<br>p53 (P04637)<br>p21 (P38936)	The expression of E6, p53 and p21 proteins was evaluated by immunocytochemistry. The expression of HPV16 E6 protein was significantly higher in LSIL that in Non-SIL samples (p=0.006). We found a

cytologies with and without low grade lesions<br><br>Jimenez-Tagle *et al., 2014*	50 without squamous intraepithelial lesions (Non-IL) and 51 samples of low grade squamous intraepithelial lesions (LSIL), both with HPV16 infection.		significant correlation between E6 expression and the physical state of HPV16 in Non-SIL (p=0.049).
Expression of PBK/TOPK in cervical cancer and cervical intraepithelial neoplasia<br><br>Luo *et al*., 2014	28 cases of low-grade cervical intraepithelial neoplasia (CINI), 62 cases of high-grade intraepithelial neoplasia and 80 cases of cervical cancer by immunohistochemistry (IHC).	PBK/TOPK (Q96KB5)	PBK/TOPK expression was significantly greater in cervical cancer than that in high-grade intraepithelial neoplasia and CINI ($P < 0.05$). Meanwhile, PBK/TOPK expression in high-grade intraepithelial neoplasia was significantly higher compared with that in CINI ($P < 0.05$). In addition, PBK/TOPK expression in cervical cancer significantly correlated with histological type, differentiation, lymph node metastasis, vaginal and cervical invasion, TNM stage and tumor size ($P < 0.05$).
Immunohistochemical LRIG3 expression in cervical intraepithelial neoplasia and invasive squamous cell cervical cancer: association with expression of tumor markers, hormones, high-risk HPV-infection, smoking and patient outcome	Cervical biopsies from 129 patients with invasive squamous cell carcinoma and 170 biopsies showing low grade and high grade CIN, or normal epithelium were stained for LRIG3 and 17 additional tumor markers	LRIG3 (Q6UXM1)<br>pRB (P28749)<br>CK10 (P13645)<br>C-myc (P01106)<br>E-cadherina (P12830)<br>p53 (P04637)<br>p16 (P42771)	In CIN, high expression of the tumor suppressors retinoblastoma protein, p53, and p16, and E-cadherin (cell-cell interaction), or low expression of CK10, correlated to LRIG3 expression. In addition, progestogenic contraceptive use correlated to high expression of LRIG3. In invasive cancer there was a correlation between expression of the major tumor promoter c-myc and high LRIG3 expression. High LRIG3 expression correlated significantly to presence of high-risk HPV infection in patients with normal epithelium and CIN. There was no correlation

Lindström *et al*., 2014			between LRIG3 expression and 10-year survival in patients with invasive cell cervical cancer. LRIG3 expression is associated with a number of molecular events in CIN.
Fascin expression in cervical normal squamous epithelium, cervical intraepithelial neoplasia, and superficially invasive (stage IA1) squamous carcinoma of the cervix<br><br>Koay *et al., 2014*	Cervical biopsies showing normal squamous epithelium (n=10), CIN 1 (n=10), CIN 2-3 without invasion (n=11), and CIN 2-3 adjacent to SCC (n=40)	Fascin (Q16658)	Fascin expression in normal squamous epithelium was restricted to basal and parabasal cells, whereas there was increased staining in immature squamous metaplasia and in most CIN lesions. Full thickness staining was more frequent in high grade CIN adjacent to invasion than in CIN 2-3 alone. Eighteen SCCs (67%) were fascin positive and seven cases showed accentuated staining at the tumour-stromal interface (invasive front). There was no consistent relationship between fascin expression in CIN lesions and in corresponding carcinomas
Predictive value of the combined p16 and Ki-67 immunocytochemistry in low-grade squamous intraepithelial lesions<br><br>Ziemke *et al.*, 2014	Analyzed the p16(INK4a) and Ki-67 immunocytochemistry (CINtec® PLUS, dual stain) of 260 patients with LSIL. Cytology and dual-stain results were correlated with histology at the time of treatment or with cytological follow-up.	p16 (P42771)<br>Ki-67 (P46013)	After an average duration of 24.9 months (1-58) and a histology rate of 36.2% [cervical intraepithelial neoplasia, grade 2 or higher (CIN2+) as positive], the statistical evaluation for cytology and dual stain resulted in a sensitivity of 98.3 and 90.0%, respectively, a specificity of 74.5% for dual stain, a positive predictive value (PPV) of 22.8 and 51.4%, and a negative predictive value (NPV) of 96.1% for dual stain.
Clinical significance of OCT4 and SOX2 protein	305 normal cervical epithelium samples, 289	OCT4 (Q01860)<br>SOX2 (P48431)	OCT4 and SOX2 expression was higher in cervical cancer than normal cervix (both $p < 0.001$). OCT4

expression in cervical cancer<br><br>Kim *et al*., 2015	cervical intraepithelial neoplasia samples, and 161 cervical cancer cases and compared the data with clinicopathologic factors		overexpression was associated with lymphovascular space invasion (p = 0.045), whereas loss of SOX2 expression was correlated with large tumor size (p = 0.015). Notably, OCT4 and SOX2 were significantly co-expressed in premalignant cervical lesions, but not in malignant cervical tumor. OCT4 overexpression showed worse 5-year disease-free and overall survival rates (p = 0.012 and p = 0.021, respectively) when compared to the low-expression group, while SOX2 expression showed favorable overall survival (p = 0.025)
Enhanced expression of PD L1 in cervical intraepithelial neoplasia and cervical cancers<br><br>Mezache *et al*., 2015	Cervical tissues of which are:<br>55 NO-SIL<br>21 CIN 1/2<br>70 SCCE	PD L1 (Q9NZQ7)	PDL1 protein was not evident by immunohistochemistry in histologically normal cervical epithelia (0/55) even when adjacent to CIN or cancer. PD L1 expression was much increased in CINs (20/21=95%) and cervical squamous cell cancer (56/70=80%) and localized to the dysplastic/neoplastic squamous cells and mononuclear cells, respectively. There was also a significant increase (each P<0.001) in PD L1 detection in mononuclear cells when comparing cervical squamous cell cancers to endometrial (22/115=19%) and ovarian adenocarcinomas (5/40=13%).
Immunohistochemical Expression and Prognostic Significance of CD97 and its Ligand DAF in Human Cervical Squamous Cell Carcinoma	97 patients with CSCC and 53 patients with cervical intraepithelial neoplasia, a precursor lesion of CSCC	CD97 (P48960)<br>DAF (P08174)	CD97 and DAF were absent or only weakly expressed in the normal epithelium of the cervix but were present in 83.5% (81/97) and 90.7% (88/97) of CSCC samples, respectively. Overexpression of CD97 was significantly associated with a high International Federation of Gynecology and

He et al., 2015			Obstetrics stage (P=0.010) and lymph node metastasis (P=0.026). The majority of CSCCs, irrespective of staging/grading classification, displayed strong DAF immunostaining. Kaplan-Meier survival analysis revealed that overexpression of CD97 was associated with a worse prognosis. Multivariate analyses showed that the International Federation of Gynecology and Obstetrics stage (P=0.000), lymph node metastasis (P=0.004), and CD97 expression (P=0.040) were independent risk factors for overall survival.
Molecular transitions from papillomavirus infection to cervical precancer and cancer: Role of stromal estrogen receptor signaling<br><br>*den Boon et al., 2015*	analyzed 128 frozen cervical samples spanning normalcy, increasingly severe cervical intraepithelial neoplasia (CIN1- CIN3), and cervical cancer (CxCa)	AR (P10275)<br>CDKN2A (P42771)<br>CHEK1 (O14757)<br>CXCL14 (O95715)<br>CXCR2 (P25025)<br>ESR1 (P03372)<br>FN1 (P02751)<br>GREB1 (Q9C091)<br>IL-8 (P10145)<br>MTHFD1L (Q6UB35)<br>MUC4 (Q99102)<br>PGR (P06401)<br>SOD2 (P04179)<br>VEGFA (P15692)<br>SYCP2 (Q9BX26)<br>UHRF1 (Q96T88)	Significantly, despite clinical, epidemiological, and animal model results linking estrogen and estrogen receptor alpha (ERα) to CxCa, ERα expression declined >15-fold from normalcy to cancer, showing the strongest inverse correlation of any gene with the increasing expression of p16, a marker for HPV-linked cancers. This drop in ERα in CIN and tumor cells was confirmed at the protein level. However, ERα expression in stromal cells continued throughout CxCa development. Our further studies localized stromal ERα to FSP1+, CD34+, SMA- precursor fibrocytes adjacent to normal and precancerous CIN epithelium, and FSP1-, CD34-, SMA+ activated fibroblasts in CxCas. Moreover, rank correlations with ERα mRNA identified IL-8, CXCL12, CXCL14, their receptors, and other

		MCM4 (P33991)<br>MCM5 (P33992)<br>RNASEH2A (O75792)<br>NUSAP1 (Q9BXS6)<br>DTL (Q9NZJ0)<br>STIL (Q15468)<br>C16orf75 (Q96E14)<br>PHF19 (Q5T6S3)<br>RAD51 (Q06609)<br>MCM6 (Q14566)<br>APOBEC3B (Q9UH17)<br>RFC5 (P40937)<br>MCM2 (P49736)<br>KIF2C (Q99661)<br>TCF19 (Q9Y242)<br>TK1 (P04183)<br>UBE2C (O00762)<br>PRIM1 (P49642)<br>POLE2 (P56282)<br>RRM2 (P31350)<br>CGNL1 (Q0VF96)<br>EPB41L3 (Q9Y2J2)<br>PLAGL1 (Q9UM63)<br>ZNF439 (Q8NDP4)<br>EDN3 (P14138)<br>CTTNBP2 (Q8WZ74)<br>ZNF135 (P52742)<br>CCND1 (P24385)<br>ZNF419 (Q96HQ0)	angiogenesis and immune cell infiltration and inflammatory factors as candidates for ERα-induced stroma-tumor signaling pathways

		SOSTDC1 (Q6X4U4)<br>ZNF844 (Q08AG5)<br>KIAA0232 (Q92628)<br>SLC27A6 (Q9Y2P4)<br>MAOB (P27338)<br>FAM13C (Q8NE31)<br>GREB1 (Q4ZG55)<br>PHYHIP (Q92561)<br>GSTA4 (O15217)	
Circulating soluble neuropilin-1 in patients with early cervical cancer and cervical intraepithelial neoplasia can be used as a valuable diagnostic biomarker<br><br>*Yang et al., 2015*	NRP-1 was measured in 64 preoperative patients and 20 controls. NRP-1 protein in cervical tissue was detected in 56 patients and 20 controls.	NRP-1 (O14786)	Both sNRP-1 and NRP-1 proteins were correlated with stage. sNRP-1 presented a high diagnostic ability of cervical cancer and CIN, with a sensitivity of 70.97% and a specificity of 73.68%.
A study of the expression and localization of toll-like receptors 2 and 9 in different grades of cervical intraepithelial neoplasia and squamous cell carcinoma<br><br>Ghosh *et al*., 2015	This single institution study includes individuals with normal, precancerous lesions, cervical intraepithelial neoplastic (CIN) and invasive squamous cell carcinoma (SCC) of the cervix	TLR 2 (O60603)<br>TLR 9 (Q9NR96)	Upon confirmation by histopathology, fluorescence based immunohistochemistry was performed in all patients for TLR2 and TLR9, followed by semi-quantitative estimation of the staining intensity and grade of expression. The expression pattern of TLR2 and TLR9 does not vary greatly from normal to precancerous lesions, but a significant variation was observed in advance stages, i.e. squamous cell carcinoma of the uterine cervix. Additionally the expression increased marginally in higher grades.

Clinical Utility of Molecular Biomarkers in Cervical Squamous Intraepithelial Lesions in a Young Adult Population<br><br>Spiryda *et al.*, 2016	50 cervical smears:<br>46 HPV 16 positive<br>4 HPV negative<br>21 normal cytology	IL1-β (P01584)<br>FZD (Q9UP38)<br>N-cadherina (P19022)<br>GDF-15 (Q99988)	Fifty samples were selected that reflected the demographics of the Carolina Women's Care Study participants. IL1β mRNA expression was 9.4-fold higher in cervical cells from women with abnormal Pap tests ($p = .0018$); low-grade squamous intraepithelial lesion had 12.7-fold higher expression than negatives ($p = .0011$). The FZD mRNA expression was 5.7-fold higher in CIN 2 as compared with CIN 1 ($p = .0041$) and 8.5-fold higher compared with cytology/pathology negative ($p = .0014$). Other differences in mRNA expression showed trends but not reaching statistical significance for each condition.
Significance of DNA Replication Licensing Proteins (MCM2, MCM5 and CDC6), p16 and p63 as Markers of Premalignant Lesions of the Uterine Cervix: Its Usefulness to Predict Malignant Potential<br><br>Saritha *et al.*, 2018	190 cervical tissue biopsies<br><br>52 LSIL<br>64 HSIL<br>63 SCCE<br>11 NORMAL TISSUE	MCM2 (P49736)<br>MCM5 (P33992)<br>CDC6 (Q99741)<br>p16 (P42771)<br>p63 (Q9H3D4)	All the markers were positive in malignant and dysplastic cells. MCM protein expression was found to be up-regulated in LSIL, HSIL and in malignancies to a greater extent than p16 as well as p63. CDC6 protein was preferentially expressed in high grade lesions and in invasive squamous cell carcinomas. A progressive increase in the expression of DNA replication licensing proteins in accordance with the grades of cervical intraepithelial lesion suggests these markers as significant to predict malignant potential of low grade lesions in cervical smears.
Biomarkers of resistance to radiation therapy: a	149 patients were included, with FIGO IIB (n = 53) and FIGO IIIB (n	IGF-1R β (P08069)<br>GLUT1 (P11166)<br>HIF-1α (Q16665)	One hundred forty nine patients with a mean age of 46 years were included, with FIGO IIB (n = 53) and FIGO IIIB (n = 96) CCs. 61 patients were treated with

prospective study in cervical carcinoma<br><br>Moreno-Acosta *et al.*, 2017	= 96) CC. 61 patients were treated with exclusive RT + brachytherapy.<br>88 underwent chemoradiotherapy + brachytherapy.	GAPDH (P04406)<br>Survivin (O15392)<br>HKII (P52789)<br>hTERT (O14746)<br>CAIX (Q16790)	exclusive RT + brachytherapy and 88 underwent chemo-radiotherapy + brachytherapy. Our findings suggest an association between hemoglobin level (Hb) (>11 g/dL) and 3 months complete response ($p = 0.02$). Hb level < 11 g/dL was associated with decreased PFS ($p = 0.05$) and OS ($p = 0.08$). Overexpression of IGF-1R β was correlated with a decreased OS ($p = 0.007$). Overexpression of GLUT1 was marginally correlated with reduced OS ($p = 0.05$). PFS and OS were significantly improved in patients undergoing chemoradiation versus exclusive radiotherapy (PFS: $p = 0.04$; OS: $p = 0.01$)
γH2Ax Expression as a Potential Biomarker Differentiating between Low and High Grade Cervical Squamous Intraepithelial Lesions (SIL) and High Risk HPV Related SIL<br><br>Leventakos et al., 2017	In total, 275 cases were included in the study: 112 low grade SIL (LGSIL), 99 high grade SIL (HGSIL), 24 squamous cell carcinoma (SCC), 12 adenocarcinoma and 28 cervical specimens with no essential lesions.	γH2AX (P16104)	Gradual increase of both basal and surface γH2AX expression was noted up from normal cervices to LGSIL harboring a low risk HPV type, to LGSIL harboring a high risk virus at a non-activated state ($p<0.05$). Thereafter, both basal and surface γH2AX expression dropped in LGSIL harboring a high risk virus at an activated state and in HGSIL.
Expression of geminin, p16, and Ki67 in cervical intraepithelial neoplasm and normal tissues	Was examined in 95 samples, including CIN1 (n = 45), CIN2/3 (n = 40), and normal	Geminin (O75496)<br>p16 (P42771)<br>Ki-67 (P46013)	Geminin expression was negative in all normal tissues and expressed in 13.3% of CIN1 and 90.0% of CIN2/3. P16 expression was demonstrated in 24.4% of CIN1 and 87.5% of CIN2/3. The corresponding

Xing *et al.*, 2017	cervical tissues (n = 10) by immunohistochemistry.		Ki67 expression was 35.6% and 95.0%. The specificity of geminin for differentiating between CIN1 and CIN2/3 was 86.7%, while for p16 and Ki67 the corresponding values were 75.6% and 64.4%. The sensitivity of geminin, p16, and Ki67 was 90.0%, 87.5%, and 95.0%, respectively. The positive predictive value (PPV) and accuracy of geminin were higher than p16 and Ki67. In addition, geminin expression showed a weak correlation with HPV status, but there was no association between p16 expression and HPV status.
SIRT1 overexpression in cervical squamous intraepithelial lesions and invasive squamous cell carcinoma<br><br>Vélez-Pérez *et al., 2017*	A total of 101 cases were selected including 29 CIN 1s, 32 CIN 2s, 16 CIN 3s, 2 microinvasive SCCs, and 22 invasive SCCs	Sirtuin 1 (Q96EB6)	Cervical nonneoplastic squamous epithelium showed weak positivity of SIRT1 in the basal layer. SIRT1 cytoplasmic overexpression was found in 13.8% of CIN 1s (4/29), 40.6% of CIN 2s (13/32), and 50% of CIN 3s (8/16), and it was statistically significant between CIN 1 and CIN 2/3 lesions (P=.01). All 24 cases of invasive and microinvasive SCC showed SIRT1 overexpression, with 25% (6/24) showing cytoplasmic staining only, 4.2% (1/24) showing nuclear staining only, and 70.8% (17/24) showing both nuclear and cytoplasmic staining.
A systems approach for the elucidation of crucial genes and network constituents	A Differential expression analysis was performed to identify genes that are	PABPC1 (P11940)<br>RPS27 (P42677)<br>RPL13A (P40429)<br>RPL21 (P46778)	To integrate these DEGs with the pathophysiology of CIN1, assessment of interaction of these gene/proteins and crucial motifs with statistical parameters such as z-score and p-value was carried

of cervical intraepithelial neoplasia 1 (CIN1)<br><br>Suman *et al.*, 2017	differentially expressed in CIN1 in comparison to normal cells. Gene Ontology (GO) and pathway enrichment analyses of DEGs were followed by the construction of a protein-protein interaction (PPI) network. Hubs were identified and module enrichment analysis was performed.	RPL35A (P18077)<br>YWHAZ (P63104)<br>ACTB (P60709)<br>F13A1 (P00488)<br>CRABP2 (P29373)<br>SDHAF2 (Q9NX18)<br>DSP (P15924)<br>KRT14 (P02533)<br>SAA1 (P0DJI8)<br>GJA1 (P17302)<br>MT-CYB (P00156)<br>MAFB (Q9Y5Q3)<br>BCR (P11274)<br>GJB2 (P29033)<br>ADAMTS7 (Q9UKP4)<br>CALML3 (P27482)<br>CXCL5 (P42830)<br>RARRES1 (P49788)<br>HSPB1 (P04792)	out. Microarray analysis revealed 71 differentially expressed genes including 39 upregulated and 32 downregulated genes. 4 genes, namely PABPC1, RPS27, RPL13A and RPL21, were found to be overlapping among hubs and module genes of the PPI network and also part of the significant motifs of the regulatory network. Gene regulation of the DEGs also revealed important TFs and miRNAs such as ELF1, SRF, has-mir-125b-5p and has-mir-644a. PABPC1, RPS27, RPL13A and RPL21 may serve as potential biomarkers for CIN1 and as prospective targets for therapeutic approaches.
Claudin-1 as a Biomarker of Cervical Cytology and Histology<br><br>Benczik *et al.*, 2017	Population of 352 women attending colposcopic referral visits resulting in cervical conisation and a second population of 150 women attending routine gynaecological visits with negative cervical cytology	Claudina-1 (O95832)	High correlation observable between p16INK4a and CLDN1 established CLDN1 as a competing marker in cervical cancer. Concordance of CLDN1 immunostaining of cervical intraepithelial neoplasia 2 and above (CIN2+) positives was 84.0 % (73.8–89.3); concordance of CIN2+ negatives was 69.0 % (59.6–75.8). In conclusion, CLDN1 has similar diagnostic potential as p16INK4a, our results established it as a histological and cytological biomarker with the

			potential to improve the clinical performance of cervical cytology and histology.
Expression of HPV-induced DNA Damage Repair Factors Correlates With CIN Progression<br><br>Spriggs *et al.*, 2018	30 CIN biopsies:<br><br>10 CIN I<br>10 CIN II<br>10 CIN III	pCHK2 (O96017)<br>pCHK1 (O14757)<br>FANCD2 (Q9BXW9)<br>BRCA1 (P38398)<br>H2AX (P16104)	The percentage of cells expressing pCHK2, pCHK1, FANCD2, and BRCA1 is significantly higher in high-grade squamous intraepithelial lesions compared with that of either low-grade squamous intraepithelial lesions or normal tissue, particularly in differentiated cell layers. In addition, the distribution of this staining throughout the epithelium is altered with increasing lesion grade.
Ezrin and E-cadherin expression profile in cervical cytology: a prognostic marker for tumor progression in cervical cancer<br><br>Zacapala-Gómez *et al.*, 2018	Cervical samples of 125 patients. The cytological or histological diagnostic was performed by Papanicolaou staining or H&E staining, respectively.	Ezrin (P15311)<br>E-cadherin (P12830)	High Ezrin expression was observed in cervical cancer samples (70%), samples with multiple infection by HR-HPV (43%), and samples with integrated viral genome (47%). High Ezrin expression was associated with degree of SIL, viral genotype and physical status. In contrast, low E-cadherin expression was found in cervical cancer samples (95%), samples with multiple infection by HR-HPV/LR-HPV (87%) and integrated viral genome (72%). Low E-cadherin expression was associated with degree of SIL and viral genotype. Interestingly, Ezrin nuclear staining was associated with degree of SIL and viral genotype. High Ezrin expression, high percent of nuclear Ezrin and low E-cadherin expression behaved as risk factors for progression to HSIL and cervical cancer.

The distribution of novel biomarkers in carcinoma-in-situ, microinvasive, and squamous cell carcinoma of the uterine cervix<br><br>Nicol *et al*., 2018	60 cases of SCC and normal adjacent tissue. Most tumours were grade 2 (60.4%) or grade 3 (16.0%) and stage I (80.0%) or stage II (18.3%).	**Importin-β (Q14974)**<br>**Exportin 5 (Q9HAV4)**<br>**Mcl 1 (Q07820)**<br>**PDL1 (Q9NZQ7)**<br>**cFLIP (O15519)**<br>E6 (P03126)<br>**E7 (P03129)**<br>p16 (P42771)<br>Ki-67 (P46013)	There was a highly significant increase in PDL1 expression and decrease in Ki-67 (each $p < 0.001$) in microinvasive cancer compared to CIS whereas p16 and E6/E7 remained stable. As the lesion progressed to SCC, p16 and E6/E7 RNA remained strongly overexpressed with a concomitant over expression of importin-β and Ki67. HPV positive Caski cells showed significant elevations of p16, importin-β, exportin-5 and PDL1 compared to the HPV negative cervical cancer cell line C33A, consistent with viral induction of these biomarkers
Minichromosome Maintenance Complex (MCM) Genes Profiling and MCM2 Protein Expression in Cervical Cancer Development<br><br>*Kaur et al., 2019*	evaluated in three cervical tissue samples each of normal cervix, human papillomavirus (HPV)-infected low grade squamous intraepithelial lesion (LSIL), high grade squamous intraepithelial lesion (HSIL) and squamous cell carcinoma (SCC), using Human. Immunohistochemical expression of MCM2	MCM 2 (P49736)<br>MCM 4 (P33991)<br>MCM 5 (P33992)<br>MCM 7 (P33993)	MCM2, 4, 5 and 7 genes expressions were upregulated with increasing fold change during the progression from LSIL to HSIL and the highest in SCC. MCM2 gene had the highest fold change in SCC compared to normal cervix. Immunohistochemically, MCM2 protein was localised in the nuclei of basal cells of normal cervical epithelium and dysplastic-neoplastic cells of CIN and SCC. There was a significant difference in MCM2 protein expression between the histological groups ($P = 0.039$), and histoscore was the highest in HSIL compared to normal cervix ($P = 0.010$).

	protein was semi-quantitatively assessed by histoscore in tissue microarrays containing 9 cases of normal cervix, 10 LSIL, 10 HSIL and 42 cases of SC		
BAG3 expression correlates with the grade of dysplasia in squamous intraepithelial lesions of the uterine cervix<br><br>*Raffone et al., 2019*	Forty patients (16 CIN1/L-SIL, 11 CIN2/H-SIL and 13 CIN3/H-SIL) were assessed by immunohistochemistry for BAG3	BAG3 (O95817)	In all normal controls, BAG3 expression was negative. In L-SIL specimens, BAG3 expression was confined to the basal third of the epithelium, with an intensity minimal in nine cases (56.3%), weak in six (37.5%) and strong in one (6.3%). In H-SIL specimens, BAG3 expression involved also the two upper thirds of the epithelium, with an intensity moderate in 13 cases (54.2%; 8 CIN2 and 5 CIN3) and strong in 11 cases (45.8%; 3 CIN2 and 8 CIN3). The distribution of BAG3 expression correlated perfectly with the grade of dysplasia ($P = 0.0$); a moderate/strong expression of BAG3 was significantly associated with H-SIL ($P < 0.0001$), with no significant difference between CIN2 and CIN3 ($P = 0.1228$)
Regulation of LCoR and RIP140 expression in cervical intraepithelial neoplasia and correlation with CIN progression and dedifferentiation<br><br>*Vogelsang et al., 2020*	81 cervical biopsies with Dx of CIN:<br><br>CIN I= 38<br>CIN II= 26<br>CIN III= 17	LCoR (Q96JN0)<br>RIP140 (P48552)	Nuclear LCoR overexpression correlates significantly with CIN II progression. Nuclear RIP140 expression significantly increases and nuclear LCoR expression decreases with higher grading of cervical intraepithelial neoplasia. Cytoplasmic RIP140 expression is significantly higher in CIN III than in CIN I or CIN II.

**PROTEOMICS ANALYSIS IN SIL AND SCC**			
**BIBLIOGRAPHIES**	**STUDY POPULATION**	**PROTEIN (UNIPROT CODE)**	**FINDINGS**
Proteomic analysis of high-grade dysplastic cervical cells obtained from ThinPrep slides using laser capture microdissection and mass spectrometry<br><br>Gu *et al.*, 2007	9 normal and 9 abnormal cervical cytological specimens	**Overexpressed proteins**<br>14-3-3 protein tau (P27348)<br>RPS3 (P23396)<br>RPS4X (P62701)<br>RPS5 (P46782)<br>RPS8 (P62241)<br>RPSA (P08865)<br>HSPD1 (P10809)<br>RPLP0 (P05388)<br>RPL4 (P36578)<br>RPL7 (P18124)<br>HSPA5 (P11021)<br>CAP1 (Q01518)<br>SLC25A5 (P05141)<br>ORM1 (P02763)<br>SERPINA1 (P01009)<br>A2M (P01023)<br>ACTN1 (P12814)<br>ANXA11 (P50995)<br>ANXA4 (P09525)<br>ANXA5 (P08758)<br>APOA1 (P02647)<br>ATP5F1A (P25705)	Quantitative protein differences between HSIL (High-Grade Squamous Intraepithelial Lesion) and NILM (Negative for Intraepithelial Lesions or Malignancy) samples were determined by comparing the intensities of the representative (label-free) peptide ions. More than 200 proteins were found to exhibit a 3-fold difference in protein level. Interestingly, significant up-regulation of nuclear and mitochondrial proteins in HSIL specimens was noted. In several cases, the increased protein abundance observed in high-grade cells, as determined by quantitative LC-MS, was validated by immunocytochemical methods using ThinPrep cervical specimens.

		ATP5PO (P48047)<br>DDX1 (Q92499)<br>S100A11 (P31949)<br>CANX (P27824)<br>CALR (P27797)<br>CTSG (P08311)<br>CLIC1 (O00299)<br>CS (O75390)<br>CLTC (Q00610)<br>C3 (P01024)<br>MACROH2A1 (O75367)<br>CRB1 (P82279)<br>PRKDC (P78527)<br>DDOST (P39656)<br>RPN1 (P04843)<br>EEF2 (P13639)<br>TUFM (P49411)<br>HSP90B1 (P14625)<br>EPPK1 (P58107)<br>STOM (P27105)<br>FGA (P02671)<br>FGB (P02675)<br>FGG (P02679)<br>FN1 (P02751)<br>FBLN1 (P23142)<br>FLNA (P21333)<br>FLNB (O75369)<br>FBP1 (P09467)	

		ALDOA (P04075)<br>GLUL (P15104)<br>RAN (P62826)<br>HP (P00738)<br>HSPA1L (P34931)<br>HSP90AA1 (P07900)<br>HSP90AB1 (P08238)<br>HNRNPA1 (P09651)<br>HNRNPA3 (P51991)<br>HNRNPD (Q14103)<br>HNRNPF (P52597)<br>HNRNPH1 (P31943)<br>HNRNPK (P61978)<br>HNRNPL (P14866)<br>HNRNPM (P52272)<br>HNRNPR (O43390)<br>HNRNPA2B1 (P22626)<br>HNRNPC (P07910)<br>H1-2 (P16403)<br>H1-5 (P16401)<br>H2AZ1 (P0C0S5)<br>H2BC14 (Q99879)<br>H3-3A (P84243)<br>HOOK1 (Q9UJC3)<br>IGHG2 (P01859)<br>IGKV3-20 (P01619)<br>IGHM (P01871)<br>IDH1 (O75874)	

		IDH2 (P48735)<br>KRT31 (Q15323)<br>KRT18 (P05783)<br>KRT85 (P78386)<br>KRT7 (P08729)<br>KRT8 (P05787)<br>Lamin A/C (P02545)<br>ELANE (P08246)<br>LCP1 (P13796)<br>LYZ (P61626)<br>CAPG (P40121)<br>MDH2 (P40926)<br>MSN (P26038)<br>PRTN3 (P24158)<br>MPO (P05164)<br>MYL2 (P10916)<br>MYH9 (P35579)<br>NEB (P20929)<br>DEFA3 (P59666)<br>NAMPT (P43490)<br>NUMA1 (Q14980)<br>NCL (P19338)<br>NPM1 (P06748)<br>PPIA (P62937)<br>PPIB (P23284)<br>PRDX1 (Q06830)<br>HSD17B4 (P51659)<br>PLEC (Q15149)<br>PTBP1 (P26599)	

		PHB (P35232)<br>PDIA3 (P30101)<br>PDIA4 (P13667)<br>PDIA6 (Q15084)<br>PKM (P14618)<br>IQGAP1 (P46940)<br>RAB11B (Q15907)<br>RAB29 (O14966)<br>RYR3 (Q15413)<br>SPINK5 (Q9NQ38)<br>TF (P02787)<br>SET (Q01105)<br>ATP1A1 (P05023)<br>SFPQ (P23246)<br>HSPA9 (P38646)<br>SOD2 (P04179)<br>TLN1 (Q9Y490)<br>TCP1 (P17987)<br>CCT2 (P78371)<br>CCT4 (P50991)<br>PRDX3 (P30048)<br>TYMP (P19971)<br>TAGLN2 (P37802)<br>TKT (P29401)<br>HADHA (P40939)<br>UBA1 (P22314)<br>VIM (P08670)<br>VCL (P18206)<br>VTN (P04004)	

		VDAC1 (P21796)<br>VDAC2 (P45880)<br>WDR1 (O75083)<br>AZGP1 (P25311)<br><br>**Under-expressed proteins**<br>PGD (P52209)<br>ARF1 (P84077)<br>TMPRSS11D (O60235)<br>AKR1B10 (O60218)<br>ANXA1 (P04083)<br>ANXA3 (P12429)<br>ANXA8 (P13928)<br>SLPI (P03973)<br>ALOX12 (P18054)<br>BRCA2 (P51587)<br>CALML3 (P27482)<br>CBR1 (P16152)<br>CLIC3 (O95833)<br>CSTA (P01040)<br>CSTB (P04080)<br>DSC2 (Q02487)<br>DSG1 (Q02413)<br>DSG3 (P32926)<br>DSP (P15924)<br>EVPL (Q92817)<br>ERO1A (Q96HE7)<br>EIF4A1 (P60842)	

		EZR (P15311)<br>FABP5 (Q01469)<br>LGALS7 (P47929)<br>GSN (P06396)<br>H2AC7 (P20671)<br>SERPINB13 (Q9UIV8)<br>IGHA1 (P01876)<br>IGKV3-20 (P01619)<br>IL1RN (P18510)<br>IVL (P07476)<br>JUP (P14923)<br>KRT13 (P13646)<br>KRT15 (P19012)<br>KRT82 (Q9NSB4)<br>KRT3 (P12035)<br>KRT4 (P19013)<br>KRT6A (P02538)<br>KRT6B (P04259)<br>KHK (P50053)<br>SERPINB1 (P30740)<br>LRP1 (Q07954)<br>SERPINB5 (P36952)<br>MUC5B (Q9HC84)<br>PPL (O60437)<br>PGAM1 (P18669)<br>RNH1 (P13489)<br>PKP1 (Q13835)<br>PKP3 (Q9Y446)<br>PIGR (P01833)	

		TGM1 (P22735)<br>TGM3 (Q08188)<br>NPEPPS (P55786)<br>RAB2A (P61019)<br>CRABP2 (P29373)<br>S100A14 (Q9HCY8)<br>SCEL (O95171)<br>SERPINB12 (Q96P63)<br>SPRR3 (Q9UBC9)<br>SERPINB3 (P29508)	
Characterization of Molecular Markers Indicative of Cervical Cancer Progression<br><br>Arnouk *et al*., 2009	10 healthy tissue surrounding the HSIL<br>10 HSIL<br>10 SCC	**Expressed proteins in HSIL and SCC**<br>CRNN (Q9UBG3)<br>PSME2 (Q9UL46)<br>PARK7 (Q99497)<br>ACTG1 (P63261)<br>TTR (P02766)<br>HSPB1 (P04792)<br>CLIC1 (O00299)<br>KRT8 (P05787)<br>TF (P02787)<br>HSPB6 (O14558)<br>AKR7A2 (O43488)<br>COL1A2 (P08123)<br>CKB (P12277)<br>KRT13 (P13646)<br>GSTP1 (P09211)<br>PSME1 (Q06323)<br>SOD2 (P04179)	Significant expression changes were seen with 53 spots resulting in identification of 23 unique proteins at the molecular level. These include eight that uniquely distinguish normal epithelium and HSIL and four that uniquely distinguish HSIL and carcinoma. In addition, one protein, cornulin, distinguishes all three states. Other identified proteins included differentiation markers, oncogene DJ-1, serpins, stress and interferon-responsive proteins, detoxifying enzymes, and serum transporters.

		LMNA (P02545)<br>SERPINB1 (P30740)<br>SERPINB3 (P29508)<br>KRT10 (P13645)<br>KRT6A (P02538)<br>WARS1 (P23381	
Proteomic patterns of cervical cancer cell lines, a network perspective<br><br>Higareda-Almaraz *et al.*, 2011	A systemic perspective of the common proteomic profile of six cervical cancer cell lines: HeLa y CaLo (HPV 18) SiHa y CaSki (HPV 16) ViBo y C-33ª (HPV-free cancer lines)	Consensus proteins among cervical cancer cell lines<br>HSPA5 (P11021)<br>ENO1 (P06733)<br>HSPA8 (P11142)<br>VIM (P08670)<br>ENO1 (P06733)<br>P4HB (P07237)<br>ANXA2 (P07355)<br>TUBB (P07437)<br>ANXA4 (P09525)<br>TUFM (P49411)<br>TPI1 (P60174)<br>VIM (P08670)<br>PGAM1 (P18669)<br>ATP5F1B (P06576)<br>ACTG1 (P63261)<br>ATIC (P31939)<br>CKB (P12277)<br>EEF2 (P13639)<br>HYOU1 (Q9Y4L1)<br>CKB (P12277)	Analyzed by means of 2D SDS-PAGE and MALDI-TOF mass spectrometry the protein extracts of six cervical cancer cell lines, from which we identified a consensus of 66 proteins. We call this group of proteins, the "central core of cervical cancer". Starting from this core set of proteins, we acquired a PPI network that pointed, through topological analysis, to some proteins that may well be playing a central role in the neoplastic process, such as 14-3-3ζ. In silico overrepresentation analysis of transcription factors pointed to the overexpression of c-Myc, Max and E2F1 as key transcription factors involved in orchestrating the neoplastic phenotype.

		PPIA (P62937)<br>GSN (P06396)<br>VCL (P18206)<br>WDR1 (O75083)<br>EZR (P15311)<br>HSPA1A (P0DMV8)<br>TUBA1C (Q9BQE3)<br>PRDX1 (Q06830)<br>PDIA3 (P30101)<br>HSPA9 (P38646)<br>SDHA (P31040)<br>RPSA (P08865)<br>HSP90AB1 (P08238)<br>ESD (P10768)<br>TKT (P29401)<br>LDHB (P07195)<br>ALDH1A1 (P00352)<br>PPA1 (Q15181)<br>DDX3X (O00571)<br>GLUD1 (P00367)<br>GAPDH (P04406)<br>ACO2 (Q99798)<br>MYL12A (P19105)<br>RRM1 (P23921)<br>EEF2 (P13639)<br>UQCRC1 (P31930)<br>CNN2 (Q99439)<br>PCBP1 (Q15365)<br>IMMT (Q16891)	

		CAPG (P40121)<br>TPT1 (P13693)<br>PSMB4 (P28070)<br>VCP (P55072)<br>KRT1 (P04264)<br>LGALS1 (P09382)<br>YWHAZ (P63104)<br>LMNA (P02545)<br>HNRNPL (P14866)<br>CCT7 (Q99832)<br>OSBPL8 (Q9BZF1)<br>CCT5 (P48643)<br>HYOU1 (Q9Y4L1)<br>PSMA5 (P28066)<br>FOXP3 (Q9BZS1)	
Gelsolin and ceruloplasmin as potential predictive biomarkers for cervical cancer by 2D-DIGE proteomics analysis<br><br>Lokamani *et al.*, 2013	Serum samples of controls, patients with cervical intraepithelial neoplasia grade 3 (CIN 3), squamous cell carcinoma of early (SCC I and II) and late (SCC III and IV) stage were subjected to 2D-DIGE	CD5L (O43866)<br>GSN (P06396)<br>CFH (P08603)<br>CFB (P00751)<br>C1S (P09871)<br>ALB (P02768)<br>AFM (P43652)<br>APOA1 (P02647)<br>APOH (P02749)<br>SERPINA1 (P01009)<br>TF (P02787)<br>HPX (P02790)<br>HP (P00738)<br>CP (P00450)	A total of 20 differentially expressed proteins were identified. These proteins were found to play key roles in the apoptosis pathway, complement system, various types of transportation such as hormones, fatty acids, lipid, vitamin E and drug transportation, coagulation cascade, regulation of iron and immunologic response. Based on their functional relevancy to the progression of various cancers, 4 proteins namely the complement factor H, CD5-like antigen, gelsolin and ceruloplasmin were chosen for further validation using ELISA. Biological network analysis showed that ceruloplasmin and gelsolin are closely interacted with the oncogene NF-κb. These two proteins were further validated using the IHC.

		IGHM (P01871)	
Identification of protein biomarkers for cervical cancer using human cervicovaginal fluid<br><br>Van Raemdonck *et al.*, 2014	**12 samples of cervicovaginal fluids:**<br><br>6 without SIL and HPV<br>4 LSIL (HPV 31/52, 39,18/56, 31/52)<br>2 HSIL (HPV 16, 31)	ACTN4 (O43707)<br>PGK1 (P00558)<br>GC (P02774)<br>CFH (P08603)<br>CRABP2 (P29373)<br>SERPINB3 (P29508)<br>NAMPT (P43490)<br>ACTR3 (P61158)<br>YWHAE (P62258)<br>UBE2D2 (P62837)<br>EEF1A1P5 (Q5VTE0)<br>SERPINB13 (Q9UIV8)<br>HP (P00738)<br>ATP5F1B (P06576)<br>ANXA2 (P07355)<br>PKM (P14618)	The proteome analysis revealed 16 candidate biomarkers of which alpha-actinin-4 ($p = 0.001$) and pyruvate kinase isozyme M1/M2 ($p = 0.014$) were most promising. Verification of alpha-actinin-4 by ELISA ($n = 28$) showed that this candidate biomarker discriminated between samples from healthy and both low-risk and high-risk HPV-infected women ($p = 0.009$). Additional analysis of longitudinal samples ($n = 29$) showed that alpha-actinin-4 levels correlated with virus persistence and clearing, with a discrimination of approximately 18 pg/ml.
A panel of regulated proteins in serum from patients with cervical intraepithelial neoplasia and cervical cancer<br><br>Boichenko *et al.*, 2014	Serum of patients with cervical intraepithelial neoplasia (CIN) and squamous cell cervical cancer using iTRAQ, label-free shotgun, and targeted mass-spectrometric quantification	**Up-regulated proteins**<br>IGHA1 (P01876)<br>HP (P00738)<br>IGHG3 (P01860)<br>SERPINA1 (P01009)<br>PF4 (P02776)<br>CRP (P02741)<br>FCGBP (Q9Y6R7)<br>PROS1 (P07225)<br>IGKV1-5 (P01602)	In the discovery stage we used a "pooling" strategy for the comparative analysis of immunodepleted serum and revealed 15 up- and 26 down-regulated proteins in patients with early- (CES) and late-stage (CLS) cervical cancer. The analysis of nondepleted serum samples from patients with CIN, CES, an CLS and healthy controls showed significant changes in abundance of alpha-1-acid glycoprotein 1, alpha-1-antitrypsin, serotransferrin, haptoglobin, alpha-2-HS-glycoprotein, and vitamin D-binding protein. We

		CRP (P02741)<br>FCGBP (Q9Y6R7)<br>HP (P00738)<br>LBP (P18428)<br>IGHG3 (P01860)<br>LGALS3BP (Q08380)<br>A1AG1 (P02763)<br>C6 (P13671)<br><br>**down-regulated proteins**<br>K1C10 (P13645)<br>K1C9 (P35527)<br>S100A7 (P31151)<br>S100A9 (P06702)<br>KRT5 (P13647)<br>C1R (P00736)<br>KRT2 (P35908)<br>PRG4 (Q92954)<br>LCAT (P04180)<br>KRT1 (P04264)<br>C6 (P13671)<br>CFI (P05156)<br>FBLN1 (P23142)<br>S10A7 (P31151)<br>PZP (P20742)<br>KV106 (P01598)<br>IPSP (P05154)<br>APOA4 (P06727)	validated our findings using a fast UHPLC/MRM method in an independent set of serum samples from patients with cervical cancer or CIN and healthy controls as well as serum samples from patients with ovarian cancer (more than 400 samples in total).

		KAIN (P29622)<br>K2C5 (P13647)<br>CD14 (P08571)<br>FBLN1 (P23142)<br>LCAT (P04180)<br>K1C10 (P13645)<br>PROS (P07225)<br>IGKC (P01834)<br>TRFE (P02787)	
Differentially expressed proteins among normal cervix, cervical intraepithelial neoplasia and cervical squamous cell carcinoma<br><br>Zhao *et al*., 2015	Cervical tissues (including normal cervix, CIN and CSCC)<br><br>9 cancer-free tissue<br>7 CIN I<br>8 CIN II<br>8 CIN III<br>7 SCC	IGHG1 (P01857)<br>SHFL (Q9NUL5)<br>F7 (P08709)<br>ALB (P02768)<br>EEF1A1 (P68104)<br>PPP1R7 (Q15435)<br>SOD3 (P08294)<br>TTR (P02766)<br>TPSB2 (P20231)<br>TPSAB1 (Q15661)<br>ALB (P02768)<br>HBB (P68871)<br>IGKC (P01834)<br>PKM (P14618)<br>APOA1 (P02647)<br>S100A11 (P31949)<br>PKM (P14618)<br>S100A8 (P05109)<br>TUBB (P07437)<br>S100A9 (P06702)	2-D DIGE images with high resolution and good repeatability were obtained. Forty-six differentially expressed proteins (27 up-regulated and 19 down-regulated) were differentially expressed among the normal cervix, CIN and CSCC. 26 proteins were successfully identified by MALDI-TOF/TOF MS. S100A9 (S100 calcium-binding protein A9) was the most significantly up-regulated protein. Eukaryotic elongation factor 1-alpha-1 (eEF1A1) was the most significantly down-regulated protein. Pyruvate kinase isozymes M2 (PKM2) was both up-regulated and down-regulated

		GSTP1 (P09211)<br>S100A7 (P31151)<br>ENO1 (P06733)<br>PPIA (P62937)	
iTRAQ-based quantitative proteomic analysis of cervical cancer<br><br>Ding *et al*., 2015	**16 biopsias de tejido cervical:**<br><br>8 de cáncer cervical<br>8 de tejido libre de cáncer	OGN (P20774)<br>CRNN (Q9UBG3)<br>DCN (P07585)<br>PRELP (P51888)<br>COL1A1 (P02452)<br>TPM1 (P09493)<br>CNN1 (P51911)<br>SERPINA1 (P01009)<br>ABI3BP (Q7Z7G0)<br>AGR2 (O95994)<br>ORM2 (P19652)<br>BGN (P21810)<br>APOA2 (P02652)<br>DPT (Q07507)<br>APOH (P02749)<br>TAGLN (Q01995)<br>SOD3 (P08294)<br>COL1A2 (P08123)<br>FMOD (Q06828)<br>PTGDS (P41222)<br>KRT1 (P04264)<br>ASPN (Q9BXN1)<br>CP (P00450)<br>SLPI (P03973)<br>CAPS (Q13938)	As a result, 3,647 proteins were identified, among which the expression levels of 294 proteins in cervical cancer samples were distinct from the paired non-tumor samples. Further validation of the differentially expressed proteins, including G6PD, ALDH3A1, STAT1 and HSPB1, was carried out via qRT-PCR, western blot analysis and tissue microarray. Functional analysis of one of the highly expressed proteins, G6PD, was performed using RNA interference. Attenuated G6PD expression reduced the capacity of HeLa cells to migrate and invade in vitro.

		KNG1 (P01042)<br>LGALS1 (P09382)<br>CAVIN1 (Q6NZI2)<br>CPA3 (P15088)<br>FABP3 (P05413)<br>DEK (P35659)<br>GCLM (P48507)<br>SERPINB5 (P36952)<br>WARS1 (P23381)<br>DIAPH1 (O60610)<br>ATP2A2 (P16615)<br>DTX3L (Q8TDB6)<br>MCM6 (Q14566)<br>STAT1 (P42224)<br>SLC2A1 (P11166)<br>KRT17 (Q04695)<br>S100A2 (P29034)<br>ADH1C (P00326)<br>CAMP (P49913)<br>GBP1 (P32455)<br>G6PD (P11413)<br>ALDH3A1 (P30838)<br>OAS3 (Q9Y6K5)<br>MPO (P05164)<br>TAPBP (O15533)<br>PGD (P52209)<br>AKR1B10 (O60218)<br>H1-5 (P16401)<br>EPX (P11678)	

		CES1 (P23141)<br>PRG3 (Q9Y2Y8)<br>NAMPT (P43490)<br>ADH7 (P40394)<br>DEFA1 (P59665<br>AKR1C2 (P52895)	
Differential proteins among normal cervix cells and cervical cancer cells with HPV-16 infection, through mass spectrometry-based Proteomics (2D-DIGE) in women from Southern México<br><br>Serafín-Higuera *et al.*, 2016	A pool of 6 samples from women diagnosed with HPV-16 and histopathologically staged as squamous cell carcinoma (SCC) of which 4 cases were stage IIB, 1 case stage IB1 and 1 case stage IB2. The second group consisted of four combined samples from women with normal cytology and colposcopy and negative for HPV infection.	**Up-regulated proteins**<br>OGN (P20774)<br>ACTA2 (P62736)<br>LUM (P51884)<br><br>**down-regulated proteins**<br>PRDX1 (Q06830)<br>SFN (P31947)<br>ENO1 (P06733)<br>KRT5 (P13647)	The proteins that showed an increased expression in cervical cancer in comparison with normal cervix cells were: Mimecan, Actin from aortic smooth muscle and Lumican. While Keratin, type II cytoskeletal 5, Peroxiredoxin-1 and 14-3-3 protein sigma showed a decrease in their protein expression level in cervical cancer in comparison with normal cervix cells.
Tyrosine kinase LYN is an oncotarget in human cervical cancer: A quantitative proteomic based study	The tissue microarray slides containing malignant and normal cervical tissues (n=208) was purchased from US Biomax, Inc.	LYZ (P61626)<br>PCID2 (Q5JVF3)<br>TMEM214 (Q6NUQ4)<br>BID (P55957)<br>NCKAP1L (P55160)<br>RFC5 (P40937)	Therefore, we employed iTRAQ to obtain novel proteins profile which participates in the tumor oncogenesis of cervical cancer. 3300 proteins were identified aberrantly expressed in cervical cancer, and western bolt was performed to validate the results of iTRAQ. Then, we selected LYN for further

Liu *et al.*, 2016		CYBB (P04839)<br>LYN (P07948)<br>SSR3 (Q9UNL2)<br>DYNLT1 (P63172)<br>SRSF3 (P84103)<br>DEK (P35659)<br>RQCD1 (Q92600)<br>ITGB2 (P05107)<br>TECR (Q9NZ01)<br>SYNM (O15061)<br>LAMB2 (P55268)<br>PEX5 (P50542)<br>S100A4 (P26447)<br>ACTA2 (P62736)<br>IGHV3-49<br>(A0A0A0MS15)<br>MAP1S (Q66K74)<br>IQGAP2 (Q13576)<br>APOA4 (P06727)<br>RPLP2 (P05387)<br>AK3 (Q9UIJ7)<br>SEPT11 (Q9NVA2)<br>BIN1 (O00499)<br>CD14 (P08571)<br>AKAP2 (Q9Y2D5)	study. Immunohistochemistry identified that LYN expression was significantly increased in cervical cancer tissues than that in cancer adjacent normal cervical tissues and normal cervical tissues. The increased LYN expression was significantly correlated with cancer differentiation and FIGO stage. Silencing LYN inhibited cell proliferation, migration and invasion, conversely, overexpression LYN promoted cell proliferation, migration and invasion
Proteomic identification of potential biomarkers for cervical squamous cell	Collected 76 cases of fresh cervical tissues and 116 cases of paraffin-embedded	ASAH1 (Q13510)<br>MCM5 (P33992)<br>CYCS (P99999)<br>TM9SF4 (Q92544)	We identified 67 proteins that were differentially expressed in human papillomavirus 16-positive squamous cell carcinoma compared to normal cervix. The quantitative reverse transcriptase-polymerase

carcinoma and human papillomavirus infection<br><br>Qing et al., 2017	tissue slices, diagnosed as cervical squamous cell carcinoma, cervical intraepithelial neoplasia II-III, or normal cervix.	ANP32A (P39687)<br>SRSF7 (Q16629)<br>RBM8A (Q9Y5S9)<br>SRSF10 (O75494)<br>PCBP2 (Q15366)<br>OSTF1 (Q92882)<br>TAGLN2 (P37802)<br>PCNA (P12004)<br>MCM2 (P49736)<br>HSPD1 (P10809)<br>HNRNPC (P07910)<br>CYBB (P04839)<br>HNRNPA1 (P09651)<br>ENO1 (P06733)<br>NCL (P19338)<br>TYMP (P19971)<br>PFN1 (P07737)<br>ATP6V1B2 (P21281)<br>DDX5 (P17844)<br>ANP32B (Q92688)<br>SCPEP1 (Q9HB40)<br>AP3D1 (O14617)<br>SUMO2 (P61956)<br>MDH2 (P40926)<br>SRP9 (P49458)<br>VDAC1 (P21796)<br>MCM4 (P33991)<br>HSPA9 (P38646)<br>GSTK1 (Q9Y2Q3)	chain reaction analysis verified the upregulation of ASAH1, PCBP2, DDX5, MCM5, TAGLN2, hnRNPA1, ENO1, TYPH, CYC, and MCM4 in squamous cell carcinoma compared to normal cervix ($p < 0.05$). In addition, the transcription of PCBP2, MCM5, hnRNPA1, TYPH, and CYC was also significantly increased in cervical intraepithelial neoplasia II-III compared to normal cervix. Immunohistochemistry staining further confirmed the overexpression of PCBP2, hnRNPA1, ASAH1, and DDX5 in squamous cell carcinoma and cervical intraepithelial neoplasia II-III compared to normal controls ($p < 0.05$).

		PRELP (P5188)<br>LUM (P51884)<br>POTEKP (Q9BYX7)<br>COL5A1 (P20908)<br>PTGDS (P41222)<br>SOD3 (P08294)<br>TGM2 (P21980)<br>DCN (P07585)<br>FBN1 (P35555)<br>FBLN1 (P23142)<br>OLFML3 (Q9NRN5)<br>TNXB (P22105)<br>KANK2 (Q63ZY3)<br>AGT (P01019)<br>COL6A2 (P12110)<br>SERPINA1 (P01009)<br>AMBP (P02760)<br>COL6A3 (P12111)<br>COL6A1 (P12109)<br>APOA2 (P02652)<br>CLU (P10909)<br>SPRR1A (P35321)<br>DPT (Q07507)<br>COL5A2 (P05997)<br>COL1A1 (P02452)<br>COL1A2 (P08123)<br>COL14A1 (Q05707)<br>SERPINA3 (P01011)<br>COL21A1 (Q96P44)	

		CKB (P12277)<br>CAV1 (Q03135)<br>KRT6B (P04259)<br>COL3A1 (P02461)<br>CRNN (Q9UBG3)	
Proteomic Analysis of Normal and Cancer Cervical Cell Lines Reveals Deregulation of Cytoskeleton-associated Proteins<br><br>Pappa *et al.*, 2017	Cell extracts from a normal cervical (HCK1T) and three cervical cancer cell lines, one HPV-negative (C33A), and two HPV-positive, SiHa (HPV16+) and HeLa (HPV18+), were analyzed by 2-dimensional electrophoresis and differentially expressed proteins were identified by MALDI-TOF mass spectrometry	RPSA (P08865)<br>EEF1D (P29692)<br>HSPD1 (P10809)<br>CCT5 (P48643)<br>HSPD1 (P10809)<br>PPA1 (Q15181)<br>PDIA3 (P30101)<br>LDHB (P07195)<br>NME1 (P15531)<br>TCP1 (P17987)<br>SELENBP1 (Q13228)<br>CCT2 (P78371)<br>ENO1 (P06733)<br>TPI1 (P60174)<br>LMNA (P02545)<br>ERP29 (P30040)<br>FABP5 (Q01469)<br>LMNA (P02545)<br>PHGDH (O43175)<br>EEF1G (P26641)<br>HINT2 (Q9BX68)<br>CCT6A (P40227)<br>STIP1 (P31948)<br>LMNA (P02545)	In total, 113 proteins were found differentially expressed between the normal and the cervical cancer lines. Bioinformatics analysis revealed the actin cytoskeleton signaling pathway to be significantly affected, while up-regulation of cofilin-1, an actin depolymerizing factor, was documented and further validated by western blotting. Furthermore, two-way comparisons among the four cell lines, revealed a set of 18 informative differentially expressed proteins.

		ENO1 (P06733)<br>STIP1 (P31948)<br>PHGDH (O43175)<br>PGAM1 (P18669)<br>GMPS (P49915)<br>EEF2 (P13639)<br>PSMA2 (P25787)<br>KRT1 (P04264)<br>RACK1 (P63244)<br>RAN (P62826)<br>HSD17B10 (Q99714)<br>PPIA (P62937)<br>PRDX5 (P30044)<br>PPIA (P62937)<br>PKM (P14618)<br>ALDOA (P04075)<br>LDHA (P00338)<br>GAPDH (P04406)<br>PRDX1 (Q06830)<br>CFL1 (P23528)<br>KRT7 (P08729)<br>APRT (P07741)<br>RPA2 (P15927)<br>ERP29 (P30040)<br>ANKRD36BP1 (Q96IX9)<br>PARK7 (Q99497)<br>CCT2 (P78371)<br>LHB (P01229)	

		OXCT1 (P55809)<br>KRT10 (P13645)<br>PNP (P00491)<br>PSMA2 (P25787)<br>AKR1B1 (P15121)<br>LHB (P01229)<br>IDH1 (O75874)<br>PEBP1 (P30086)<br>LGALS3 (P17931)<br>ATP5PD (O75947)<br>PHB (P35232)<br>PRDX2 (P32119)<br>HSPD1 (P10809)<br>GRB2 (P62993)<br>PRDX3 (P30048)<br>ALDH7A1 (P49419)<br>RAN (P62826)<br>TPI1 (P60174)<br>ALDH7A1 (P49419)<br>TUFM (P49411)<br>PSMB2 (P49721)<br>ETFA (P13804)<br>RACK1 (P63244)<br>CFL1 (P23528)<br>VDAC2 (P45880)<br>PSMA4 (P25789)<br>VDAC1 (P21796)<br>ETFB (P38117<br>PRDX6 (P30041)	

		PSMA7 (O14818)<br>SFN (P31947)<br>VIM (P08670)<br>VCP (P55072)<br>HSPA8 (P11142)<br>HSPB1 (P04792)<br>SERPINB5 (P36952)<br>HNRNPH1 (P31943)<br>CAPG (P40121)<br>KRT6A (P02538)<br>WDR1 (O75083)<br>ATIC (P31939)<br>PDLIM1 (O00151)<br>KRT5 (P13647)<br>FSCN1 (Q16658)<br>KRT6A (P02538)<br>KRT6B (P04259)<br>KRT6C (P48668)<br>TRIP6 (Q15654)<br>ISG15 (P05161)<br>TKT (P29401)<br>CKMT1A (P12532)<br>ANXA2 (P07355)<br>ANXA2P2 (A6NMY6)<br>CALR (P27797)<br>P4HB (P07237)<br>HSPA5 (P11021)<br>EIF3I (Q13347)<br>XRCC5 (P13010)	

		RPLP0 (P05388)<br>VCL (P18206)<br>ACTG1 (P63261)<br>IMPDH2 (P12268)<br>FUBP1 (Q96AE4)<br>HSPA9 (P38646)<br>HSPA6 (P17066)<br>TUBB (P07437)<br>TUBB4B (P68371)<br>ATP5F1B (P06576)<br>TUBB4A (P04350)<br>TUBB2A (Q13885)<br>KRT8 (P05787)<br>KRT19 (P08727)<br>PGK1 (P00558)<br>TAGLN2 (P37802)<br>ATP5F1B (P06576)<br>TUBB2B (Q9BVA1)<br>PDIA6 (Q15084)<br>ATP5F1A (P25705)<br>SHMT2 (P34897)	
Proteomic alterations in early stage cervical cancer<br><br>Güzel *et al.*, 2018	We compared in a semi-quantitative way the proteomes from an equivalent of 8,000 tumor cells from patients with squamous cell cervical cancer (SCC, n = 22) with	MCM4 (P33991)<br>S100P (P25815)<br>POLD1 (P28340)<br>DNAJC13 (O75165)<br>RFC2 (P35250)<br>PTK7 (Q13308)<br>RIC8A (Q9NPQ8)<br>SPECC1 (Q5M775)	

	healthy epithelial and stromal cells obtained from normal cervical tissue (n = 13)	LSM2 (Q9Y333)<br>ADPGK (Q9BRR6)<br>EYA3 (Q99504)<br>GALNT2 (Q10471)<br>PCNP (Q8WW12)<br>HLA-DRB1 (P01911)<br>DTX3L (Q8TDB6)<br>KPNA2 (P52292)<br>CTPS1 (P17812)<br>P3H1 (Q32P28)<br>PTRH2 (Q9Y3E5)<br>SEP15 (O60613)<br>ICAM1 (P05362)<br>ACOT9 (Q9Y305)<br>PARP9 (Q8IXQ6)<br>NUP210 (Q8TEM1)<br>CEACAM5 (P06731)<br>SMCHD1 (A6NHR9)<br>ZNF326 (Q5BKZ1)<br>RNF20 (Q5VTR2)<br>RHOT2 (Q8IXI1)<br>SAMD9 (Q5K651)<br>MPO (P05164)<br>KRT13 (P13646)<br>EVPL (Q92817)<br>CRNN (Q9UBG3)<br>DSC2 (Q02487)<br>ANP32A (P39687)<br>TOP1 (P11387)	

		TMX3 (Q96JJ7)<br>PDIA3 (P30101)<br>MCM4 (P33991)<br>MCM6 (Q14566)<br>MCM7 (P33993)<br>DSG1 (Q02413)<br>RRBP1 (Q9P2E9)<br>CSE1L (P55060)<br>MCM3 (P25205)<br>HNRNPA2B1 (P22626)<br>KRT5 (P13647)<br>HSPA5 (P11021)<br>CALR (P27797)<br>TPR (P12270)<br>MCM2 (P49736)<br>ERP29 (P30040)<br>NUP155 (O75694)<br>PLAA (Q9Y263)<br>FEN1 (P39748)<br>WARS1 (P23381)<br>PRPF6 (O94906)<br>S100P (P25815)<br>POLD1 (P28340)<br>SERPINH1 (P50454)	
Microproteomic Profiling of High-Grade Squamous Intraepithelial Lesion of the Cervix: Insight into	Tissue regions of endoC, ectoC, and HSlL were collected by laser microdissection (3500	Q9NVP2 (ASF1B)<br>P07948 (LYN)<br>Q04695 (KRT17)<br>O00151 (DDX39A)	We identified 3072 proteins among the fifteen samples and 2386 were quantified in at least four out of the five biological replicates of at least one tissue type. We found 236 proteins more abundant in HSIL.

Biological Mechanisms of Dysplasia and New Potential Diagnostic Markers<br><br>Pottier *et al.,* 2019	cells each) from five patients. Samples were processed and analyzed using our recently developed laser microdissection-based microproteomic method. Tissues were compared in order to retrieve HSIL's proteomic profile	O00267 (SUPT5H)<br>O15143 (ARPC1B)<br>O43143 (DHX15)<br>O43670 (ZNF207)<br>O43684 (BUB3)<br>O43795 (MYO1B)<br>O60216 (RAD21)<br>O60264 (SMARCA5)<br>O60610 (DIAPH1)<br>Q99879 (H2BC14)<br>O75175 (CNOT3)<br>O75494 (SRSF10)<br>O75694 (NUP155)<br>O94776 (MTA2)<br>O94906 (PRPF6)<br>O95232 (LUC7L3)<br>O96019 (ACTL6A)<br>P00734 (F2)<br>P00747 (PLG)<br>P00915 (CA1)<br>P02042 (HBD)<br>P02671 (FGA)<br>P02675 (FGB)<br>P02679 (FGG)<br>P02746 (C1QB)<br>P02749 (APOH)<br>P02763 (ORM1)<br>P02786 (TFRC)<br>P04439 (HLA-A)	Gene ontology enrichments revealed mechanisms of DNA replication and RNA splicing. Despite the squamous nature of HSIL, a common signature between HSIL and endoC could be found. Finally, potential new markers could support diagnosis of dysplasia in SILs.

		Q93077 (H2AC6)<br>P05141 (SLC25A5)<br>P05164 (MPO)<br>P06731 (CEACAM5)<br>P06733 (ENO1)<br>P06753 (TPM3)<br>P07437 (TUBB)<br>P08621 (SNRNP70)<br>P09651 (HNRNPA1)<br>P09874 (PARP1)<br>Q71UI9 (H2AZ2)<br>P10586 (PTPRF)<br>P11387 (TOP1)<br>P11586 (MTHFD1)<br>P11908 (PRPS2)<br>P12004 (PCNA)<br>P12270 (TPR)<br>P12532 (CKMT1A)<br>P13796 (LCP1)<br>P15121 (AKR1B1)<br>P15927 (RPA2)<br>P16333 (NCK1)<br>P16401 (H1-5)<br>P16615 (ATP2A2)<br>P16949 (STMN1)<br>P17301 (ITGA2)<br>P17844 (DDX5)<br>P19971 (TYMP)<br>P20700 (LMNB1)	

		P21397 (MAOA)<br>P22087 (FBL)<br>P22102 (GART)<br>P25205 (MCM3)<br>P26583 (HMGB2)<br>P26639 (TARS1)<br>P27105 (STOM)<br>P27694 (RPA1)<br>P28065 (PSMB9)<br>P28340 (POLD1)<br>P30520 (ADSS2)<br>P31025 (LCN1)<br>P31431 (SDC4)<br>P31930 (UQCRC1)<br>P32455 (GBP1)<br>P32456 (GBP2)<br>P33240 (CSTF2)<br>P33241 (LSP1)<br>P33316 (DUT)<br>P33991 (MCM4)<br>P33992(MCM5)<br>P33993 (MCM7)<br>P34897 (SHMT2)<br>P35244 (RPA3)<br>P35573 (AGL)<br>P35659 (DEK)<br>P36551 (CPOX)<br>P37802 (TAGLN2)<br>P38919 (EIF4A3)	

		P39748 (FEN1)<br>P40199 (CEACAM6)<br>P40763 (STAT3)<br>P41091 (EIF2S3)<br>P42166 (TMPO)<br>P42167 (TMPO)<br>P42224 (STAT1)<br>P42285 (MTREX)<br>P43246 (MSH2)<br>GPD2 (P43304)<br>SYK (P43405)<br>RANBP1 (P43487)<br>VDAC2 (P45880)<br>CBX5 (P45973)<br>RANGAP1 (P46060)<br>RECQL (P46063)<br>GCLC (P48506)<br>NASP (P49321)<br>MCM2 (P49736)<br>GMPS (P49915)<br>MRE11 (P49959)<br>FXR1 (P51114)<br>SMARCA4 (P51532)<br>RPS6KA3 (P51812)<br>HNRNPF (P52597)<br>THOP1 (P52888)<br>AKR1C2 (P52895)<br>NAP1L1 (P55209)<br>EPPK1 (P58107)	

		DEFA3 (P59666)<br>DEFA1 (P59665)<br>RAP2B (P61225)<br>RAP2C (Q9Y3L5)<br>RAP2A (P10114)<br>MAGOH (P61326)<br>LSM3 (P62310)<br>H4C1 (P62805)<br>RAN (P62826)<br>RPS26 (P62854)<br>RPS26P11 (Q5JNZ5)<br>PPIA (P62937)<br>PPIAL4A (Q9Y536)<br>PPIAL4D (F5H284)<br>TRA2B (P62995)<br>UBE2I (P63279)<br>HBB (P68871)<br>HBA1 (P69905)<br>GTF2I (P78347)<br>GTF2IRD2 (Q86UP8)<br>GTF2IRD2B (Q6EKJ0)<br>PRKDC (P78527)<br>HNRNPU (Q00839)<br>U2AF1 (Q01081)<br>U2AF1L4 (Q8WU68)<br>FKBP4 (Q02790)<br>TNFAIP2 (Q03169)<br>TAP1 (Q03518)<br>TAP2 (Q03519	

		SSBP1 (Q04837)<br>SSRP1 (Q08945)<br>NSUN2 (Q08J23)<br>RBBP4 (Q09028)<br>NCBP1 (Q09161)<br>GTF3C1 (Q12789)<br>SF3A3 (Q12874)<br>PPP1R8 (Q12972)<br>HNRNPA0 (Q13151)<br>CBX3 (Q13185)<br>TRIM28 (Q13263)<br>G3BP1 (Q13283)<br>EIF3I (Q13347)<br>TCOF1 (Q13428)<br>HDAC1 (Q13547)<br>CBFB (Q13951)<br>COTL1 (Q14019)<br>CAPRIN1 (Q14444)<br>RBM39 (Q14498)<br>RBM23 (Q86U06)<br>PRPSAP1 (Q14558)<br>MCM6 (Q14566)<br>SMC1A (Q14683)<br>USP10 (Q14694)<br>CHD4 (Q14839)<br>CHD5 (Q8TDI0)<br>CHD3 (Q12873)<br>PAFAH1B3 (Q15102)<br>PMVK (Q15126)	

		NONO (Q15233)<br>SF3B3 (Q15393)<br>RPS6KA1 (Q15418)<br>RPS6KA2 (Q15349)<br>SAFB (Q15424)<br>MAPRE1 (Q15691)<br>ELAVL1 (Q15717)<br>RBBP7 (Q16576)<br>CPSF6 (Q16630)<br>IFI16 (Q16666)<br>PYHIN1 (Q6K0P9)<br>(SMU1) Q2TAY7<br>LARP7 (Q4G0J3)<br>ARHGEF16 (Q5VV41)<br>RNF213 (Q63HN8)<br>PRPF8 (Q6P2Q9)<br>DDX46 (Q7L014)<br>MYH14 (Q7Z406)<br>IRF2BP2 (Q7Z5L9)<br>FERMT3 (Q86UX7)<br>ERC1 (Q8IUD2)<br>SRRM1 (Q8IYB3)<br>NUP93 (Q8N1F7)<br>SMARCC2 (Q8TAQ2)<br>DTX3L (Q8TDB6)<br>NUP210 (Q8TEM1)<br>GATAD2B (Q8WXI9)<br>DNAJC9 (Q8WXX5)<br>IRGQ (Q8WZA9)	

		DDB2 (Q92466)<br>RTF1 (Q92541)<br>ANP32B (Q92688)<br>RAD50 (Q92878)<br>ARHGEF1 (Q92888)<br>SMARCC1 (Q92922)<br>SMARCE1 (Q969G3)<br>EFHD2 (Q96C19)<br>HNRNPAB (Q99729)<br>NAP1L4 (Q99733)<br>ANP32E (Q9BTT0)<br>MRPL4 (Q9BYD3)<br>API5 (Q9BZZ5)<br>BOLA2 (Q9H3K6)<br>EPB41L1 (Q9H4G0)<br>FN3KRP (Q9HA64)<br>HMG20A (Q9NP66)<br>RPRD1B (Q9NQG5)<br>DDX21 (Q9NR30)<br>DDX50 (Q9BQ39)<br>SLTM (Q9NWH9)<br>ERAP1 (Q9NZ08)<br>MAT2B (Q9NZL9)<br>KIAA1522 (Q9P206)<br>RCC2 (Q9P258)<br>SAE1 (Q9UBE0)<br>UBA2 (Q9UBT2)<br>TJP2 (Q9UDY2)<br>SUN2 (Q9UH99)	

		LIMA1 (Q9UHB6)<br>BAZ1B (Q9UIG0)<br>RALY (Q9UKM9)<br>ACIN1 (Q9UKV3)<br>CORO1C (Q9ULV4)<br>SMC3 (Q9UQE7)<br>CARHSP1 (Q9Y2V2)<br>THRAP3 (Q9Y2W1)<br>NOP58 (Q9Y2X3)<br>LUC7L2 (Q9Y383)<br>LUC7L (Q9NQ29)<br>SH3BP1 (Q9Y3L3)<br>SAMHD1 (Q89Y3Z3)<br>SUPT16H (Q9Y5B9)<br>TIMM13 (Q9Y5L4)<br>NPTN (Q9Y639)<br>BZW2 (Q9Y6E2)	
Label-free cervicovaginal fluid proteome profiling reflects the cervix neoplastic transformation<br><br>Starodubtseva *et al.*, 2019	The proteome composition of CVF from 40 women of reproductive age with human papillomavirus (HPV)-associated cervix neoplastic transformation (low-grade squamous intraepithelial lesion [LSIL], high-grade squamous	YWHAZ (P63104)<br>CAP1 (Q01518)<br>AFM (P43652)<br>SERPINA3 (P01011)<br>A2ML1 (A8K2U0)<br>ACTN4 (O43707)<br>AMY1A (P04745)<br>ANXA1 (P04083)<br>ANXA2 (P07355)<br>APOA1 (P02647)<br>BPIFB1 (Q8TDL5)<br>FGA (P02671)	Hierarchical clustering and principal component analysis (PCA) of the proteomic data obtained by a label-free quantitation approach show the distribution of the sample set between four major clusters (no intraepithelial lesion or malignancy [NILM], LSIL, HSIL and CANCER) depending on the form of cervical lesion. Multisample ANOVA with subsequent Welch's t test resulted in 117 that changed significantly across the four clinical stages, including 27 proteins significantly changed in cervical cancer. Some of them were indicated as promising biomarkers previously (ACTN4, VTN, ANXA1,

	intraepithelial lesion [HSIL], and CANCER) was investigated	FN1 (P02751)<br>LGALS3BP (Q08380)<br>HRG (P04196)<br>H1-4 (P10412)<br>IGHG3 (P01860)<br>FCGBP (Q9Y6R7)<br>JCHAIN (P01591)<br>LYZ (P61626)<br>MUC5B (Q9HC84)<br>MPO (P05164)<br>ELANE (P08246)<br>NAMPT (P43490)<br>PON1 (P27169)<br>TTR (P02766)<br>VTN (P04004)<br>ACTC1 (P68032)<br>ORM2 (P19652)<br>SERPINA1 (P01009)<br>A1BG (P04217)<br>SERPINC1 (P01008)<br>APOB (P04114)<br>C4BPA (P04003)<br>C4B (P0C0L5)<br>CFB (P00751)<br>CDK3 (Q00526)<br>CSTA (P01040)<br>DSC2 (Q02487)<br>PI3 (P19957)<br>FABP5 (Q01469)	CAP1, ANXA2, and MUC5B). CVF proteomic data from the discovery stage were analyzed by the partial least squares-discriminant analysis (PLS-DA) method to build a statistical model, allowing to differentiate severe dysplasia (HSIL and CANCER) from the mild/normal stage (NILM and LSIL), and receiver operating characteristic (ROC) area under the curve (AUC) were obtained on an independent set of 33 samples

		GPI (P06744)<br>HP (P00738)<br>HBA1 (P69905)<br>H2BC4 (P62807)<br>KLK10 (O43240)<br>KLK6 (Q92876)<br>KNG1 (P01042)<br>PIGR (P01833)<br>ACP3 (P15309)<br>TGM3 (Q08188)<br>SERPINB13 (Q9UIV8)<br>ALB (P02768)<br>QSOX1 (O00391)<br>SBSN (Q6UWP8)<br>ACTG1 (P63261)<br>ORM1 (P02763)<br>AHSG (P02765)<br>A2M (P01023)<br>ENO1 (P06733)<br>SLPI (P03973)<br>CTSV (O60911)<br>C3 (P01024)<br>SPRR1A (P35321)<br>SPRR1B (P22528)<br>CRNN (Q9UBG3)<br>CRISP3 (P54108)<br>CDA (P32320)<br>FGB (P02675)<br>FGG (P02679)	

		GGCT (O75223)<br>GSN (P06396)<br>HPX (P02790)<br>IGHG2 (P01859)<br>IGHG4 (P01861)<br>IGHM (P01871)<br>IVL (P07476)<br>KLK11 (Q9UBX7)<br>LRG1 (P02750)<br>SERPINB1 (P30740)<br>LCN2 (P80188)<br>PPL (O60437)<br>SERPING1 (P05155)<br>LCP1 (P13796)<br>TGM1 (P22735)<br>SPINK7 (P58062)<br>SERPINB3 (P29508)<br>SPRR3 (Q9UBC9)<br>TXN (P10599)<br>TKT (P29401)<br>GC (P02774)<br>WFDC2 (Q14508)<br>ZNF185 (O15231)<br>AZGP1 (P25311)	

SEQUENCING STUDIES OF HPV 16 INTEGRATION SITES IN THE HUMAN GENOME			
**BIBLIOGRAPHIES**	**STUDY POPULATION**	**INTEGRATION SITES Protein (Uniprot code)**	**FINDINGS**
Multiplex Identification of Human Papillomavirus 16 DNA Integration Sites in Cervical Carcinomas<br><br>Xu *et al*., 2013	47 cervical carcinoma biopsies positive for HPV 16<br><br>**Sequencing studies of HPV 16 integration sites in the human genome**	CASZ1 (Q86V15)<br>GPN1 (Q9HCN4)<br>MBD5 (Q9P267)<br>ORC2 (Q13416)<br>PARD3B (Q8TEW8)<br>ERBB4 (Q15303)<br>Q7Z2X4 (Q7Z2X4)<br>USP4 (Q13107)<br>FHIT (P49789)<br>MECOM (Q03112)<br>ATP11B (Q9Y2G3)<br>CLDN1 (O95832)<br>CXCL8 (P10145)<br>MRPL1 (Q9BYD6)<br>C4orf17 (Q53FE4)<br>DUX4L2 (P0CJ85)<br>SLC29A1 (Q99808)<br>RUNX2 (Q13950)<br>CENPW (Q5EE01)<br>CREB5 (Q02930)<br>CSMD3 (Q7Z407)<br>POU5F1B (Q06416)<br>MYC (P01106)<br>IFT74 (Q96LB3)	A novel multiplex strategy for sequence determination of HPV16 DNA integration sites. It includes DNA fragmentation and adapter tagging, PCR enrichment of the HPV16 early region, Illumina next-generation sequencing, data processing, and validation of candidate integration sites by junction-PCR. This strategy was performed with 51 cervical cancer samples (47 primary tumors and 4 cell lines). Altogether 75 HPV16 integration sites (39-junctions) were identified and assigned to the individual samples. By comparing the DNA junctions with the presence of viral oncogene fusion transcripts, 44 tumors could be classified into four groups: Tumors with one transcriptionally active HPV16 integrate (n = 12), tumors with transcribed and silent DNA junctions (n = 8), tumors carrying episomal HPV16 DNA (n = 10), and tumors with one to six DNA junctions, but without fusion transcripts (n = 14).

		UBAP2 (Q5T6F2)<br>DENND1A (Q8TEH3)<br>USP6NL (Q92738)<br>LOXL4 (Q96JB6)<br>PYROXD2 (Q8N2H3)<br>PTPRJ (Q12913)<br>BCL2L14 (Q9BZR8)<br>KLF5 (Q13887)<br>KLF12 (Q9Y4X4)<br>HIF1A (Q16665)<br>LIPC (P11150)<br>SEMA4B (Q9NPR2)<br>STARD3 (Q14849)<br>ERBB2 (P04626)<br>VMP1 (Q96GC9)<br>FHOD3 (Q2V2M9)<br>ARID3B (Q8IVW6)<br>MOB3A (Q96BX8)<br>GATAD2A (Q86YP4)<br>URI1 (O94763)<br>MACROD2 (A1Z1Q3)<br>CBFA2T2 (O43439)<br>STS (P08842)<br>NHS (Q6T4R5)<br>SUPT20HL2 (P0C7V6)<br>PDK3 (Q15120)<br>DCAF12L2 (Q5VW00)<br>SLITRK2 (Q9H156)	

Genome-wide profiling of HPV integration in cervical cancer identifies clustered genomic hot spots and a potential microhomology-mediated integration mechanism<br><br>Hu *et al*., 2015	Cervical biopsies:<br>10 CIN I<br>9 CIN II<br>7 CIN III<br>89 SCCE<br>15 adenocarcinoma<br><br>3 cervical cancer cell lines: CaSki, HeLa y SiHa	AGTR2 (P50052)<br>DMD (P11532)<br>LRP1B (Q9NZR2)<br>CDH7 (Q9ULB5)<br>FHIT (P49789)<br>DCC (P43146)<br>CADM2 (Q8N3J6)<br>HS3ST4 (Q9Y661)<br>LEPREL1 (Q8IVL5)<br>KLF5 (Q13887)<br>KLF12 (Q9Y4X4)<br>HMGA2 (P52926)<br>M5X2 (P35548)<br>POU5F1B (Q06416)<br>SEMA3D (O95025)<br>C9orf85 (Q96MD7)<br>DLG2 (Q15700)<br>CPNE8 (Q86YQ8)<br>MYC (P01106)<br>MAGI1 (Q96QZ7)	By conducting whole-genome sequencing and high-throughput viral integration detection, we identified 3,667 HPV integration breakpoints in 26 cervical intraepithelial neoplasias, 1 04 cervical carcinomas and five cell lines. Beyond recalculating frequencies for the previously reported frequent integration sites POU5F1B (9.7%), FHIT (8.7%), KLF12 (7.8%), KLF5 (6.8%), LRP1B (5.8%) and LEPREL1 (4.9%), we discovered new hot spots HMGA2 (7.8%), DLG2 (4.9%) and SEMA3D (4.9%). Protein expression from FHIT and LRP1B was downregulated when HPV integrated in their introns. Protein expression from MYC and HMGA2 was elevated when HPV integrated into flanking regions.
Mechanistic signatures of HPV insertions in cervical carcinomas<br><br>Holmes *et al*., 2016	72 cervical carcinomas biopsies HR-HPV (16, 18, 33, 45, 51, 45, 68)	NUDT15 (Q9NV35)<br>MED4 (Q9NPJ6)<br>ITM2B (Q9Y287)<br>RB1 (P06400)<br>LPAR6 (P43657)<br>SUCLA2 (Q9P2R7)<br>OFD1 (O75665)<br>RAB9A (P51151)<br>EGFL6 (Q8IUX8)	Developed a generic and comprehensive Capture-HPV method followed by Next Generation Sequencing (NGS). Starting from biopsies or circulating DNA samples, this Capture-NGS approach rapidly identifies the HPV genotype, HPV status (integrated, episomal or absence), the viral-host DNA junctions and the associated genome rearrangements. This analysis of 72 cervical carcinomas identified five HPV signatures. The first

		GPM6B (Q13491)<br>ZBTB18 (Q99592)<br>AKT3 (Q9Y243)<br>C1ORF100 (Q5SVJ3)<br>RAB11A (P62491)<br>MEGF1 (Q9NYQ8)<br>MAPK10 (P53779)<br>PTPN13 (Q12923)<br>ARHGAP24 (Q8N264)<br>AFF1 (P51825)<br>MED13L (Q71F56)<br>RNFT2 (Q96EX2)<br>ZNF341 (Q9BYN7)<br>PXMP4 (Q9Y6I8)<br>NECAB3 (Q96P71)<br>CBFA2T2 (O43439)<br>RPL13A (P40429)<br>FLT3LG (P49771)<br>DHRS3 (O75911)<br>TBC1D22B (Q9NU19)<br>CPZ (Q66K79)<br>GPR78 (Q96P69)<br>TRMT44 (Q8IYL2)<br>ACOX3 (O15254)<br>HMX1 (Q9NP08)<br>AFF3 (P51826)<br>REV1 (Q9UBZ9)<br>KLF5 (Q13887)<br>KLF12 (Q9Y4X4)	two signatures contain two hybrid chromosomal–HPV junctions whose orientations are co-linear (2J-COL) or non-linear (2J-NL), revealing two modes of viral integration associated with chromosomal deletion or amplification events, respectively. The third and fourth signatures exhibit 3–12 hybrid junctions, either clustered in one locus (MJ-CL) or scattered at distinct loci (MJ-SC) while the fifth signature consists of episomal HPV genomes (EPI).

		PIBF1 (Q8WXW3)<br>POU5F1B (Q06416)<br>MYC (P01106)<br>PIBF1 (Q8WXW3)<br>CXCL6 (P80162)<br>PF4V1 (P10720)<br>IL8 (P10145)<br>CXCL1 (P09341)<br>RASSF6 (Q6ZTQ3)<br>PF4 (P02776)<br>MMP12 (P39900)<br>MMP3 (P08254)<br>IMMP2L (Q96T52)<br>AHR (P35869)<br>WDR20 (Q8TBZ3)<br>TUBD1 (Q9UJT1)<br>RNF152 (Q8N8N0)<br>PIGN (O95427)<br>TBC1D1 (Q86TI0)<br>TP63 (Q9H3D4)<br>LEPREL1 (Q8IVL5)<br>TPRG1 (Q6ZUI0)<br>ANXA1 (P04083)<br>HS3ST1 (O14792)<br>HES1 (Q14469)<br>SST (P61278)<br>RTP2 (Q5QGT7)<br>BCL6 (P41182)<br>LPP (Q93052)	

		VMP1 (Q96GC9)<br>MAST4 (O15021)<br>NFIX (Q14938)<br>NTMT1 (Q9BV86)<br>C9orf50 (Q5SZB4)<br>RAB22A (Q9UL26)<br>YIPF6 (Q96EC8)<br>PARD3B (Q8TEW8)<br>CENPW (Q5EE01)<br>C9ORF3 (Q8N6M6)<br>MACROD2 (A1Z1Q3)<br>ZRANB2 (O95218)<br>PTGER3 (P43115)<br>UGP2 (Q16851)<br>MDH1 (P40925)<br>VPS54 (Q9P1Q0)<br>WDPCP (O95876)<br>FAM135B (Q49AJ0)<br>DACH1 (Q9UI36)<br>ATP10A (O60312)<br>SPG11 (Q96JI7)<br>SORD (Q00796)<br>GPR137B (O60478)<br>ERO1LB (Q86YB8)<br>LYST (Q99698)<br>EDARADD (Q8WWZ3)<br>HM13 (Q8TCT9)<br>ID1 (P41134)	

		BCL2L1 (Q07817)<br>MAP2 (P11137)<br>COL4A4 (P53420)<br>GATSL1 (A6NHX0)<br>ZFPM2 (Q8WW38)<br>OXR1 (Q8N573)<br>EPN1 (Q9Y6I3)<br>TLE3 (Q04726)<br>UACA (Q9BZF9)<br>ATXN3L (Q9H3M9)<br>EGFL6 (Q8IUX8)<br>MAGI2 (Q86UL8)<br>MAGI1 (Q96QZ7)<br>LAMB3 (Q13751)	
The characteristics of HPV integration in cervical intraepithelial cells<br><br>Li *et al*., 2019	230 HPV positive histological diagnosis of CIN (1,2 and 3) and SCC	MC4R (P32245)<br>YTHDF1 (Q9BYJ9)<br>FRMPD4 (Q14CM0)<br>DIAPH2 (O60879)<br>ANKRD30BL (A7E2S9)<br>LRP1B (Q9NZR2)<br>NFE2L2 (Q16236)<br>MECOM (Q03112)<br>TP63 (Q9H3D4)<br>P3H2 (Q8IVL5)<br>NR4A3 (Q92570)<br>KLF5 (Q13887)	Deploying HIVID, a cost-effective technique to detect HPV integration sites, our team had studied the characteristics of HPV integrations in cervical exfoliated cells. Our results indicated that both the sample proportion and the number of HPV integrations gradually increased following the development of cervical lesion. Meanwhile, our data also revealed that there were recurrent genes integrated by HPV in cervical exfoliated cells. Collectively, the HPV integration breakpoints were highly enriched in the intron and promoter regions. Intriguingly, the gene pathway analysis indicated that the HPV-integrated genes were strongly inclined to pathways of metabolism of xenobiotics by

			cytochrome P450, chemical carcinogenesis and steroid hormone biosynthesis.
Genome-wide profiling of human papillomavirus DNA integration in liquid-based cytology specimens from a Gabonese female Population using HPV capture technology<br><br>Nkili-Meyong *et al.*, 2019	Liquid-based cytology<br>13 ASCUS<br>5 LSIL<br>7 HSIL<br>7 SCC	RRM1 (P23921)<br>STIM1 (Q13586)<br>KLF12 (Q9Y4X4)<br>GOLIM4 (O00461)<br>SERPINI1 (Q99574)<br>MYCN (P04198)<br>SCGB1A1 (P11684)<br>**AHNAK** (Q09666)<br>EEF1G (P26641)<br>TUT1 (Q9H6E5)<br>**MTA2** (O94776)<br>ROM1 (Q03395)<br>**EML3** (Q32P44)<br>**GANAB** (Q14697)<br>INTS5 (Q6P9B9)<br>CSKMT(A8MUP2)<br>BSCL2 (Q96G97)<br>HNRNPUL2 (Q1KMD3)<br>ZBTB3 (Q9H5J0)<br>TTC9C (Q8N5M4)<br>POLR2G (P62487)<br>HNRNPUL2 (Q1KMD3)<br>BSCL2 (Q96G97)<br>TAF6L (Q9Y6J9)	In this study, using a double-capture system followed by high-throughput sequencing, we determined the HPV integration status present in liquid-based cervical smears in an urban Gabonese population. The main inclusion criteria were based on cytological grade and the detection of the HPV16 genotype using molecular assays. The rate of HPV integration in the host genome varied with cytological grade: 85.7% (6/7), 71.4% (5/7), 66.7% (2/3) 60% (3/5) and 30.8% (4/13) for carcinomas, HSIL, ASCH, LSIL and ASCUS, respectively. For high cytological grades (carcinomas and HSIL), genotypes HPV16 and 18 represented 92.9% of the samples (13/14). The integrated form of HPV16 genotype was mainly found in high-grade lesions in 71.4% of samples regardless of cytological grade. Minority genotypes (HPV33, 51, 58 and 59) were found in LSIL samples, except HPV59, which was identified in one HSIL sample.

		NXF1 (Q9UBU9)<br>LBHD1 (Q9BQE6)<br>GNG3 (P63215)<br>TMEM223 (A0PJW6)<br>STX5 (Q13190)<br>TMEM179B (Q7Z7N9)<br>WDR74 (Q6RFH5)<br>WDR74 (Q04323)<br>UQCC3 (Q6UW78)<br>SLC3A2 (P08195)<br>TANC2 (Q9HCD6)<br>MARCHF10 (Q8NA82)<br>MRC2 (Q9UBG0)<br>KTN1 (Q86UP2)<br>FBXO34 (Q9NWN3)<br>WDHD1 (O75717)<br>SAMD4A (Q9UPU9)<br>GCH1 (P30793)<br>CGRRF1 (Q99675)<br>CDKN3 (Q16667)<br>BMP4 (P12644)<br>FERMT2 (Q96AC1)<br>DDHD1 (Q8NEL9)<br>STYX (Q8WUJ0)<br>PSMC6 (P62333)<br>GNPNAT1 (Q96EK6)<br>PTGDR (Q13258)<br>PELI2 (Q9HAT8)	

		TMEM260 (Q9NX78)<br>CENPW (Q5EE01)<br>TRMT11 (Q7Z4G4)<br>PDSS2 (Q86YH6)<br>BEND3 (Q5T5X7)<br>MTRES1 (Q9P0P8)<br>CHGB (P05060)<br>TRMT6 (Q9UJA5)<br>MCM8 (Q9UJA3)<br>GPCPD1 (Q9NPB8)<br>KLF12 (Q9Y4X4)<br>PXDN (Q92626)<br>MYT1L (Q9UL68)<br>SNTG2 (Q9NY99)<br>TPO (P07202)<br>KAT6B (Q8WYB5)<br>STC1 (P52823)<br>SIK1 (P57059)<br>HSF2BP (O75031)<br>H2BC12L (P57053)<br>CRYAA (P02489)<br>L3MBTL2 (Q969R5)<br>RANGAP1 (P46060)<br>ZC3H7B (Q9UGR2)<br>TEF (Q10587)<br>EP300 (Q09472)<br>RBX1 (P62877)<br>XPNPEP3 (Q9NQH7)<br>DPYSL3 (Q14195)	

		STK32A (Q8WU08)<br>PPP2R2B (Q00005)	
Accurate Detection of HPV Integration Sites in Cervical Cancer Samples Using the Nanopore MinION Sequencer Without Error Correction<br><br>Yang *et al.*, 2020	Cervical squamous cell carcinoma stage IIB VPH 16	CHMP4B (Q9H444)<br>RALY (Q9UKM9)<br>FBXW7-AS1 (B0L3A2)<br>CAGE1 (Q8TC20)<br>TFAP2A (P05549)<br>RPS6KA3 (P51812)<br>CNKSR2 (Q8WXI2)<br>CAGE1 (Q8TC20)<br>RPS6KA3 (P51812)<br>CNKSR2 (Q8WXI2)<br>CRACDL (Q6NV74)<br>CCDC150 (Q8NCX0)<br>EDN1 (P05305)<br>PHACTR1 (Q9C0D0)<br>PDGFD (Q9GZP0)<br>CCDC150 (Q8NCX0)<br>ELP4 (Q96EB1)<br>RPS6KA3 (P51812)<br>RREB1 (Q92766)<br>KLF12 (Q9Y4X4)<br>KIF3B (O15066)<br>ASXL1 (Q8IXJ9)<br>PDGFD (Q9GZP0)<br>STMND1 (H3BQB6)<br>OR4F3 (Q6IEY1)<br>TUBB8 (Q3ZCM7)<br>CNKSR2 (Q8WXI2)	This study, we evaluated the feasibility of identifying HPV integration sites by nanopore sequencer. Specifically, we re-sequenced the integration sites of a previously published sample by both nanopore and Illumina sequencing. After analyzing the results, three points of conclusions were drawn: first, 13 out of 19 previously published integration sites were found from all three datasets (i.e., nanopore, Illumina, and the published data), indicating a high overlap rate and comparability among the three platforms; second, our pipeline of nanopore and Illumina data identified 66 unique integration sites compared with previous published paper with 13 of them being verified by Sanger sequencing, indicating the higher integration sites detection sensitivity of our results compared with published data; third, we established a pipeline which could be used in HPV integration site detection by nanopore sequencing data without doing error correction analysis.

		PDCD2 (Q16342)<br>TP63 (Q9H3D4)<br>ATRN (O75882)<br>CAGE1 (Q8TC20)<br>PTPRQ (Q9UMZ3)<br>KNL1 (Q8NG31)<br>DYSF (O75923)<br>CYP26B1 (Q9NR63)<br>RBL1 (P28749)<br>MYF6 (P23409)<br>TRAF1 (Q13077)<br>DSCR8 (Q96T75)<br>DSCR10 (P59022)<br>MAF (O75444)<br>FMN2 (Q9NZ56)<br>HELZ (P42694)<br>GYPC (P04921)<br>CXCR6 (O00574)<br>FYCO1 (Q9BQS8)<br>ERC2 (O15083)<br>ASXL1 (Q8IXJ9)<br>VEPH1 (Q14D04)<br>CAGE1 (Q8TC20)<br>DCDC1 (M0R2J8)<br>CDH4 (P55283)<br>CPNE8 (Q86YQ8)<br>INTS7 (Q9NVH2)	

Human papilloma virus (HPV) integration signature in Cervical Cancer: identification of MACROD2 gene as HPV hot spot integration site<br><br>Kamal *et al.*, 2021	Different integration signatures were identified using HPV double capture followed by next-generation sequencing (NGS) in 272 CC patients from the BioRAIDs study [NCT02428842].	ABCC3 (O15438)<br>ACSL6 (Q9UKU0)<br>AGBL4 (Q5VU57)<br>AGPS (O00116)<br>AK5 (Q9Y6K8)<br>Q13023 (AKAP6)<br>ANKRD66 (B4E2M5)<br>ANTXR2 (P58335)<br>ARHGAP42 (A6NI28)<br>ARHGEF6 (Q15052)<br>ATP6AP2 (O75787)<br>ATRNL1 (Q5VV63)<br>AVL9 (Q8NBF6)<br>AZIN1 (O14977)<br>BCL11B (Q9C0K0)<br>BMP5 (P22003)<br>BRINP3 (Q76B58)<br>ABTB3 (A6QL63)<br>PHAF1 (Q9BSU1)<br>LDAH (Q9H6V9)<br>CFAP299 (Q6V702)<br>TBC1D32 (Q96NH3)<br>(CACYBP) Q9HB71<br>CADM2 (Q8N3J6)<br>CAMSAP2 (Q08AD1)<br>CARMIL1 (Q5VZK9)<br>CCDC25 (Q86WR0)<br>CCDC66 (A2RUB6)<br>CDH16 (O75309)	Episomal HPV was much less frequent in CC as compared to anal carcinoma ($p < 0.0001$). We identified >300 different HPV-chromosomal junctions (inter- or intra-genic). The most frequent integration site in CC was in MACROD2 gene followed by MIPOL1/TTC6 and TP63. HPV integration signatures were not associated with histological subtype, FIGO staging, treatment or PFS. HPVs were more frequently episomal in PIK3CA mutated tumours ($p = 0.023$). Viral integration type was dependent on HPV genotype ($p < 0.0001$); HPV18 and HPV45 being always integrated. High HPV copy number was associated with longer PFS ($p = 0.011$).

		CEACAM5 (P06731)<br>CEACAM6 (P40199)<br>CELF2 (O95319)<br>CENPJ (Q9HC77)<br>CEP170P1 (Q96L14)<br>CFAP69 (A5D8W1)<br>CFAP77 (Q6ZQR2)<br>CHST11 (Q9NPF2)<br>CHST2 (Q9Y4C5)<br>CLDN16 (Q9Y5I7)<br>CLPTM1L (Q96KA5)<br>CMAHP (Q9Y471)<br>COL24A1 (Q17RW2)<br>COL4A5 (P29400)<br>COMMD10 (Q9Y6G5)<br>CPA6 (Q8N4T0)<br>CREB5 (Q02930)<br>CREBBP (Q92793)<br>CSMD1 (Q96PZ7)<br>CUL4B (Q13620)<br>CXCL1 (P09341)<br>CXCL2 (P19875)<br>DAB1 (O75553)<br>DEGS2 (Q6QHC5)<br>DGKD (Q16760)<br>DIAPH2 (O60879)<br>DLEU1 (O43261)<br>DLG2 (Q15700)<br>DLGAP1 (O14490)	

		DPP6 (P42658)<br>DPYD (Q12882)<br>EEFSEC (P57772)<br>EHF (Q9NZC4)<br>EHHADH (Q08426)<br>EMILIN2 (Q9BXX0)<br>EN1 (Q05925)<br>ENOX1 (Q8TC92)<br>ENSA (O43768)<br>EPHA3 (P29320)<br>EPN1 (Q9Y6I3)<br>ERBB2 (P04626)<br>ERC2 (O15083)<br>ERICH2 (A1L162)<br>EXOSC8 (Q96B26)<br>EXOSC9 (Q06265)<br>FAM81A (Q8TBF8)<br>FEZF1 (A0PJY2)<br>RUSC1-AS1 (Q66K80)<br>FGFR3 (P22607)<br>FHIT (P49789)<br>FNDC3B (Q53EP0)<br>FOXA1 (P55317)<br>FRMD4A (Q9P2Q2)<br>GALNTL5 (Q7Z4T8)<br>GALNTL6 (Q49A17)<br>GFRA1 (P56159)<br>GFRA4 (Q9GZZ7)<br>GRM7 (Q14831)	

		GSE1 (Q14687)<br>GSG1L (Q6UXU4)<br>HDAC8 (Q9BY41)<br>HEATR4 (Q86WZ0)<br>HIVEP1 (P15822)<br>HNRNPM (P52272)<br>HRH1 (P35367)<br>HSF4 (Q9ULV5)<br>IL33 (O95760)<br>INSR (P06213)<br>ITGAL (P20701)<br>JAK3 (P52333)<br>KCNQ5 (Q9NR82)<br>KHDRBS2 (Q5VWX1)<br>SPIDR (Q14159)<br>KIF26B (Q2KJY2)<br>KLF12 (Q9Y4X4)<br>KLF17 (Q5JT82)<br>KMT5C (Q86Y97)<br>P3H2 (Q8IVL5)<br>LIG1 (P18858)<br>LINC-PINT (A0A455ZAR2)<br>NEURL3 (Q96EH8)<br>LPP (Q93052)<br>LRP1B (Q9NZR2)<br>LRSAM1 (Q6UWE0)<br>LYN (P07948)<br>LYSMD3 (Q7Z3D4)	

		MACROD2 (A1Z1Q3)<br>MAML2 (Q8IZL2)<br>MARCHF2 (Q9P0N8)<br>MDH1 (P40925)<br>MECOM (Q03112)<br>MEGF11 (A6BM72)<br>MID1 (O15344)<br>MIPOL1 (Q8TD10)<br>TTC6 (Q86TZ1)<br>MMP12 (P39900)<br>MROH5 (Q6ZUA9)<br>MYH11 (P35749)<br>MYO16 (Q9Y6X6)<br>MYOC (Q99972)<br>NAALADL2<br>(Q58DX5)<br>NBEAL1 (Q6ZS30)<br>NCALD (P61601)<br>NEURL1B (A8MQ27)<br>NFATC2IP (Q8NCF5)<br>NFE2L2 (Q16236)<br>NFIC (P08651)<br>NFRKB (Q6P4R8)<br>NLGN1 (Q8N2Q7)<br>NME7 (Q9Y5B8)<br>NOL11 (Q9H8H0)<br>NR3C2 (P08235)<br>NR5A1 (Q13285)<br>NRG1 (Q02297)	

		NRG2 (O14511)<br>NRIP1 (P48552)<br>NSD2 (O96028)<br>NSMCE2 (Q96MF7)<br>NSRP1 (Q9H0G5)<br>NUAK2 (Q9H093)<br>NUGGC (Q68CJ6)<br>ORC1 (Q13415)<br>PARG (Q86W56)<br>PCDH11X (Q9BZA7)<br>PCSK5 (Q92824)<br>PDE4B (Q07343)<br>PDE4D (Q08499)<br>PDGFD (Q9GZP0)<br>PDPN (Q86YL7)<br>PFKFB3 (Q16875)<br>PGAP3 (Q96FM1)<br>PHF20 (Q9BVI0)<br>PIBF1 (Q8WXW3)<br>PIGN (O95427)<br>PLCL1 (Q15111)<br>PLGRKT (Q9HBL7)<br>PLSCR5 (A0PG75)<br>PTEN (P60484)<br>PTPN13 (Q12923)<br>PTPRD (P23468)<br>RAB11A (P62491)<br>RAB33B (Q9H082)<br>RAD51B (O15315)	

		RBFOX1 (Q9NWB1)<br>RIMS2 (Q9UQ26)<br>RMI1 (Q9H9A7)<br>RSRC1 (Q96IZ7)<br>SAMD12 (Q8N8I0)<br>SBF2 (Q86WG5)<br>SCARA3 (Q6AZY7)<br>SGCD (Q92629)<br>SGCZ (Q96LD1)<br>SGK1 (O00141)<br>SH3BP4 (Q9P0V3)<br>SH3GL1 (Q99961)<br>SIK2 (Q9H0K1)<br>SLC10A6 (Q3KNW5)<br>SLC27A2 (O14975)<br>SLC6A18 (Q96N87)<br>SLC6A19 (Q695T7)<br>SLC6A3 (Q01959)<br>SLC6A9 (P48067)<br>SLC9A5 (Q14940)<br>SLIT3 (O75094)<br>SMAD3 (P84022)<br>SMCO1 (Q147U7)<br>SMYD3 (Q9H7B4)<br>SMYD2 (Q9NRG4)<br>SNRPA (P09012)<br>SNTA1 (Q13424)<br>SPARC (P09486)<br>SPATA6L (Q8N4H0)	

		SPIDR (Q14159)<br>SPINK5 (Q9NQ38)<br>SSPN (Q14714)<br>SSR1 (P43307)<br>STAC (Q99469)<br>STAP2 (Q9UGK3)<br>STEAP2 (Q8NFT2)<br>STK39 (Q9UEW8)<br>STS (P08842)<br>SUN2 (Q9UH99)<br>SUSD1 (Q6UWL2)<br>Q6UWL2 (SUSD1)<br>TANC1 (Q9C0D5)<br>TBC1D10A (Q9BXI6)<br>TERT (O14746)<br>TFEB (P19484)<br>TGFBRAP1 (Q8WUH2)<br>TMEM63B (Q5T3F8)<br>TP63 (Q9H3D4)<br>TRAP1 (Q12931)<br>TSHZ2 (Q9NRE2)<br>TUBD1 (Q9UJT1)<br>TUBGCP3 (Q96CW5)<br>TULP1 (O00294)<br>UBE2A (P49459)<br>UGCG (Q16739)<br>USP24 (Q9UPU5)<br>USP37 (Q86T82)	

		USP40 (Q9NVE5)<br>VPS13B (Q7Z7G8)<br>WDR20 (Q8TBZ3)<br>WNT5A (P41221)<br>WWOX (Q9NZC7)<br>ZBTB7C (A1YPR0)<br>ZFAND3 (Q9H8U3)<br>ZNF532 (Q9HCE3)<br>ZNF667 (Q5HYK9)<br>ZNF8 (P17098)<br>ZNF844 (Q08AG5)<br>CNBP (P62633)	
Multi-omics mapping of human papillomavirus integration sites illuminates novel cervical cancer target genes<br><br>Iden *et al*., 2021	Combining multi-omics data from The Cancer Genome Atlas with patient-matched long-read sequencing of HPV integration sites, we developed a strategy for using HPV integration events to identify and prioritise novel candidate ICC target genes (integration-detected genes (IDGs).	TCF7L1 (Q9HCS4)<br>TGOLN2 (O43493)<br>PTP4A3 (O75365)<br>MROH5 (Q6ZUA9)<br>CD9 (P21926)<br>PLEKHG6 (Q3KR16)<br>TNFRSF1A (P19438)<br>SCNN1A (P37088)<br>LTBR (P36941)<br>CD27 (P26842)<br>RUSC1-AS1 (Q66K80)<br>NOP2 (P46087)<br>TAOK3 (Q9H2K8)<br>SLC46A3 (Q7Z3Q1)<br>MTUS2 (Q5JR59)<br>ERBB2 (P04626)<br>MIEN1 (Q9BRT3)	PacBio data revealed 267 unique human-HPV breakpoints comprising 87 total integration events in eight tumours. Candidate IDGs were filtered based on the following criteria: (1) proximity to integration site, (2) clonal representation of integration event, (3) tumour-specific expression (Z-score) and (4) association with ICC survival. Four candidates prioritised based on their unknown function in ICC (BNC1, RSBN1, USP36 and TAOK3) exhibited oncogenic properties in cervical cancer cell lines. Further, annotation of integration events provided clues regarding potential mechanisms underlying altered IDG expression in both integrated and non-integrated ICC tumours

		IKZF3 (Q9UKT9)<br>ZPBP2 (Q6X784)<br>GSDMB (Q8TAX9)<br>GJC1 (P36383)<br>EFTUD2 (Q15029)<br>NMT1 (P30419)<br>PLCD3 (Q8N3E9)<br>HEXIM1 (O94992)<br>HEXIM2 (Q96MH2)<br>MAP3K14 (Q99558)<br>ARHGAP27 (Q6ZUM4)<br>SBF1 (O95248)<br>DCST2 (Q5T1A1)<br>MORN1 (Q5T089)<br>DCST2 (Q5T1A1)<br>EFNA3 (P52797)<br>EFNA1 (P20827)<br>RC3H1 (Q5TC82)<br>GCFC2 (P16383)<br>LRRTM4 (Q86VH4)<br>RBMS1 (P29558)<br>TANK (Q92844)<br>ICOS (Q9Y6W8)<br>GSX2 (Q9BZM3)<br>PDGFRA (P16234)<br>TMPRSS11F (Q6ZWK6)<br>HPSE (Q9Y251)<br>FAT1 (Q14517)	

		GUSBP1 (Q15486)<br>CDH9 (Q9ULB4)<br>ATP10B (O94823)<br>QKI (Q96PU8)<br>TYW1B (Q6NUM6)<br>VPS13B (Q7Z7G8)<br>PTPRD (P23468)<br>NELL2 (Q99435)<br>FBXW8 (Q8N3Y1)<br>GPC5 (P78333)<br>SYNE2 (Q8WXH0)<br>RAD51B (O15315)<br>RPS6KA5 (O75582)<br>DGLUCY (Q7Z3D6)<br>CDC42BPB (Q9Y5S2)<br>MEIS2 (O14770)<br>TMCO5A (Q8N6Q1)<br>EXD1 (Q8NHP7)<br>TMOD2 (Q9NZR1)<br>LEO1 (Q8WVC0)<br>MAPK6 (Q16659)<br>PDPK1 (O15530)<br>PRSS54 (Q6PEW0)<br>GINS3 (Q9BRX5)<br>NDRG4 (Q9ULP0)<br>KIF2B (Q8N4N8)<br>TOM1L1 (O75674)<br>PRKCA (P17252)<br>SLC39A11 (Q8N1S59	

		SEC14L1 (Q92503)<br>SS18 (Q15532)<br>PSMA8 (Q8TAA3)<br>ZNF492 (Q9P255)<br>ZNF99 (A8MXY4)<br>ZNF567 (Q8N184)<br>SRXN1 (Q9BYN0)<br>SCRT2 (Q9NQ03)<br>MAFB (Q9Y5Q3)<br>DOK5 (Q9P104)<br>ZC4H2 (Q9NQZ6)<br>PRR32 (B1ATL7)<br>ACTRT1 (Q8TDG2)<br>MAGEA3 (P43357)<br>CETN2 (P41208)<br>NSDHL (Q15738)<br>MAMLD1 (Q13495)<br>SDK1 (Q7Z5N4)<br>HELLS (Q9NRZ9)<br>RSBN1 (Q5VWQ0)<br>AP4B1 (Q9Y6B7)<br>FBXL18 (Q96ME1)<br>PPP1R3A (Q16821)<br>FOXP2 (O15409)<br>PLXNA1 (Q9UIW2)<br>BNC1 (Q01954)<br>SH3GL3 (Q99963)<br>CRYL1 (Q9Y2S2)<br>MRPL35 (Q9NZE8)	

		COL4A4 (P53420)<br>GLI4 (P10075)<br>DENND3 (A2RUS2)<br><br>SPSB2 (Q99619)<br>EMG1 (Q92979)<br>USP5 (P45974)<br>PHB2 (Q99623)<br>POLR1D (P0DPB5)<br>POMP (Q9Y244)<br>WIPF2 (Q8TF74)<br>RPL23 (P62829)<br>MPP2 (Q14168)<br>TYMP (P19971)<br>GADD45A (P24522)<br>SG20L2 (Q9H9L3)<br>SCAMP3 (O14828)<br>CHTOP (Q9Y3Y2)<br>LAMTOR2 (Q9Y2Q5)<br>EFNA4 (P52798)<br>RPS27 (P02818)<br>RPS27 (P42677)<br>BCNA(Q96GW7)<br>FDPS (P14324)<br>CKS1B (P61024)<br>TNN (Q9UQP3)<br>CSN1S1 (P47710)<br>LIFR (P42702)<br>HNRNPF (P52597)	

		PRRG3 (Q9BZD7)<br>PHTF1 (Q9UMS5)<br>TRIM33 (Q9UPN9)<br>NRAS (P01111)<br>RBAK (Q9NYW8)<br>RBAKDN (A6NC62)<br>ZNF277 (Q9NRM2)<br>ZXDC (Q2QGD7)<br>SNX4 (O95219)<br>ZNF148 (Q9UQR1)<br>ZNF592 (Q92610)<br>BNC1 (Q01954)<br>METTL23 (Q86XA0)<br>MFSD11 (O43934)<br>SLC26A11 (Q86WA9)<br>SGSH (P51688)<br>PGS1 (Q32NB8)<br>CANT1 (Q8WVQ1)<br>MGAT5B (Q3V5L5)<br>USP36 (Q9P275)<br>EIF4A3 (P38919)<br>RNF213 (Q63HN8)<br>CBX2 (Q14781)<br>JMJD6 (Q6NYC1)<br>LGALS3BP (Q08380)<br>SEPTIN9 (Q9UHD8)<br>RPTOR (Q8N122)<br>GAA (P10253)<br>SRSF2 (Q01130)	

		SOCS3 (O14543)<br>LRATD2 (Q96KN1)<br>LASP1 (Q14847)<br>KRT19 (P08727)<br>TOP2A (P11388)	
Long-read sequencing unveils high-resolution HPV integration and its oncogenic progression in cervical cancer<br>Zhou et al., 2022	16 tumor simples positives HPV 16 in stage (I-II B)	CAPN1 (P07384)<br>GBF1 (Q92538)<br>MACROD2 (A1Z1Q3)<br>SOX14 (O95416)<br>ALDH1A1 (P00352)<br>LENG8 (Q96PV6)<br>LENG9 (Q96B70)<br>CDC42 (P60953)<br>AKAP13 (Q12802)<br>HNF1B (P35680)<br>KLF5 (Q13887)<br>RPS6KA5 (O75582)<br>VMP1 (Q96GC9)	e further demonstrate that multiple clonal integration events are involved in the use of shared breakpoints, the induction of inter-chromosomal translocations and the formation of extrachromosomal circular virus-human hybrid structures. Combined with the corresponding RNA-seq data, we highlight LINC00290, LINC02500 and LENG9 as potential driver genes in cervical cancer. Finally, we reveal the spatial relationship of HPV integration and its various structural variations as well as their functional consequences in cervical cancer. These findings provide insight into HPV integration and its oncogenic progression in cervical cancer.

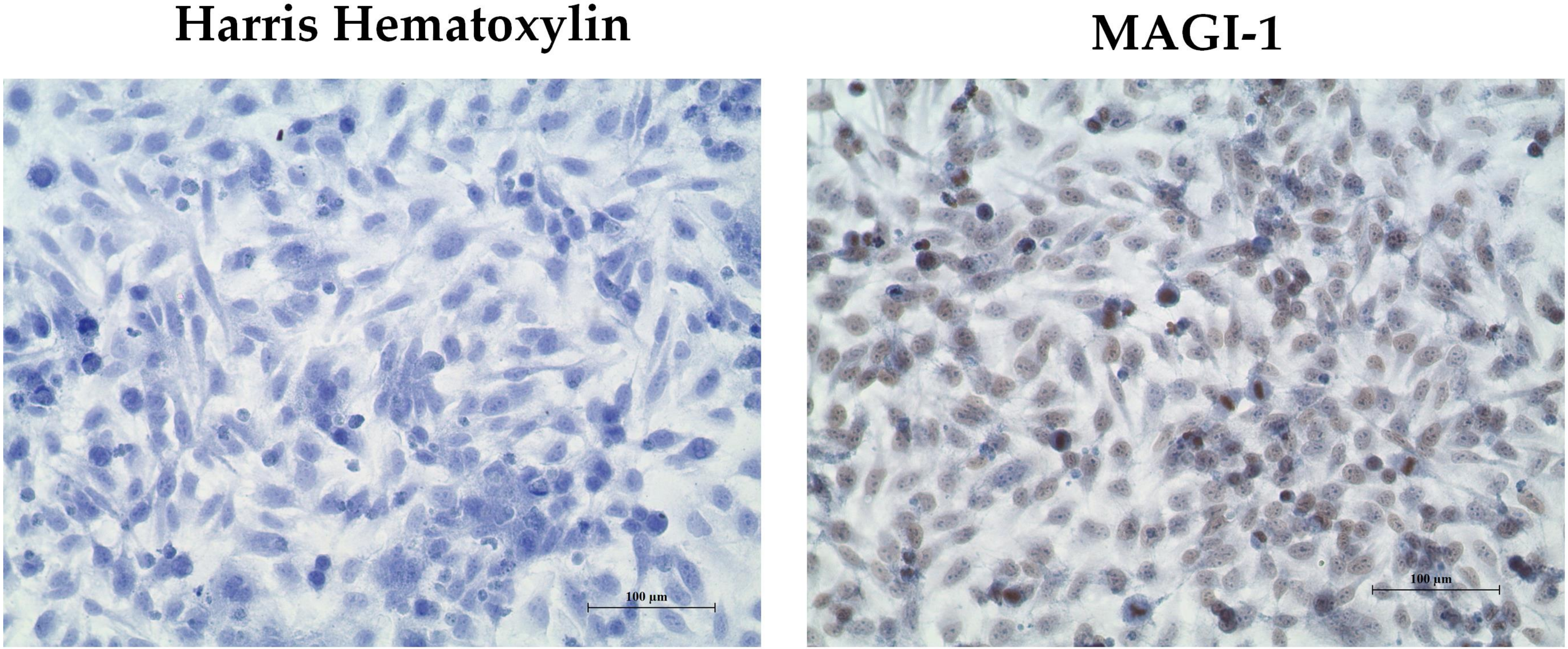

Figure S1. Evaluation of MAGI-1 expression in the HeLa cell line as a positive control of inmunocytochemistry

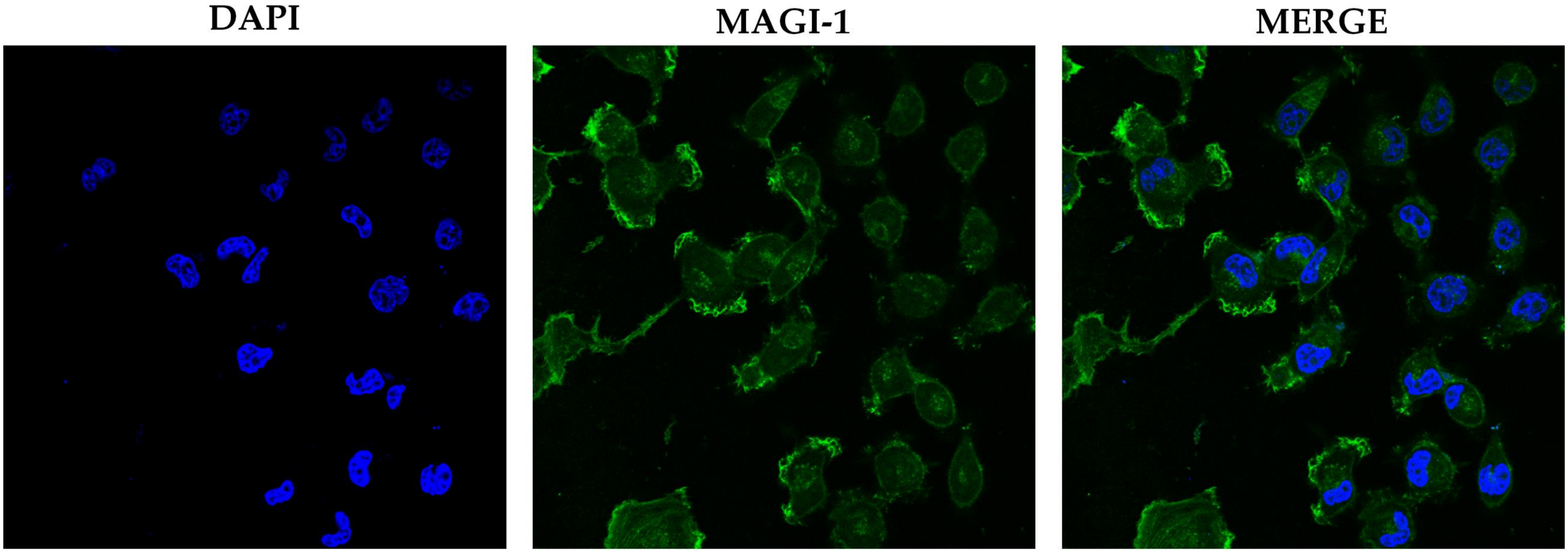

Figure S2. Evaluation of MAGI-1 by IF in HeLa cell line as a positive control of immunofluorescence.

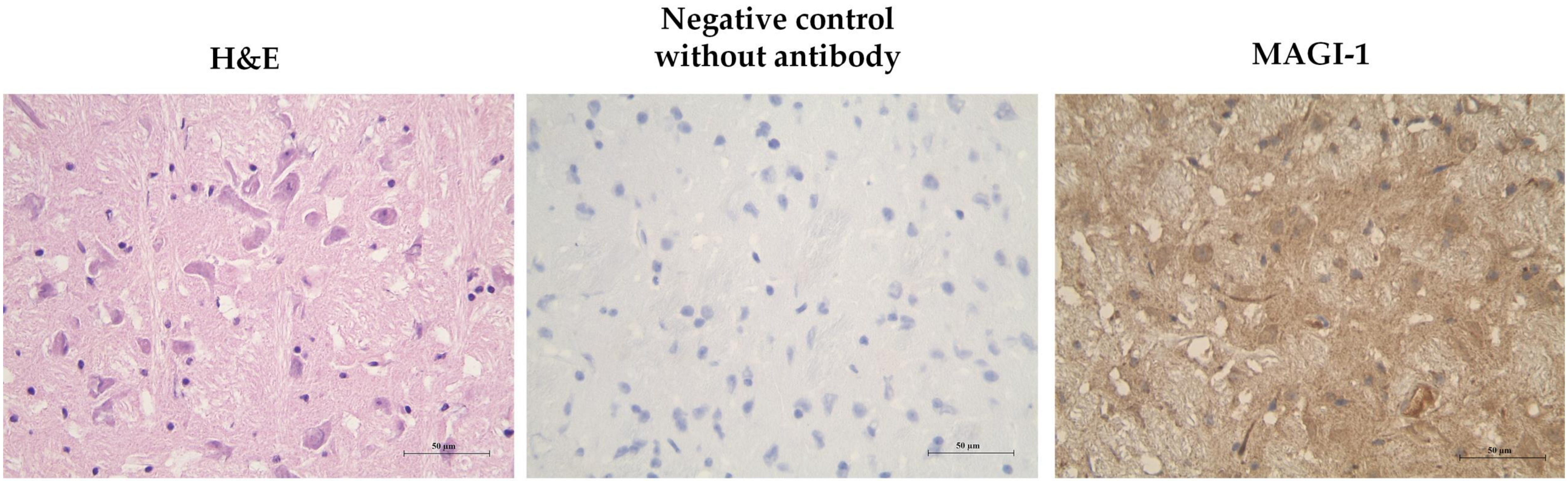

Figure S3. Evaluation of MAGI-1 expression in rat brain tissue as positive control of immunohistochemistry..

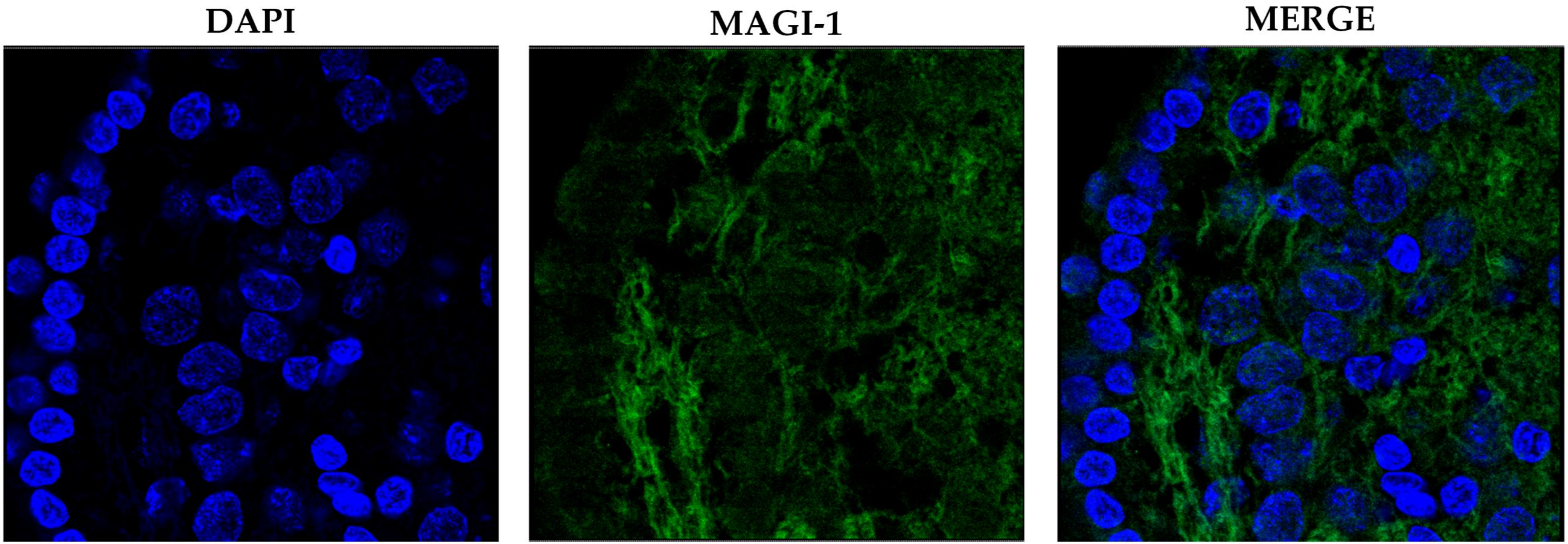

Figure S4. Evaluation of MAGI-1 by IF in HeLa rat brain tissue as positive control of immunohistofluorescence.
