# Supplementary figures and images for: Bioinformatics Analysis of Human Papillomavirus 16 Integration in Cervical Cancer: Changes in MAGI-1 Expression in Premalignant Lesions and Invasive Carcinoma
Source: Cancers (Basel). 2024 Jun 14;16(12):2225. doi: 10.3390/cancers16122225 (PMC11202195; doi:10.3390/cancers16122225)

# MAGI-1 expression (Total extract)

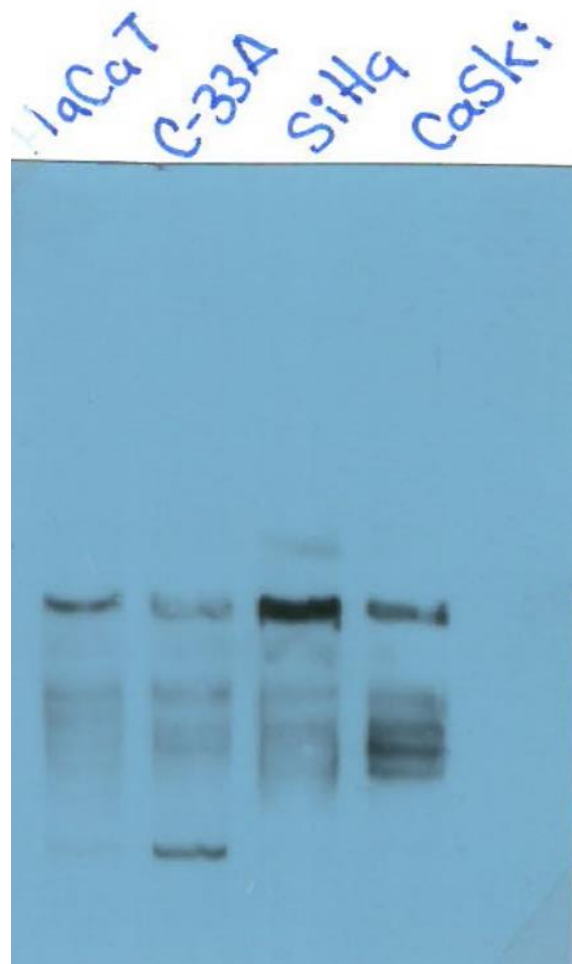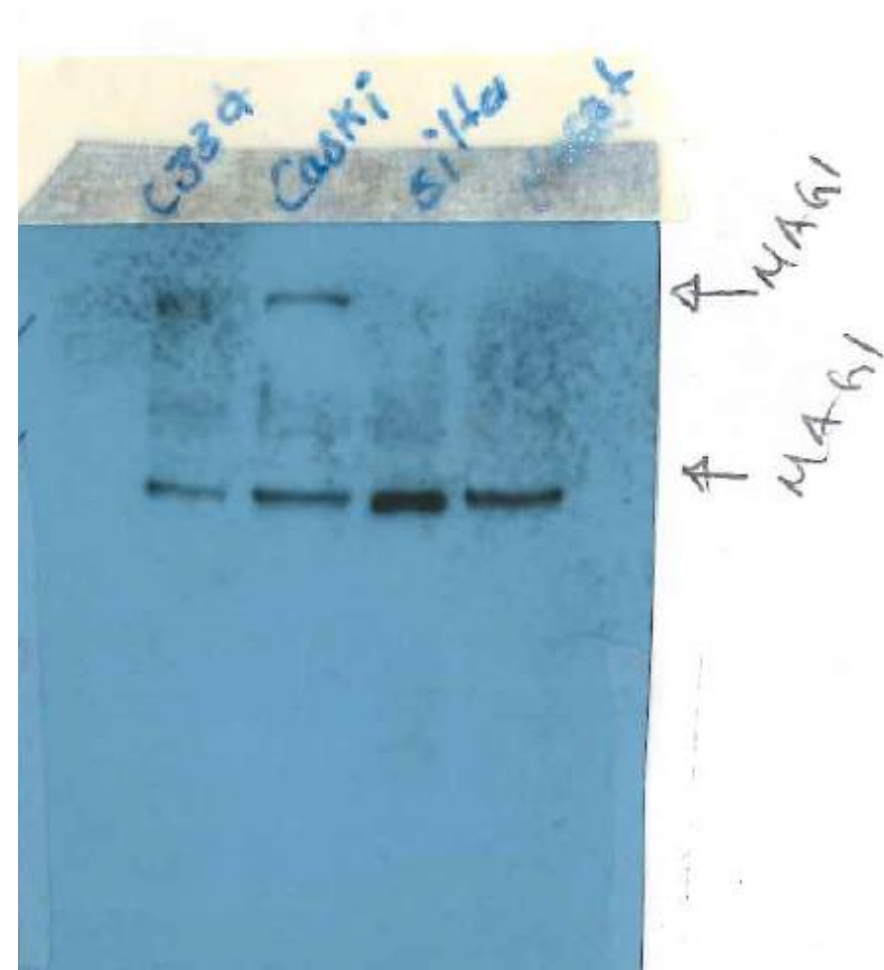

# Actin expression (total extract) load control

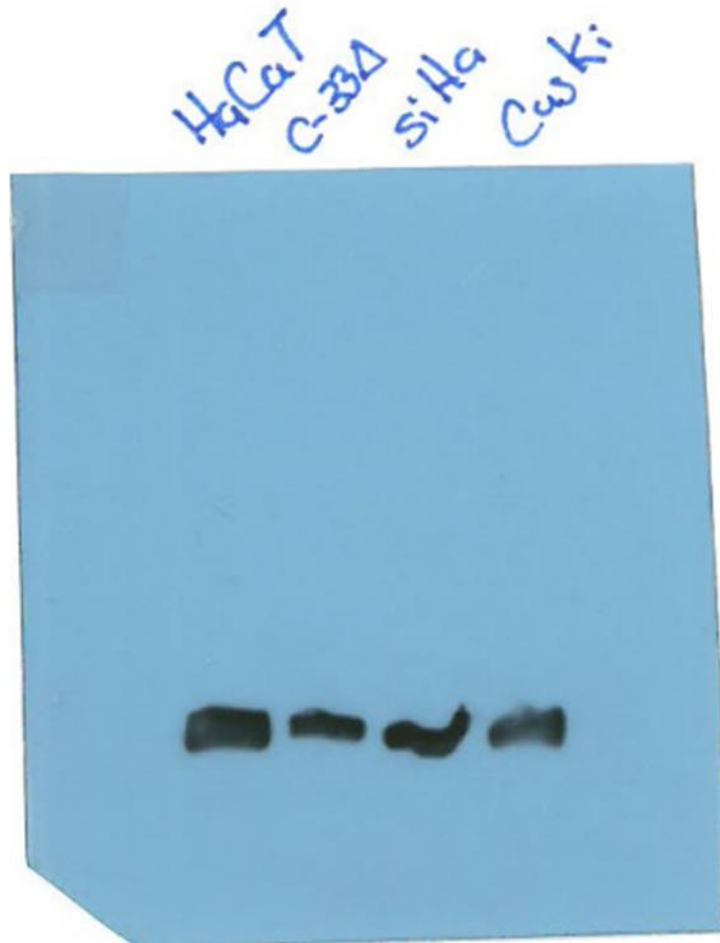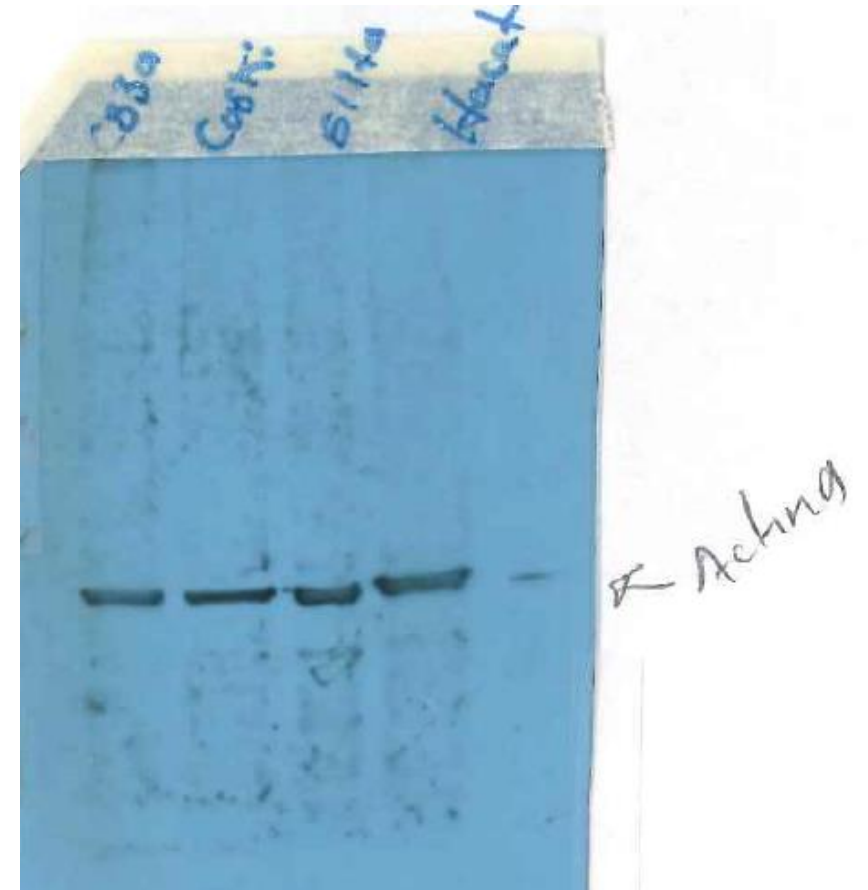

Supplement: Supplementary file 1 [file cancers-16-02225-s001.zip › cancers-2963458-supplementary/cancers-2963458-supplementary/cancers-2963458-original images of blots.pdf]
